# Supplementary material for: Variable Proportions of Phylogenetic Clustering and Low Levels of Antiviral Drug Resistance among the Major HBV Sub-Genotypes in the Middle East and North Africa
Source: Pathogens. 2021 Oct 15;10(10):1333. doi: 10.3390/pathogens10101333 (PMC8540944; doi:10.3390/pathogens10101333)
Supplement: Supplementary file 1 [file pathogens-1411491-supplementary.pdf › pathogens-1411491-supplementary.pdf]

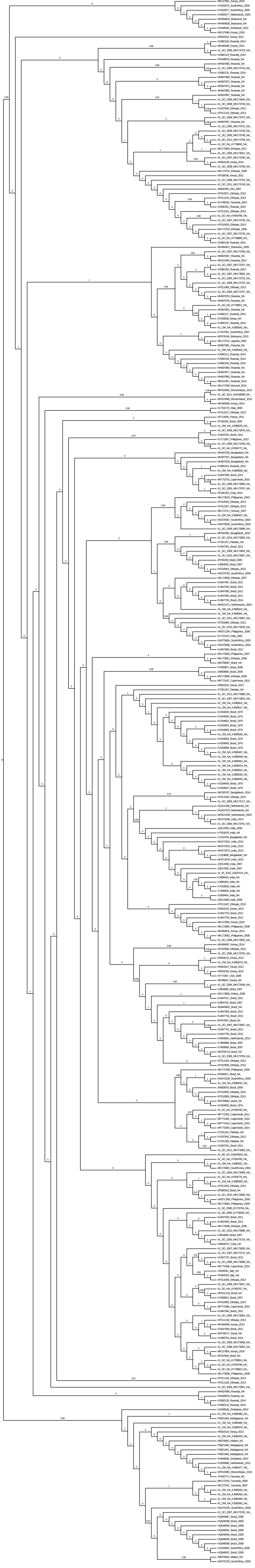

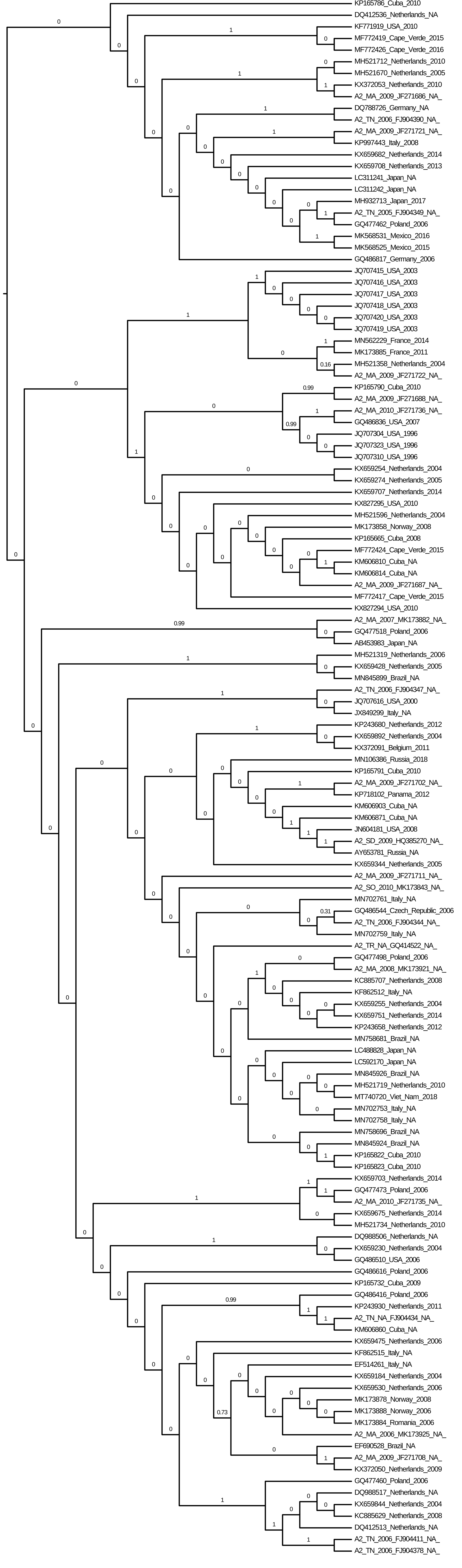
KP165786_Cuba_2010
DQ412536_Netherlands_NA
KF771919_USA_2010
MF772419_Cape_Verde_2015
MF772426_Cape_Verde_2016
MH521712_Netherlands_2010
MH521670_Netherlands_2005
KX372053_Netherlands_2010
A2_MA_2009_JF271686_NA_
DQ788726_Germany_NA
A2_TN_2006_FJ904390_NA_
A2_MA_2009_JF271721_NA_
KP997443_Italy_2008
KX659682_Netherlands_2014
KX659708_Netherlands_2013
LC311241_Japan_NA
LC311242_Japan_NA
MH932713_Japan_2017
A2_TN_2005_FJ904349_NA_
GQ477462_Poland_2006
MK568531_Mexico_2016
MK568525_Mexico_2015
GQ486817_Germany_2006
JQ707415_USA_2003
JQ707416_USA_2003
JQ707417_USA_2003
JQ707418_USA_2003
JQ707420_USA_2003
JQ707419_USA_2003
MN562229_France_2014
MK173885_France_2011
MH521358_Netherlands_2004
A2_MA_2009_JF271722_NA_
KP165790_Cuba_2010
A2_MA_2009_JF271688_NA_
A2_MA_2010_JF271736_NA_
GQ486836_USA_2007
JQ707304_USA_1996
JQ707323_USA_1996
JQ707310_USA_1996
KX659254_Netherlands_2004
KX659274_Netherlands_2005
KX659707_Netherlands_2014
KX827295_USA_2010
MH521596_Netherlands_2004
MK173858_Norway_2008
KP165665_Cuba_2008
MF772424_Cape_Verde_2015
KM606810_Cuba_NA
KM606814_Cuba_NA
A2_MA_2009_JF271687_NA_
MF772417_Cape_Verde_2015
KX827294_USA_2010
A2_MA_2007_MK173882_NA_
GQ477518_Poland_2006
AB453983_Japan_NA
MH521319_Netherlands_2006
KX659428_Netherlands_2005
MN845899_Brazil_NA
A2_TN_2006_FJ904347_NA_
JQ707616_USA_2000
JX849299_Italy_NA
KP243680_Netherlands_2012
KX659892_Netherlands_2004
KX372091_Belgium_2011
MN106386_Russia_2018
KP165791_Cuba_2010
A2_MA_2009_JF271702_NA_
KP718102_Panama_2012
KM606903_Cuba_NA
KM606871_Cuba_NA
JN604181_USA_2008
A2_SD_2009_HQ385270_NA_
AY653781_Russia_NA
KX659344_Netherlands_2005
A2_MA_2009_JF271711_NA_
A2_SO_2010_MK173843_NA_
MN702761_Italy_NA
GQ486544_Czech_Republic_2006
A2_TN_2006_FJ904344_NA_
MN702759_Italy_NA
A2_TR_NA_GQ414522_NA_
GQ477498_Poland_2006
A2_MA_2008_MK173921_NA_
KC885707_Netherlands_2008
KF862512_Italy_NA
KX659255_Netherlands_2004
KX659751_Netherlands_2014
KP243658_Netherlands_2012
MN758681_Brazil_NA
LC488828_Japan_NA
LC592170_Japan_NA
MN845926_Brazil_NA
MH521719_Netherlands_2010
MT740720_Viet_Nam_2018
MN702753_Italy_NA
MN702758_Italy_NA
MN758696_Brazil_NA
MN845924_Brazil_NA
KP165822_Cuba_2010
KP165823_Cuba_2010
KX659703_Netherlands_2014
GQ477473_Poland_2006
A2_MA_2010_JF271735_NA_
KX659675_Netherlands_2014
MH521734_Netherlands_2010
DQ988506_Netherlands_NA
KX659230_Netherlands_2004
GQ486510_USA_2006
GQ486616_Poland_2006
KP165732_Cuba_2009
GQ486416_Poland_2006
KP243930_Netherlands_2011
A2_TN_NA_FJ904434_NA_
KM606860_Cuba_NA
KX659475_Netherlands_2006
KF862515_Italy_NA
EF514261_Italy_NA
KX659184_Netherlands_2004
KX659530_Netherlands_2006
MK173878_Norway_2008
MK173888_Norway_2006
MK173884_Romania_2006
A2_MA_2006_MK173925_NA_
EF690528_Brazil_NA
A2_MA_2009_JF271708_NA_
KX372050_Netherlands_2009
GQ477460_Poland_2006
DQ988517_Netherlands_NA
KX659844_Netherlands_2004
KC885629_Netherlands_2008
DQ412513_Netherlands_NA
A2_TN_2006_FJ904411_NA_
A2_TN_2006_FJ904378_NA_

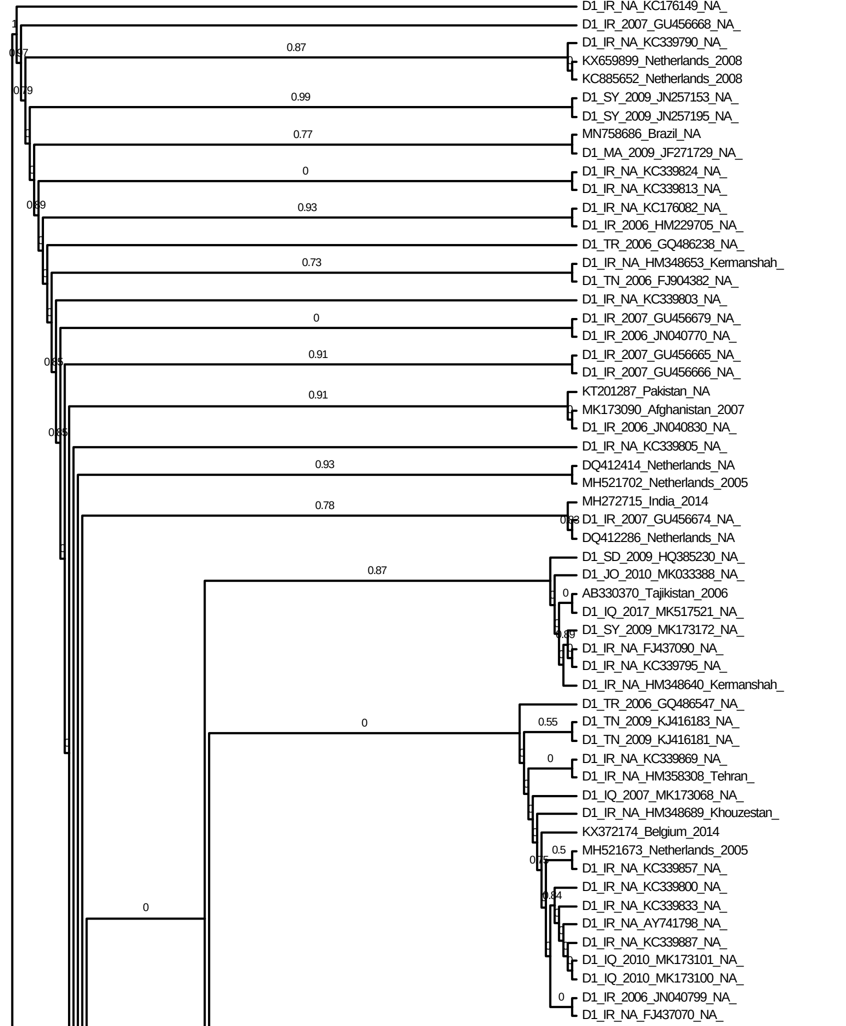
D1_IR_NA_KC176149_NA_
1
D1_IR_2007_GU456668_NA_
0.97
0.87
D1_IR_NA_KC339790_NA_
0
KX659899_Netherlands_2008
KC885652_Netherlands_2008
0.79
0.99
D1_SY_2009_JN257153_NA_
D1_SY_2009_JN257195_NA_
0.77
MN758686_Brazil_NA
D1_MA_2009_JF271729_NA_
0
D1_IR_NA_KC339824_NA_
D1_IR_NA_KC339813_NA_
0.89
0.93
D1_IR_NA_KC176082_NA_
D1_IR_2006_HM229705_NA_
D1_TR_2006_GQ486238_NA_
0.73
D1_IR_NA_HM348653_Kermanshah_
D1_TN_2006_FJ904382_NA_
D1_IR_NA_KC339803_NA_
0
D1_IR_2007_GU456679_NA_
D1_IR_2006_JN040770_NA_
0.85
0.91
D1_IR_2007_GU456665_NA_
D1_IR_2007_GU456666_NA_
0.91
KT201287_Pakistan_NA
0
MK173090_Afghanistan_2007
D1_IR_2006_JN040830_NA_
0.85
D1_IR_NA_KC339805_NA_
0.93
DQ412414_Netherlands_NA
MH521702_Netherlands_2005
0.78
MH272715_India_2014
0.83
D1_IR_2007_GU456674_NA_
DQ412286_Netherlands_NA
0.87
D1_SD_2009_HQ385230_NA_
D1_JO_2010_MK033388_NA_
0
AB330370_Tajikistan_2006
D1_IQ_2017_MK517521_NA_
D1_SY_2009_MK173172_NA_
0.89
D1_IR_NA_FJ437090_NA_
D1_IR_NA_KC339795_NA_
D1_IR_NA_HM348640_Kermanshah_
0
D1_TR_2006_GQ486547_NA_
0.55
D1_TN_2009_KJ416183_NA_
D1_TN_2009_KJ416181_NA_
0
D1_IR_NA_KC339869_NA_
D1_IR_NA_HM358308_Tehran_
D1_IQ_2007_MK173068_NA_
D1_IR_NA_HM348689_Khouzestan_
KX372174_Belgium_2014
0.5
MH521673_Netherlands_2005
0.75
D1_IR_NA_KC339857_NA_
D1_IR_NA_KC339800_NA_
0.84
D1_IR_NA_KC339833_NA_
D1_IR_NA_AY741798_NA_
D1_IR_NA_KC339887_NA_
D1_IQ_2010_MK173101_NA_
D1_IQ_2010_MK173100_NA_
0
D1_IR_2006_JN040799_NA_
D1_IR_NA_FJ437070_NA_

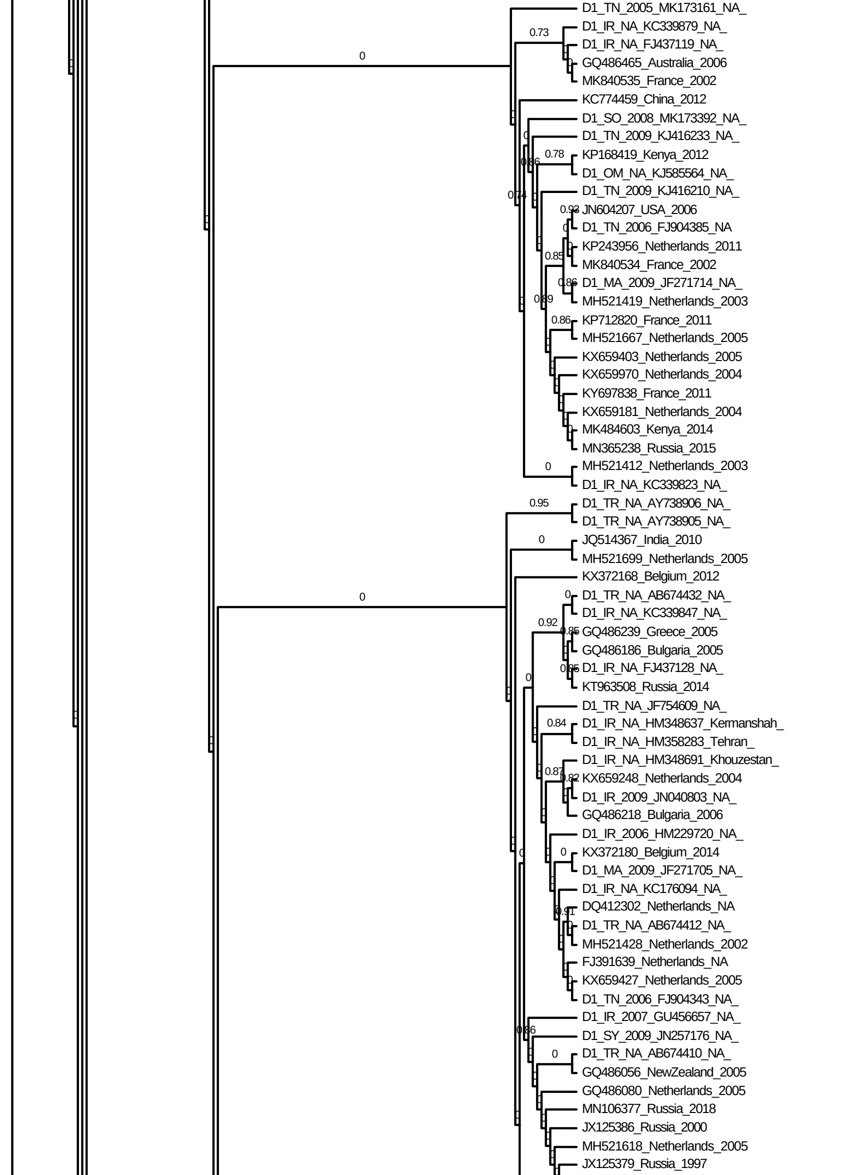
D1_TN_2005_MK173161_NA_
D1_IR_NA_KC339879_NA_
D1_IR_NA_FJ437119_NA_
GQ486465_Australia_2006
MK840535_France_2002
KC774459_China_2012
D1_SO_2008_MK173392_NA_
D1_TN_2009_KJ416233_NA_
KP168419_Kenya_2012
D1_OM_NA_KJ585564_NA_
D1_TN_2009_KJ416210_NA_
JN604207_USA_2006
D1_TN_2006_FJ904385_NA
KP243956_Netherlands_2011
MK840534_France_2002
D1_MA_2009_JF271714_NA_
MH521419_Netherlands_2003
KP712820_France_2011
MH521667_Netherlands_2005
KX659403_Netherlands_2005
KX659970_Netherlands_2004
KY697838_France_2011
KX659181_Netherlands_2004
MK484603_Kenya_2014
MN365238_Russia_2015
MH521412_Netherlands_2003
D1_IR_NA_KC339823_NA_
D1_TR_NA_AY738906_NA_
D1_TR_NA_AY738905_NA_
JQ514367_India_2010
MH521699_Netherlands_2005
KX372168_Belgium_2012
D1_TR_NA_AB674432_NA_
D1_IR_NA_KC339847_NA_
GQ486239_Greece_2005
GQ486186_Bulgaria_2005
D1_IR_NA_FJ437128_NA_
KT963508_Russia_2014
D1_TR_NA_JF754609_NA_
D1_IR_NA_HM348637_Kermanshah_
D1_IR_NA_HM358283_Tehran_
D1_IR_NA_HM348691_Khouzestan_
KX659248_Netherlands_2004
D1_IR_2009_JN040803_NA_
GQ486218_Bulgaria_2006
D1_IR_2006_HM229720_NA_
KX372180_Belgium_2014
D1_MA_2009_JF271705_NA_
D1_IR_NA_KC176094_NA_
DQ412302_Netherlands_NA
D1_TR_NA_AB674412_NA_
MH521428_Netherlands_2002
FJ391639_Netherlands_NA
KX659427_Netherlands_2005
D1_TN_2006_FJ904343_NA_
D1_IR_2007_GU456657_NA_
D1_SY_2009_JN257176_NA_
D1_TR_NA_AB674410_NA_
GQ486056_NewZealand_2005
GQ486080_Netherlands_2005
MN106377_Russia_2018
JX125386_Russia_2000
MH521618_Netherlands_2005
JX125379_Russia_1997

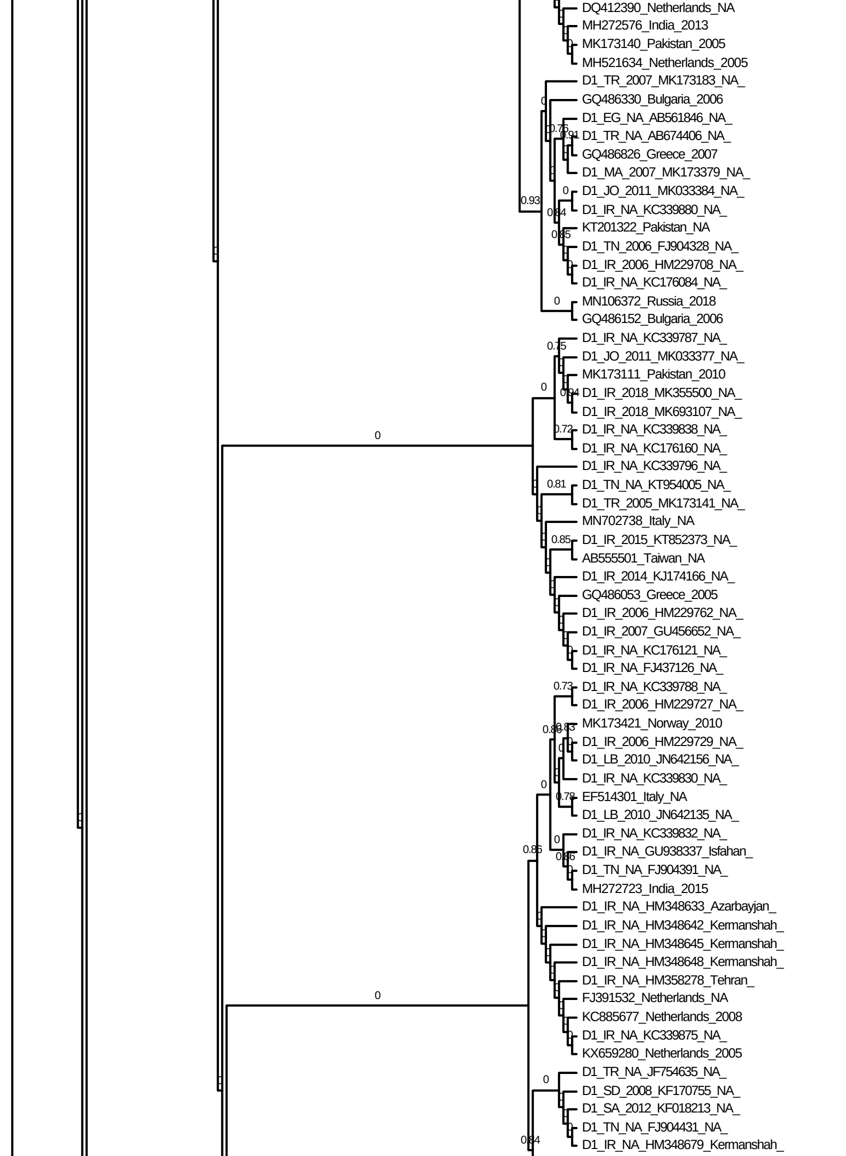

DQ412390_Netherlands_NA
MH272576_India_2013
MK173140_Pakistan_2005
MH521634_Netherlands_2005
D1_TR_2007_MK173183_NA_
GQ486330_Bulgaria_2006
D1_EG_NA_AB561846_NA_
D1_TR_NA_AB674406_NA_
GQ486826_Greece_2007
D1_MA_2007_MK173379_NA_
D1_JO_2011_MK033384_NA_
D1_IR_NA_KC339880_NA_
KT201322_Pakistan_NA
D1_TN_2006_FJ904328_NA_
D1_IR_2006_HM229708_NA_
D1_IR_NA_KC176084_NA_
MN106372_Russia_2018
GQ486152_Bulgaria_2006
D1_IR_NA_KC339787_NA_
D1_JO_2011_MK033377_NA_
MK173111_Pakistan_2010
D1_IR_2018_MK355500_NA_
D1_IR_2018_MK693107_NA_
D1_IR_NA_KC339838_NA_
D1_IR_NA_KC176160_NA_
D1_IR_NA_KC339796_NA_
D1_TN_NA_KT954005_NA_
D1_TR_2005_MK173141_NA_
MN702738_Italy_NA
D1_IR_2015_KT852373_NA_
AB555501_Taiwan_NA
D1_IR_2014_KJ174166_NA_
GQ486053_Greece_2005
D1_IR_2006_HM229762_NA_
D1_IR_2007_GU456652_NA_
D1_IR_NA_KC176121_NA_
D1_IR_NA_FJ437126_NA_
D1_IR_NA_KC339788_NA_
D1_IR_2006_HM229727_NA_
MK173421_Norway_2010
D1_IR_2006_HM229729_NA_
D1_LB_2010_JN642156_NA_
D1_IR_NA_KC339830_NA_
EF514301_Italy_NA
D1_LB_2010_JN642135_NA_
D1_IR_NA_KC339832_NA_
D1_IR_NA_GU938337_Isfahan_
D1_TN_NA_FJ904391_NA_
MH272723_India_2015
D1_IR_NA_HM348633_Azarbayjan_
D1_IR_NA_HM348642_Kermanshah_
D1_IR_NA_HM348645_Kermanshah_
D1_IR_NA_HM348648_Kermanshah_
D1_IR_NA_HM358278_Tehran_
FJ391532_Netherlands_NA
KC885677_Netherlands_2008
D1_IR_NA_KC339875_NA_
KX659280_Netherlands_2005
D1_TR_NA_JF754635_NA_
D1_SD_2008_KF170755_NA_
D1_SA_2012_KF018213_NA_
D1_TN_NA_FJ904431_NA_
D1_IR_NA_HM348679_Kermanshah_

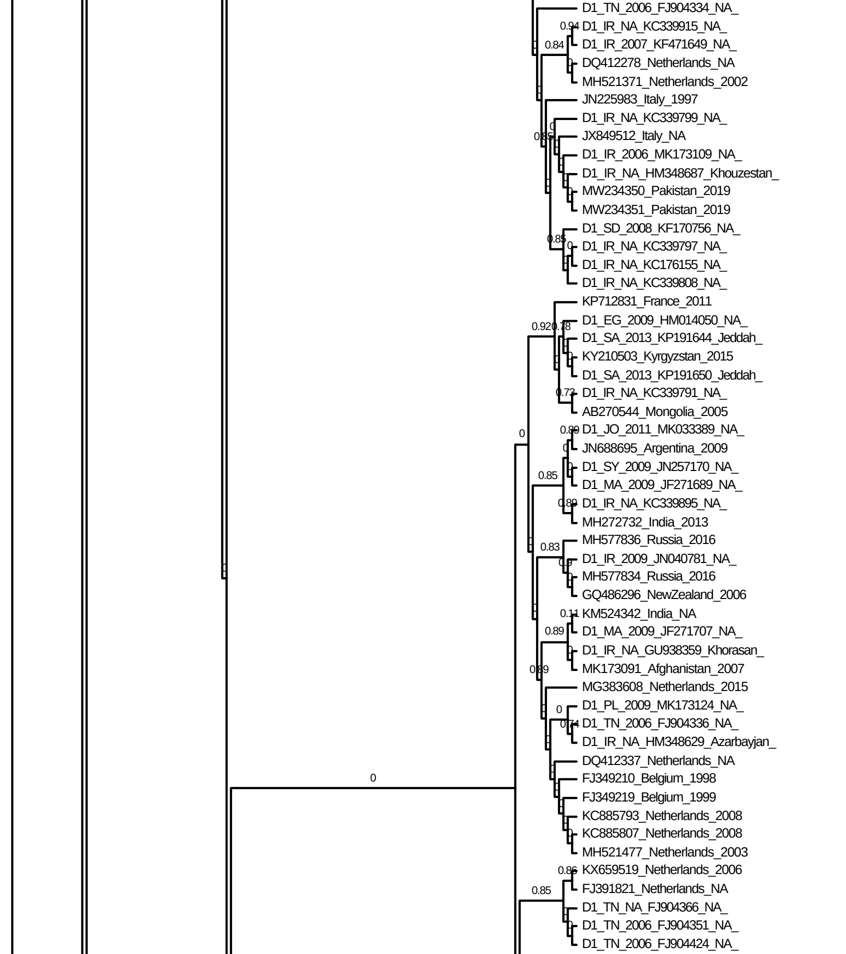
D1_TN_2006_FJ904334_NA_
D1_IR_NA_KC339915_NA_
D1_IR_2007_KF471649_NA_
DQ412278_Netherlands_NA
MH521371_Netherlands_2002
JN225983_Italy_1997
D1_IR_NA_KC339799_NA_
JX849512_Italy_NA
D1_IR_2006_MK173109_NA_
D1_IR_NA_HM348687_Khouzestan_
MW234350_Pakistan_2019
MW234351_Pakistan_2019
D1_SD_2008_KF170756_NA_
D1_IR_NA_KC339797_NA_
D1_IR_NA_KC176155_NA_
D1_IR_NA_KC339808_NA_
KP712831_France_2011
D1_EG_2009_HM014050_NA_
D1_SA_2013_KP191644_Jeddah_
KY210503_Kyrgyzstan_2015
D1_SA_2013_KP191650_Jeddah_
D1_IR_NA_KC339791_NA_
AB270544_Mongolia_2005
D1_JO_2011_MK033389_NA_
JN688695_Argentina_2009
D1_SY_2009_JN257170_NA_
D1_MA_2009_JF271689_NA_
D1_IR_NA_KC339895_NA_
MH272732_India_2013
MH577836_Russia_2016
D1_IR_2009_JN040781_NA_
MH577834_Russia_2016
GQ486296_NewZealand_2006
KM524342_India_NA
D1_MA_2009_JF271707_NA_
D1_IR_NA_GU938359_Khorasan_
MK173091_Afghanistan_2007
MG383608_Netherlands_2015
D1_PL_2009_MK173124_NA_
D1_TN_2006_FJ904336_NA_
D1_IR_NA_HM348629_Azarbayjan_
DQ412337_Netherlands_NA
FJ349210_Belgium_1998
FJ349219_Belgium_1999
KC885793_Netherlands_2008
KC885807_Netherlands_2008
MH521477_Netherlands_2003
KX659519_Netherlands_2006
FJ391821_Netherlands_NA
D1_TN_NA_FJ904366_NA_
D1_TN_2006_FJ904351_NA_
D1_TN_2006_FJ904424_NA_

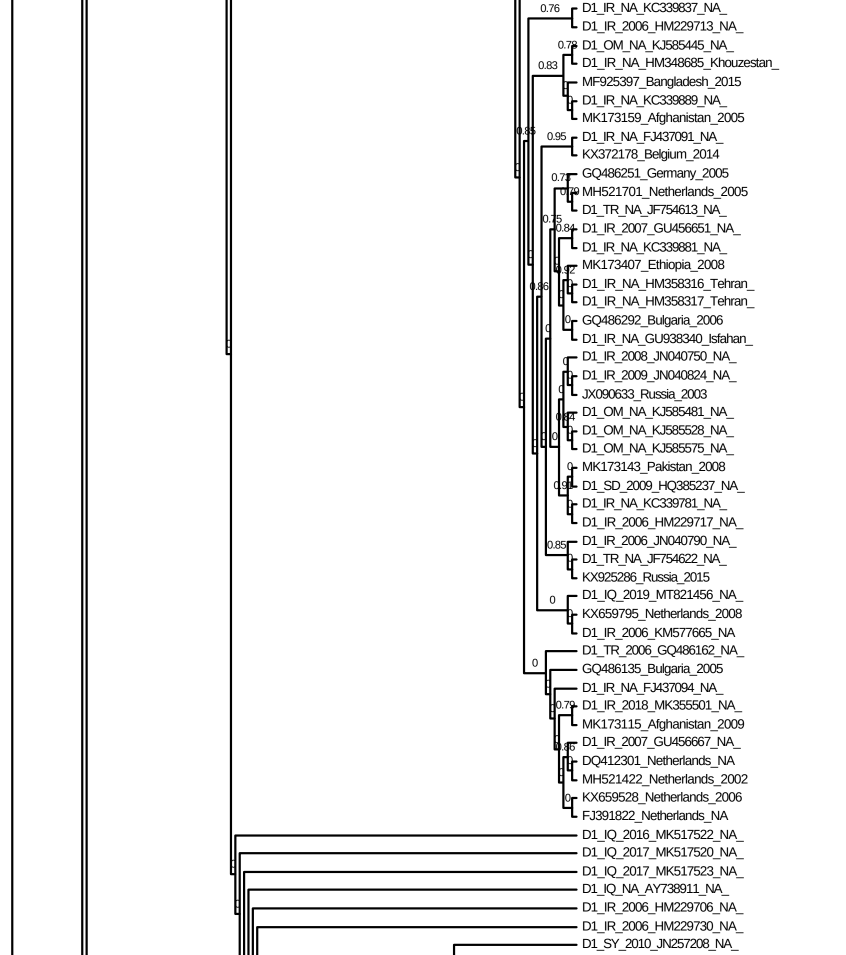
D1_IR_NA_KC339837_NA_
D1_IR_2006_HM229713_NA_
D1_OM_NA_KJ585445_NA_
D1_IR_NA_HM348685_Khouzestan_
MF925397_Bangladesh_2015
D1_IR_NA_KC339889_NA_
MK173159_Afghanistan_2005
D1_IR_NA_FJ437091_NA_
KX372178_Belgium_2014
GQ486251_Germany_2005
MH521701_Netherlands_2005
D1_TR_NA_JF754613_NA_
D1_IR_2007_GU456651_NA_
D1_IR_NA_KC339881_NA_
MK173407_Ethiopia_2008
D1_IR_NA_HM358316_Tehran_
D1_IR_NA_HM358317_Tehran_
GQ486292_Bulgaria_2006
D1_IR_NA_GU938340_Isfahan_
D1_IR_2008_JN040750_NA_
D1_IR_2009_JN040824_NA_
JX090633_Russia_2003
D1_OM_NA_KJ585481_NA_
D1_OM_NA_KJ585528_NA_
D1_OM_NA_KJ585575_NA_
MK173143_Pakistan_2008
D1_SD_2009_HQ385237_NA_
D1_IR_NA_KC339781_NA_
D1_IR_2006_HM229717_NA_
D1_IR_2006_JN040790_NA_
D1_TR_NA_JF754622_NA_
KX925286_Russia_2015
D1_IQ_2019_MT821456_NA_
KX659795_Netherlands_2008
D1_IR_2006_KM577665_NA
D1_TR_2006_GQ486162_NA_
GQ486135_Bulgaria_2005
D1_IR_NA_FJ437094_NA_
D1_IR_2018_MK355501_NA_
MK173115_Afghanistan_2009
D1_IR_2007_GU456667_NA_
DQ412301_Netherlands_NA
MH521422_Netherlands_2002
KX659528_Netherlands_2006
FJ391822_Netherlands_NA
D1_IQ_2016_MK517522_NA_
D1_IQ_2017_MK517520_NA_
D1_IQ_2017_MK517523_NA_
D1_IQ_NA_AY738911_NA_
D1_IR_2006_HM229706_NA_
D1_IR_2006_HM229730_NA_
D1_SY_2010_JN257208_NA_

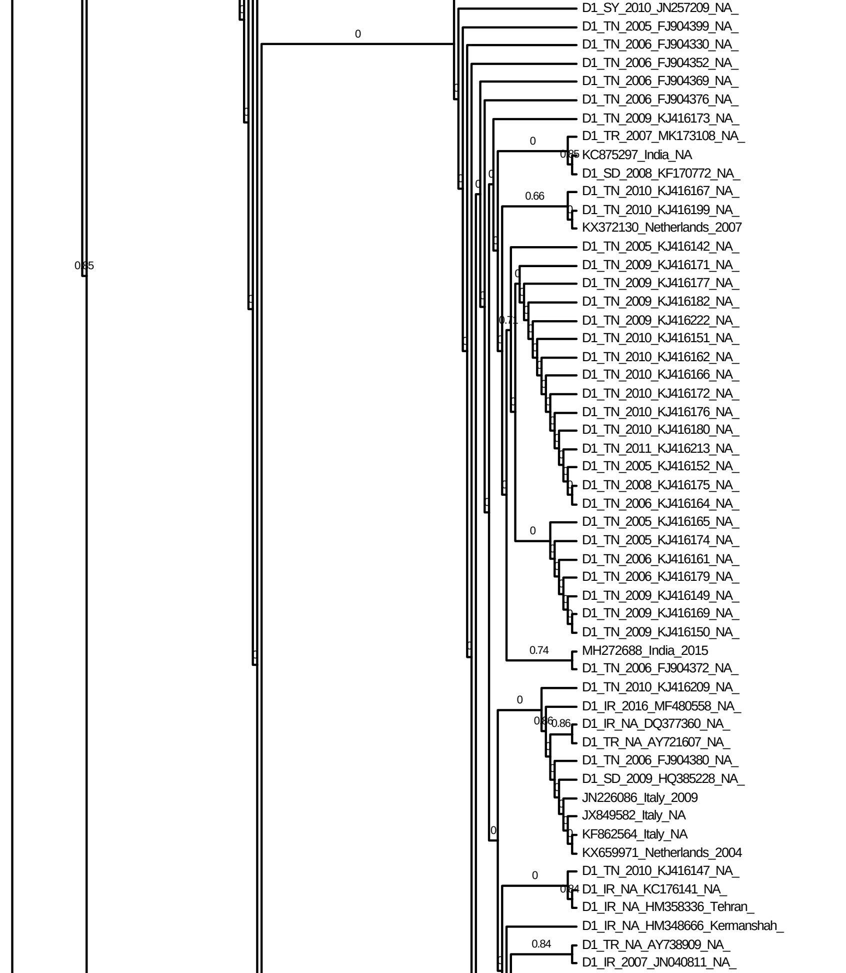
D1_SY_2010_JN257209_NA_
D1_TN_2005_FJ904399_NA_
D1_TN_2006_FJ904330_NA_
D1_TN_2006_FJ904352_NA_
D1_TN_2006_FJ904369_NA_
D1_TN_2006_FJ904376_NA_
D1_TN_2009_KJ416173_NA_
D1_TR_2007_MK173108_NA_
KC875297_India_NA
D1_SD_2008_KF170772_NA_
D1_TN_2010_KJ416167_NA_
D1_TN_2010_KJ416199_NA_
KX372130_Netherlands_2007
D1_TN_2005_KJ416142_NA_
D1_TN_2009_KJ416171_NA_
D1_TN_2009_KJ416177_NA_
D1_TN_2009_KJ416182_NA_
D1_TN_2009_KJ416222_NA_
D1_TN_2010_KJ416151_NA_
D1_TN_2010_KJ416162_NA_
D1_TN_2010_KJ416166_NA_
D1_TN_2010_KJ416172_NA_
D1_TN_2010_KJ416176_NA_
D1_TN_2010_KJ416180_NA_
D1_TN_2011_KJ416213_NA_
D1_TN_2005_KJ416152_NA_
D1_TN_2008_KJ416175_NA_
D1_TN_2006_KJ416164_NA_
D1_TN_2005_KJ416165_NA_
D1_TN_2005_KJ416174_NA_
D1_TN_2006_KJ416161_NA_
D1_TN_2006_KJ416179_NA_
D1_TN_2009_KJ416149_NA_
D1_TN_2009_KJ416169_NA_
D1_TN_2009_KJ416150_NA_
MH272688_India_2015
D1_TN_2006_FJ904372_NA_
D1_TN_2010_KJ416209_NA_
D1_IR_2016_MF480558_NA_
D1_IR_NA_DQ377360_NA_
D1_TR_NA_AY721607_NA_
D1_TN_2006_FJ904380_NA_
D1_SD_2009_HQ385228_NA_
JN226086_Italy_2009
JX849582_Italy_NA
KF862564_Italy_NA
KX659971_Netherlands_2004
D1_TN_2010_KJ416147_NA_
D1_IR_NA_KC176141_NA_
D1_IR_NA_HM358336_Tehran_
D1_IR_NA_HM348666_Kermanshah_
D1_TR_NA_AY738909_NA_
D1_IR_2007_JN040811_NA_

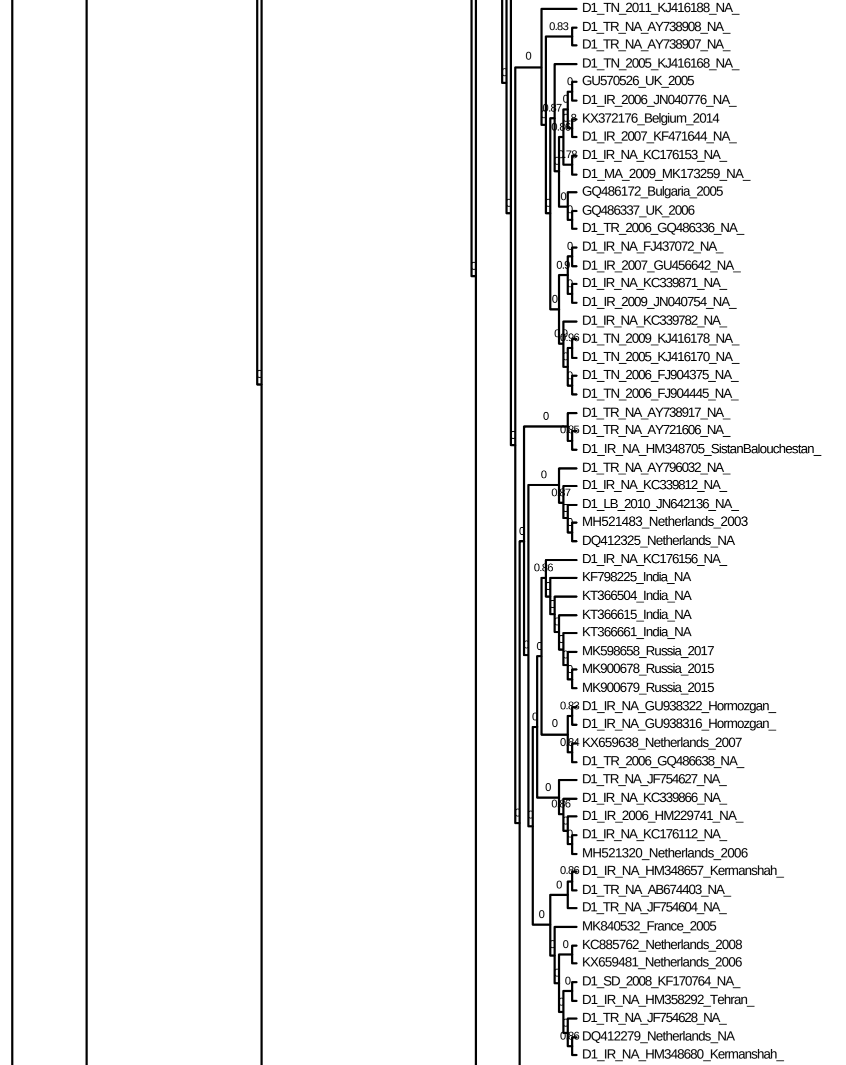
D1_TN_2011_KJ416188_NA_
D1_TR_NA_AY738908_NA_
D1_TR_NA_AY738907_NA_
D1_TN_2005_KJ416168_NA_
GU570526_UK_2005
D1_IR_2006_JN040776_NA_
KX372176_Belgium_2014
D1_IR_2007_KF471644_NA_
D1_IR_NA_KC176153_NA_
D1_MA_2009_MK173259_NA_
GQ486172_Bulgaria_2005
GQ486337_UK_2006
D1_TR_2006_GQ486336_NA_
D1_IR_NA_FJ437072_NA_
D1_IR_2007_GU456642_NA_
D1_IR_NA_KC339871_NA_
D1_IR_2009_JN040754_NA_
D1_IR_NA_KC339782_NA_
D1_TN_2009_KJ416178_NA_
D1_TN_2005_KJ416170_NA_
D1_TN_2006_FJ904375_NA_
D1_TN_2006_FJ904445_NA_
D1_TR_NA_AY738917_NA_
D1_TR_NA_AY721606_NA_
D1_IR_NA_HM348705_SistanBalouchestan_
D1_TR_NA_AY796032_NA_
D1_IR_NA_KC339812_NA_
D1_LB_2010_JN642136_NA_
MH521483_Netherlands_2003
DQ412325_Netherlands_NA
D1_IR_NA_KC176156_NA_
KF798225_India_NA
KT366504_India_NA
KT366615_India_NA
KT366661_India_NA
MK598658_Russia_2017
MK900678_Russia_2015
MK900679_Russia_2015
D1_IR_NA_GU938322_Hormozgan_
D1_IR_NA_GU938316_Hormozgan_
KX659638_Netherlands_2007
D1_TR_2006_GQ486638_NA_
D1_TR_NA_JF754627_NA_
D1_IR_NA_KC339866_NA_
D1_IR_2006_HM229741_NA_
D1_IR_NA_KC176112_NA_
MH521320_Netherlands_2006
D1_IR_NA_HM348657_Kermanshah_
D1_TR_NA_AB674403_NA_
D1_TR_NA_JF754604_NA_
MK840532_France_2005
KC885762_Netherlands_2008
KX659481_Netherlands_2006
D1_SD_2008_KF170764_NA_
D1_IR_NA_HM358292_Tehran_
D1_TR_NA_JF754628_NA_
DQ412279_Netherlands_NA
D1_IR_NA_HM348680_Kermanshah_

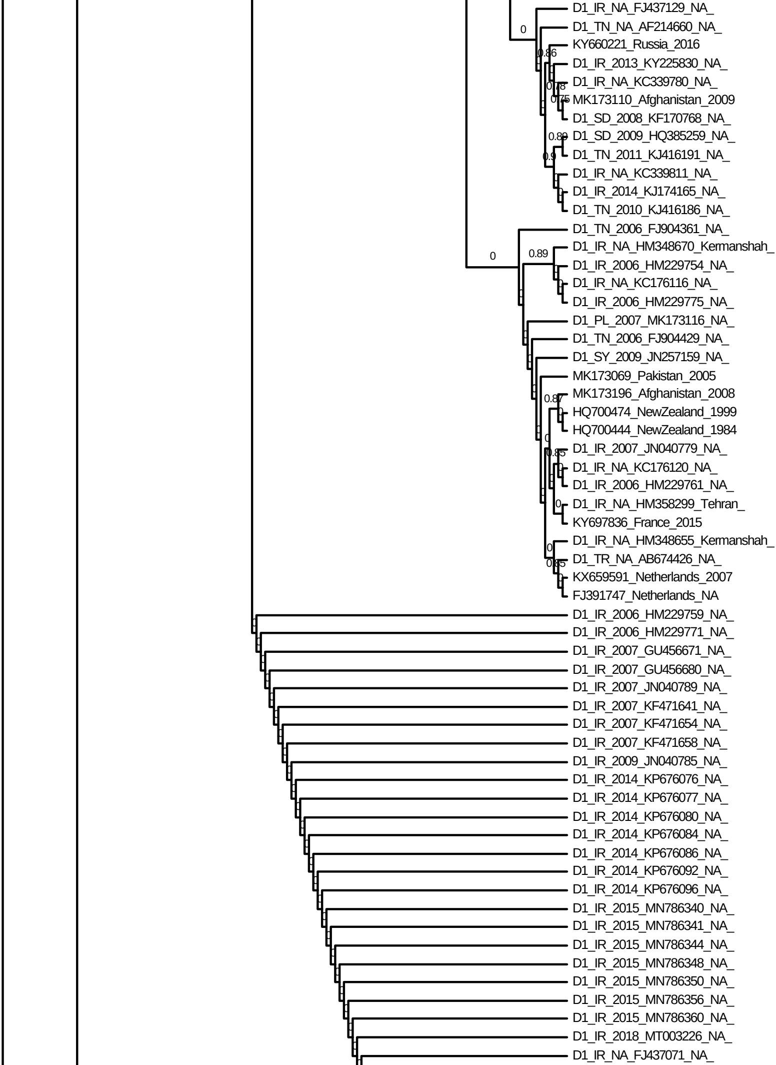
D1_IR_NA_FJ437129_NA_
D1_TN_NA_AF214660_NA_
KY660221_Russia_2016
D1_IR_2013_KY225830_NA_
D1_IR_NA_KC339780_NA_
MK173110_Afghanistan_2009
D1_SD_2008_KF170768_NA_
D1_SD_2009_HQ385259_NA_
D1_TN_2011_KJ416191_NA_
D1_IR_NA_KC339811_NA_
D1_IR_2014_KJ174165_NA_
D1_TN_2010_KJ416186_NA_
D1_TN_2006_FJ904361_NA_
D1_IR_NA_HM348670_Kermanshah_
D1_IR_2006_HM229754_NA_
D1_IR_NA_KC176116_NA_
D1_IR_2006_HM229775_NA_
D1_PL_2007_MK173116_NA_
D1_TN_2006_FJ904429_NA_
D1_SY_2009_JN257159_NA_
MK173069_Pakistan_2005
MK173196_Afghanistan_2008
HQ700474_NewZealand_1999
HQ700444_NewZealand_1984
D1_IR_2007_JN040779_NA_
D1_IR_NA_KC176120_NA_
D1_IR_2006_HM229761_NA_
D1_IR_NA_HM358299_Tehran_
KY697836_France_2015
D1_IR_NA_HM348655_Kermanshah_
D1_TR_NA_AB674426_NA_
KX659591_Netherlands_2007
FJ391747_Netherlands_NA
D1_IR_2006_HM229759_NA_
D1_IR_2006_HM229771_NA_
D1_IR_2007_GU456671_NA_
D1_IR_2007_GU456680_NA_
D1_IR_2007_JN040789_NA_
D1_IR_2007_KF471641_NA_
D1_IR_2007_KF471654_NA_
D1_IR_2007_KF471658_NA_
D1_IR_2009_JN040785_NA_
D1_IR_2014_KP676076_NA_
D1_IR_2014_KP676077_NA_
D1_IR_2014_KP676080_NA_
D1_IR_2014_KP676084_NA_
D1_IR_2014_KP676086_NA_
D1_IR_2014_KP676092_NA_
D1_IR_2014_KP676096_NA_
D1_IR_2015_MN786340_NA_
D1_IR_2015_MN786341_NA_
D1_IR_2015_MN786344_NA_
D1_IR_2015_MN786348_NA_
D1_IR_2015_MN786350_NA_
D1_IR_2015_MN786356_NA_
D1_IR_2015_MN786360_NA_
D1_IR_2018_MT003226_NA_
D1_IR_NA_FJ437071_NA_

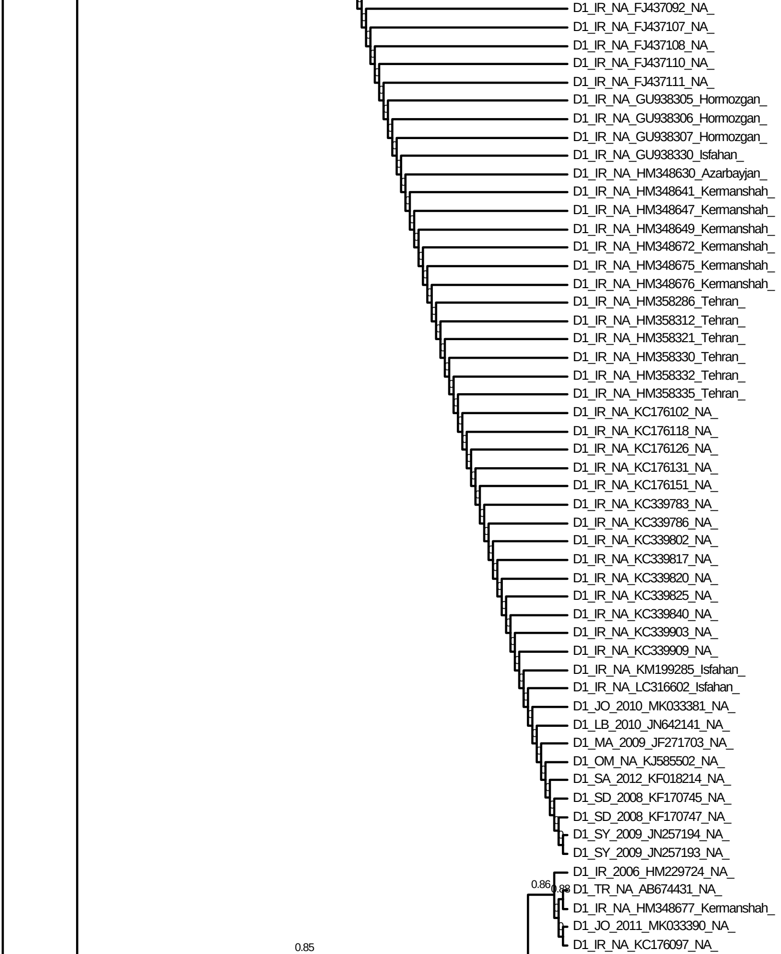

D1_IR_NA_FJ437092_NA_
D1_IR_NA_FJ437107_NA_
D1_IR_NA_FJ437108_NA_
D1_IR_NA_FJ437110_NA_
D1_IR_NA_FJ437111_NA_
D1_IR_NA_GU938305_Hormozgan_
D1_IR_NA_GU938306_Hormozgan_
D1_IR_NA_GU938307_Hormozgan_
D1_IR_NA_GU938330_Isfahan_
D1_IR_NA_HM348630_Azarbayjan_
D1_IR_NA_HM348641_Kermanshah_
D1_IR_NA_HM348647_Kermanshah_
D1_IR_NA_HM348649_Kermanshah_
D1_IR_NA_HM348672_Kermanshah_
D1_IR_NA_HM348675_Kermanshah_
D1_IR_NA_HM348676_Kermanshah_
D1_IR_NA_HM358286_Tehran_
D1_IR_NA_HM358312_Tehran_
D1_IR_NA_HM358321_Tehran_
D1_IR_NA_HM358330_Tehran_
D1_IR_NA_HM358332_Tehran_
D1_IR_NA_HM358335_Tehran_
D1_IR_NA_KC176102_NA_
D1_IR_NA_KC176118_NA_
D1_IR_NA_KC176126_NA_
D1_IR_NA_KC176131_NA_
D1_IR_NA_KC176151_NA_
D1_IR_NA_KC339783_NA_
D1_IR_NA_KC339786_NA_
D1_IR_NA_KC339802_NA_
D1_IR_NA_KC339817_NA_
D1_IR_NA_KC339820_NA_
D1_IR_NA_KC339825_NA_
D1_IR_NA_KC339840_NA_
D1_IR_NA_KC339903_NA_
D1_IR_NA_KC339909_NA_
D1_IR_NA_KM199285_Isfahan_
D1_IR_NA_LC316602_Isfahan_
D1_JO_2010_MK033381_NA_
D1_LB_2010_JN642141_NA_
D1_MA_2009_JF271703_NA_
D1_OM_NA_KJ585502_NA_
D1_SA_2012_KF018214_NA_
D1_SD_2008_KF170745_NA_
D1_SD_2008_KF170747_NA_
D1_SY_2009_JN257194_NA_
D1_SY_2009_JN257193_NA_
D1_IR_2006_HM229724_NA_
0.86
0.88
D1_TR_NA_AB674431_NA_
D1_IR_NA_HM348677_Kermanshah_
D1_JO_2011_MK033390_NA_
D1_IR_NA_KC176097_NA_
0.85

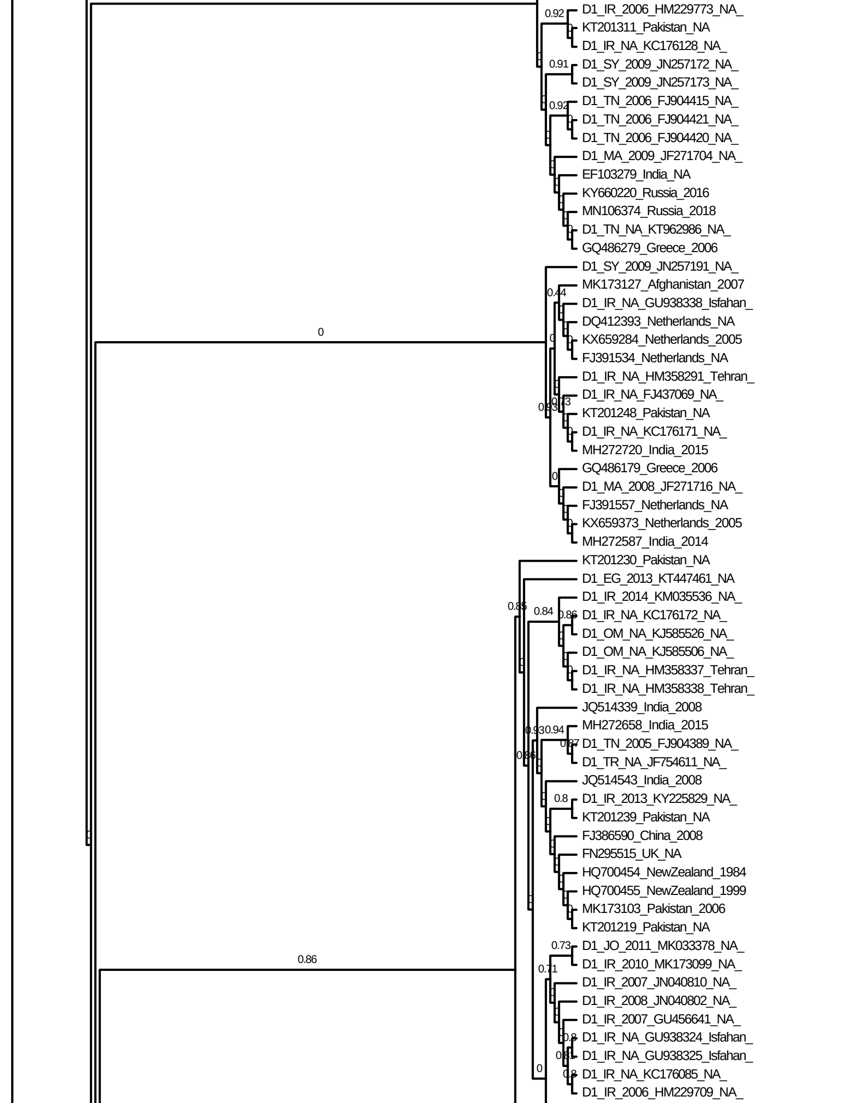

D1_IR_2006_HM229773_NA_
KT201311_Pakistan_NA
D1_IR_NA_KC176128_NA_
D1_SY_2009_JN257172_NA_
D1_SY_2009_JN257173_NA_
D1_TN_2006_FJ904415_NA_
D1_TN_2006_FJ904421_NA_
D1_TN_2006_FJ904420_NA_
D1_MA_2009_JF271704_NA_
EF103279_India_NA
KY660220_Russia_2016
MN106374_Russia_2018
D1_TN_NA_KT962986_NA_
GQ486279_Greece_2006
D1_SY_2009_JN257191_NA_
MK173127_Afghanistan_2007
D1_IR_NA_GU938338_Isfahan_
DQ412393_Netherlands_NA
KX659284_Netherlands_2005
FJ391534_Netherlands_NA
D1_IR_NA_HM358291_Tehran_
D1_IR_NA_FJ437069_NA_
KT201248_Pakistan_NA
D1_IR_NA_KC176171_NA_
MH272720_India_2015
GQ486179_Greece_2006
D1_MA_2008_JF271716_NA_
FJ391557_Netherlands_NA
KX659373_Netherlands_2005
MH272587_India_2014
KT201230_Pakistan_NA
D1_EG_2013_KT447461_NA
D1_IR_2014_KM035536_NA_
D1_IR_NA_KC176172_NA_
D1_OM_NA_KJ585526_NA_
D1_OM_NA_KJ585506_NA_
D1_IR_NA_HM358337_Tehran_
D1_IR_NA_HM358338_Tehran_
JQ514339_India_2008
MH272658_India_2015
D1_TN_2005_FJ904389_NA_
D1_TR_NA_JF754611_NA_
JQ514543_India_2008
D1_IR_2013_KY225829_NA_
KT201239_Pakistan_NA
FJ386590_China_2008
FN295515_UK_NA
HQ700454_NewZealand_1984
HQ700455_NewZealand_1999
MK173103_Pakistan_2006
KT201219_Pakistan_NA
D1_JO_2011_MK033378_NA_
D1_IR_2010_MK173099_NA_
D1_IR_2007_JN040810_NA_
D1_IR_2008_JN040802_NA_
D1_IR_2007_GU456641_NA_
D1_IR_NA_GU938324_Isfahan_
D1_IR_NA_GU938325_Isfahan_
D1_IR_NA_KC176085_NA_
D1_IR_2006_HM229709_NA_

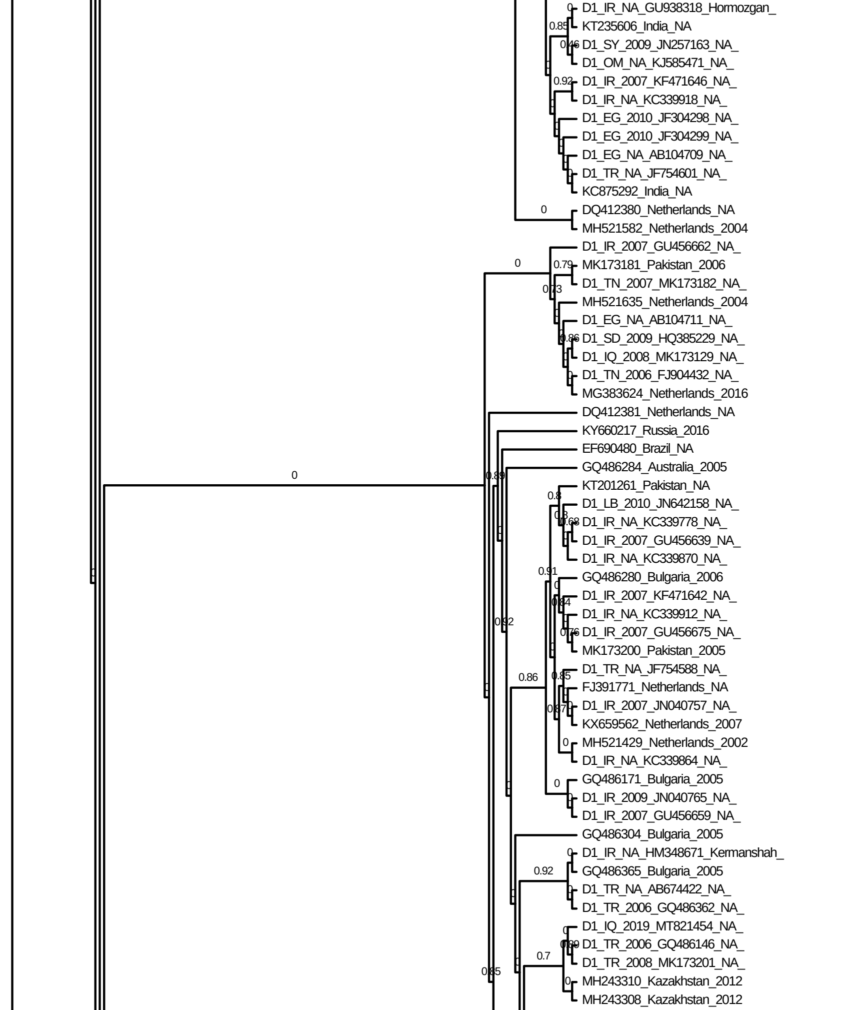

D1_IR_NA_GU938318_Hormozgan_
KT235606_India_NA
D1_SY_2009_JN257163_NA_
D1_OM_NA_KJ585471_NA_
D1_IR_2007_KF471646_NA_
D1_IR_NA_KC339918_NA_
D1_EG_2010_JF304298_NA_
D1_EG_2010_JF304299_NA_
D1_EG_NA_AB104709_NA_
D1_TR_NA_JF754601_NA_
KC875292_India_NA
DQ412380_Netherlands_NA
MH521582_Netherlands_2004
D1_IR_2007_GU456662_NA_
MK173181_Pakistan_2006
D1_TN_2007_MK173182_NA_
MH521635_Netherlands_2004
D1_EG_NA_AB104711_NA_
D1_SD_2009_HQ385229_NA_
D1_IQ_2008_MK173129_NA_
D1_TN_2006_FJ904432_NA_
MG383624_Netherlands_2016
DQ412381_Netherlands_NA
KY660217_Russia_2016
EF690480_Brazil_NA
GQ486284_Australia_2005
KT201261_Pakistan_NA
D1_LB_2010_JN642158_NA_
D1_IR_NA_KC339778_NA_
D1_IR_2007_GU456639_NA_
D1_IR_NA_KC339870_NA_
GQ486280_Bulgaria_2006
D1_IR_2007_KF471642_NA_
D1_IR_NA_KC339912_NA_
D1_IR_2007_GU456675_NA_
MK173200_Pakistan_2005
D1_TR_NA_JF754588_NA_
FJ391771_Netherlands_NA
D1_IR_2007_JN040757_NA_
KX659562_Netherlands_2007
MH521429_Netherlands_2002
D1_IR_NA_KC339864_NA_
GQ486171_Bulgaria_2005
D1_IR_2009_JN040765_NA_
D1_IR_2007_GU456659_NA_
GQ486304_Bulgaria_2005
D1_IR_NA_HM348671_Kermanshah_
GQ486365_Bulgaria_2005
D1_TR_NA_AB674422_NA_
D1_TR_2006_GQ486362_NA_
D1_IQ_2019_MT821454_NA_
D1_TR_2006_GQ486146_NA_
D1_TR_2008_MK173201_NA_
MH243310_Kazakhstan_2012
MH243308_Kazakhstan_2012

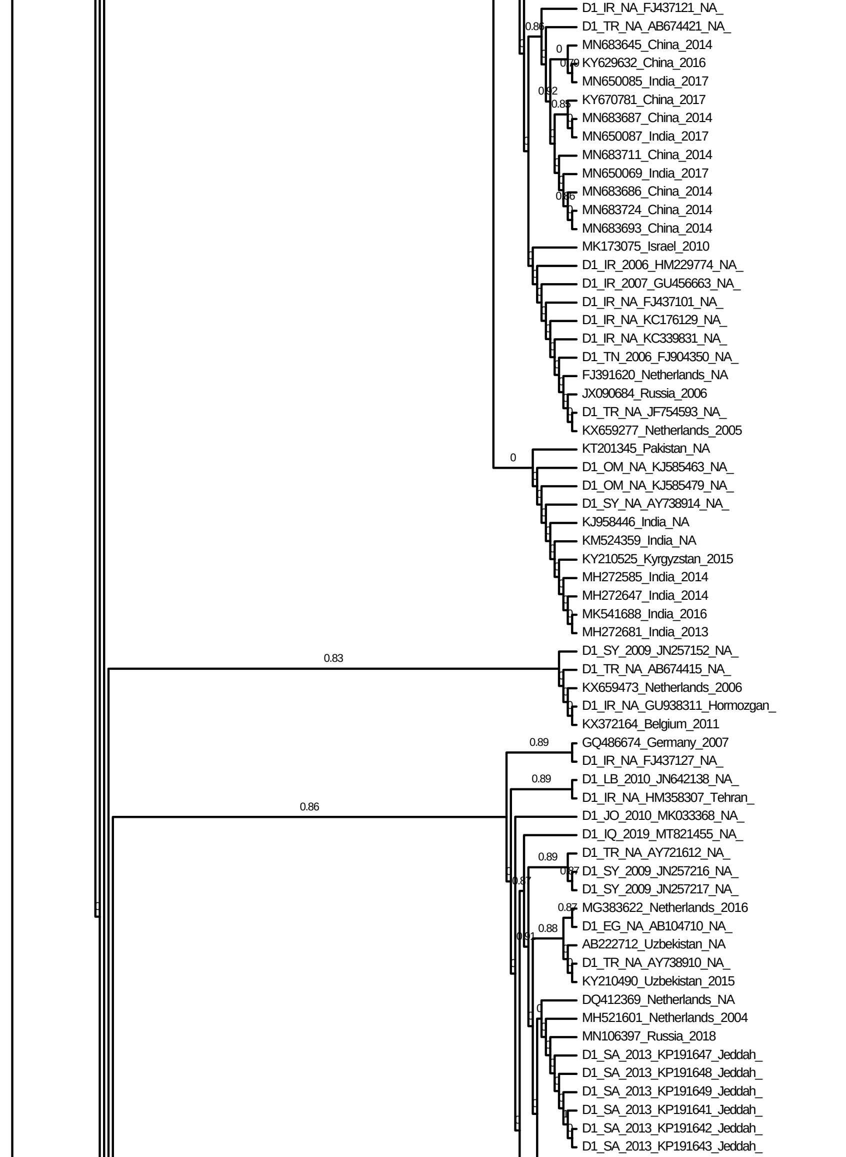
D1_IR_NA_FJ437121_NA_
D1_TR_NA_AB674421_NA_
MN683645_China_2014
KY629632_China_2016
MN650085_India_2017
KY670781_China_2017
MN683687_China_2014
MN650087_India_2017
MN683711_China_2014
MN650069_India_2017
MN683686_China_2014
MN683724_China_2014
MN683693_China_2014
MK173075_Israel_2010
D1_IR_2006_HM229774_NA_
D1_IR_2007_GU456663_NA_
D1_IR_NA_FJ437101_NA_
D1_IR_NA_KC176129_NA_
D1_IR_NA_KC339831_NA_
D1_TN_2006_FJ904350_NA_
FJ391620_Netherlands_NA
JX090684_Russia_2006
D1_TR_NA_JF754593_NA_
KX659277_Netherlands_2005
KT201345_Pakistan_NA
D1_OM_NA_KJ585463_NA_
D1_OM_NA_KJ585479_NA_
D1_SY_NA_AY738914_NA_
KJ958446_India_NA
KM524359_India_NA
KY210525_Kyrgyzstan_2015
MH272585_India_2014
MH272647_India_2014
MK541688_India_2016
MH272681_India_2013
D1_SY_2009_JN257152_NA_
D1_TR_NA_AB674415_NA_
KX659473_Netherlands_2006
D1_IR_NA_GU938311_Hormozgan_
KX372164_Belgium_2011
GQ486674_Germany_2007
D1_IR_NA_FJ437127_NA_
D1_LB_2010_JN642138_NA_
D1_IR_NA_HM358307_Tehran_
D1_JO_2010_MK033368_NA_
D1_IQ_2019_MT821455_NA_
D1_TR_NA_AY721612_NA_
D1_SY_2009_JN257216_NA_
D1_SY_2009_JN257217_NA_
MG383622_Netherlands_2016
D1_EG_NA_AB104710_NA_
AB222712_Uzbekistan_NA
D1_TR_NA_AY738910_NA_
KY210490_Uzbekistan_2015
DQ412369_Netherlands_NA
MH521601_Netherlands_2004
MN106397_Russia_2018
D1_SA_2013_KP191647_Jeddah_
D1_SA_2013_KP191648_Jeddah_
D1_SA_2013_KP191649_Jeddah_
D1_SA_2013_KP191641_Jeddah_
D1_SA_2013_KP191642_Jeddah_
D1_SA_2013_KP191643_Jeddah_

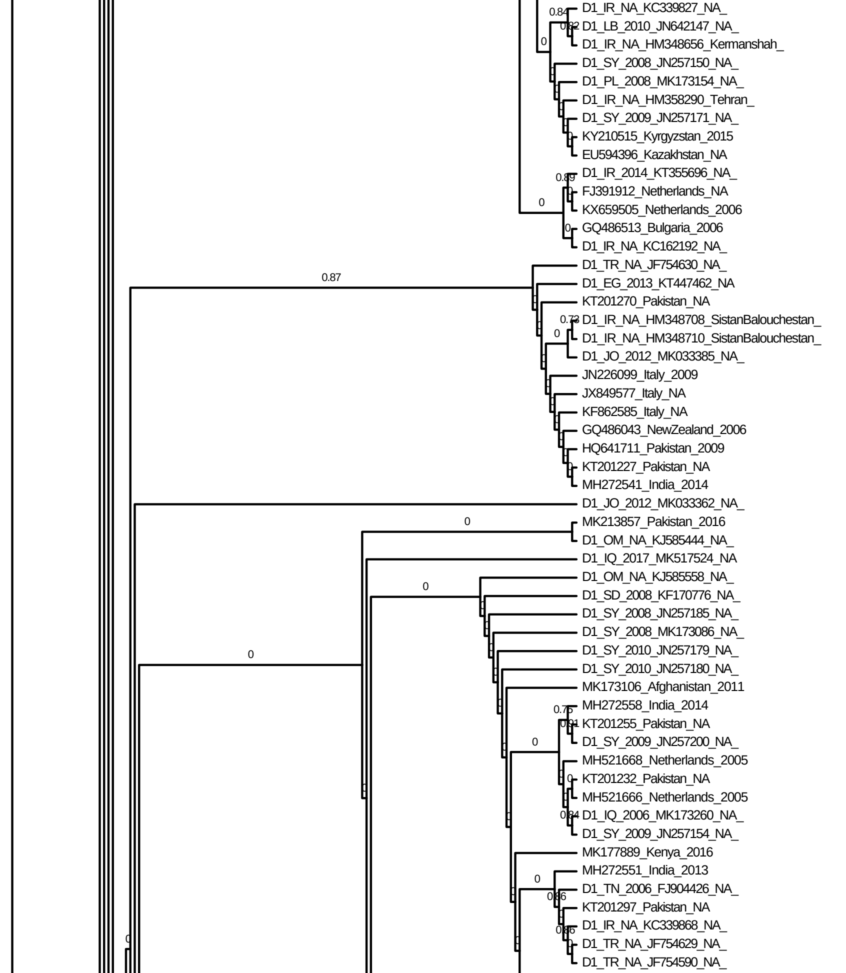
D1_IR_NA_KC339827_NA_
D1_LB_2010_JN642147_NA_
D1_IR_NA_HM348656_Kermanshah_
D1_SY_2008_JN257150_NA_
D1_PL_2008_MK173154_NA_
D1_IR_NA_HM358290_Tehran_
D1_SY_2009_JN257171_NA_
KY210515_Kyrgyzstan_2015
EU594396_Kazakhstan_NA
D1_IR_2014_KT355696_NA_
FJ391912_Netherlands_NA
KX659505_Netherlands_2006
GQ486513_Bulgaria_2006
D1_IR_NA_KC162192_NA_
D1_TR_NA_JF754630_NA_
D1_EG_2013_KT447462_NA
KT201270_Pakistan_NA
D1_IR_NA_HM348708_SistanBalouchestan_
D1_IR_NA_HM348710_SistanBalouchestan_
D1_JO_2012_MK033385_NA_
JN226099_Italy_2009
JX849577_Italy_NA
KF862585_Italy_NA
GQ486043_NewZealand_2006
HQ641711_Pakistan_2009
KT201227_Pakistan_NA
MH272541_India_2014
D1_JO_2012_MK033362_NA_
MK213857_Pakistan_2016
D1_OM_NA_KJ585444_NA_
D1_IQ_2017_MK517524_NA
D1_OM_NA_KJ585558_NA_
D1_SD_2008_KF170776_NA_
D1_SY_2008_JN257185_NA_
D1_SY_2008_MK173086_NA_
D1_SY_2010_JN257179_NA_
D1_SY_2010_JN257180_NA_
MK173106_Afghanistan_2011
MH272558_India_2014
KT201255_Pakistan_NA
D1_SY_2009_JN257200_NA_
MH521668_Netherlands_2005
KT201232_Pakistan_NA
MH521666_Netherlands_2005
D1_IQ_2006_MK173260_NA_
D1_SY_2009_JN257154_NA_
MK177889_Kenya_2016
MH272551_India_2013
D1_TN_2006_FJ904426_NA_
KT201297_Pakistan_NA
D1_IR_NA_KC339868_NA_
D1_TR_NA_JF754629_NA_
D1_TR_NA_JF754590_NA_
0.84
0.82
0.89
0.87
0.73
0.75
0.91
0.84
0.86
0

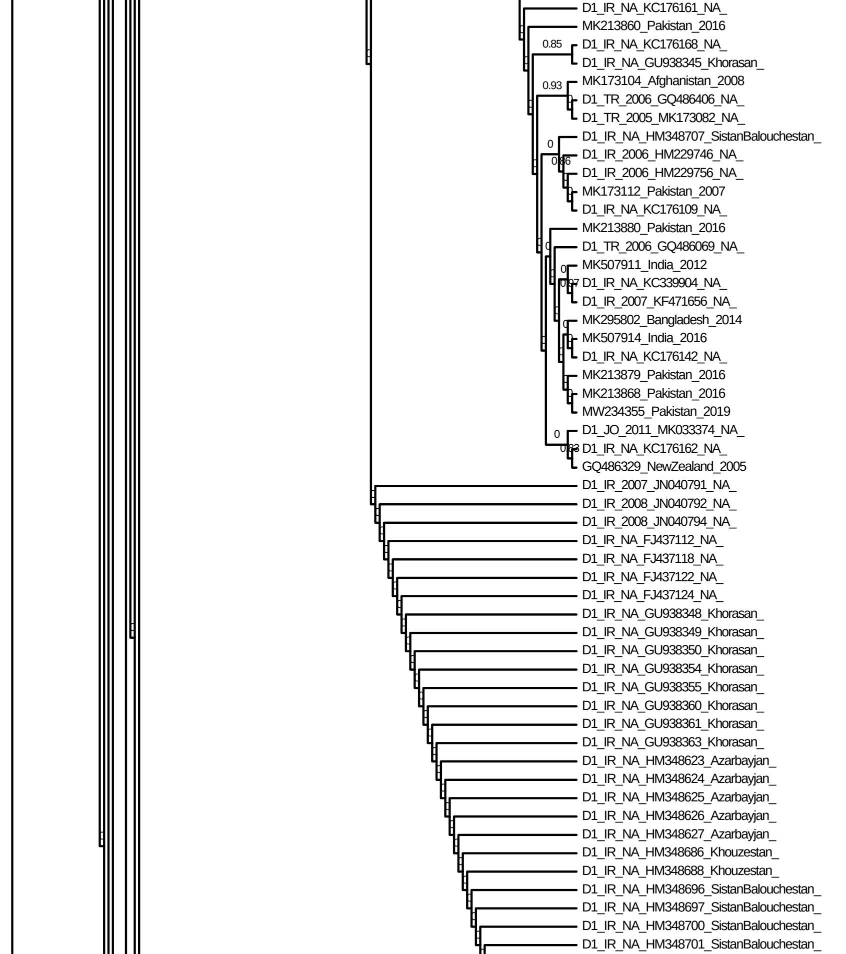
D1_IR_NA_KC176161_NA_
MK213860_Pakistan_2016
0.85
D1_IR_NA_KC176168_NA_
D1_IR_NA_GU938345_Khorasan_
0.93
MK173104_Afghanistan_2008
D1_TR_2006_GQ486406_NA_
D1_TR_2005_MK173082_NA_
D1_IR_NA_HM348707_SistanBalouchestan_
D1_IR_2006_HM229746_NA_
D1_IR_2006_HM229756_NA_
MK173112_Pakistan_2007
D1_IR_NA_KC176109_NA_
MK213880_Pakistan_2016
D1_TR_2006_GQ486069_NA_
MK507911_India_2012
D1_IR_NA_KC339904_NA_
D1_IR_2007_KF471656_NA_
MK295802_Bangladesh_2014
MK507914_India_2016
D1_IR_NA_KC176142_NA_
MK213879_Pakistan_2016
MK213868_Pakistan_2016
MW234355_Pakistan_2019
D1_JO_2011_MK033374_NA_
D1_IR_NA_KC176162_NA_
GQ486329_NewZealand_2005
D1_IR_2007_JN040791_NA_
D1_IR_2008_JN040792_NA_
D1_IR_2008_JN040794_NA_
D1_IR_NA_FJ437112_NA_
D1_IR_NA_FJ437118_NA_
D1_IR_NA_FJ437122_NA_
D1_IR_NA_FJ437124_NA_
D1_IR_NA_GU938348_Khorasan_
D1_IR_NA_GU938349_Khorasan_
D1_IR_NA_GU938350_Khorasan_
D1_IR_NA_GU938354_Khorasan_
D1_IR_NA_GU938355_Khorasan_
D1_IR_NA_GU938360_Khorasan_
D1_IR_NA_GU938361_Khorasan_
D1_IR_NA_GU938363_Khorasan_
D1_IR_NA_HM348623_Azarbayjan_
D1_IR_NA_HM348624_Azarbayjan_
D1_IR_NA_HM348625_Azarbayjan_
D1_IR_NA_HM348626_Azarbayjan_
D1_IR_NA_HM348627_Azarbayjan_
D1_IR_NA_HM348686_Khouzestan_
D1_IR_NA_HM348688_Khouzestan_
D1_IR_NA_HM348696_SistanBalouchestan_
D1_IR_NA_HM348697_SistanBalouchestan_
D1_IR_NA_HM348700_SistanBalouchestan_
D1_IR_NA_HM348701_SistanBalouchestan_

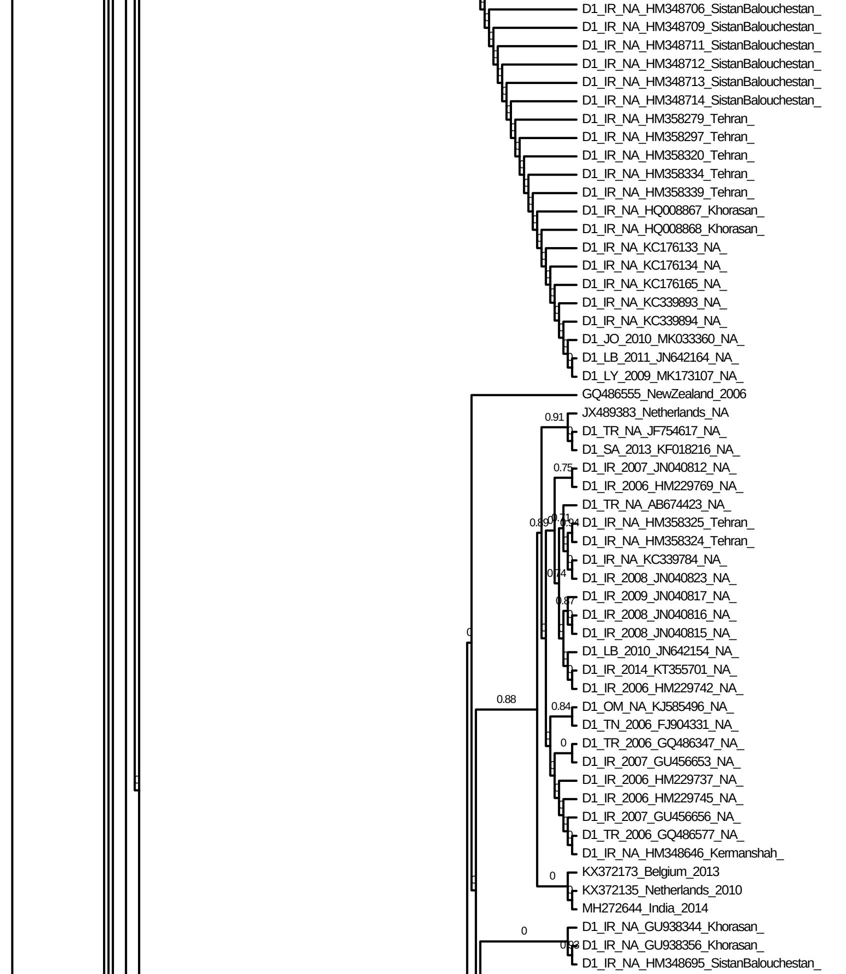
D1_IR_NA_HM348706_SistanBalouchestan_
D1_IR_NA_HM348709_SistanBalouchestan_
D1_IR_NA_HM348711_SistanBalouchestan_
D1_IR_NA_HM348712_SistanBalouchestan_
D1_IR_NA_HM348713_SistanBalouchestan_
D1_IR_NA_HM348714_SistanBalouchestan_
D1_IR_NA_HM358279_Tehran_
D1_IR_NA_HM358297_Tehran_
D1_IR_NA_HM358320_Tehran_
D1_IR_NA_HM358334_Tehran_
D1_IR_NA_HM358339_Tehran_
D1_IR_NA_HQ008867_Khorasan_
D1_IR_NA_HQ008868_Khorasan_
D1_IR_NA_KC176133_NA_
D1_IR_NA_KC176134_NA_
D1_IR_NA_KC176165_NA_
D1_IR_NA_KC339893_NA_
D1_IR_NA_KC339894_NA_
D1_JO_2010_MK033360_NA_
D1_LB_2011_JN642164_NA_
D1_LY_2009_MK173107_NA_
GQ486555_NewZealand_2006
0.91
JX489383_Netherlands_NA
D1_TR_NA_JF754617_NA_
D1_SA_2013_KF018216_NA_
0.75
D1_IR_2007_JN040812_NA_
D1_IR_2006_HM229769_NA_
D1_TR_NA_AB674423_NA_
0.89
D1_IR_NA_HM358325_Tehran_
D1_IR_NA_HM358324_Tehran_
D1_IR_NA_KC339784_NA_
D1_IR_2008_JN040823_NA_
D1_IR_2009_JN040817_NA_
D1_IR_2008_JN040816_NA_
D1_IR_2008_JN040815_NA_
D1_LB_2010_JN642154_NA_
D1_IR_2014_KT355701_NA_
D1_IR_2006_HM229742_NA_
0.88
0.84
D1_OM_NA_KJ585496_NA_
D1_TN_2006_FJ904331_NA_
0
D1_TR_2006_GQ486347_NA_
D1_IR_2007_GU456653_NA_
D1_IR_2006_HM229737_NA_
D1_IR_2006_HM229745_NA_
D1_IR_2007_GU456656_NA_
D1_TR_2006_GQ486577_NA_
D1_IR_NA_HM348646_Kermanshah_
0
KX372173_Belgium_2013
KX372135_Netherlands_2010
MH272644_India_2014
0
D1_IR_NA_GU938344_Khorasan_
D1_IR_NA_GU938356_Khorasan_
D1_IR_NA_HM348695_SistanBalouchestan_

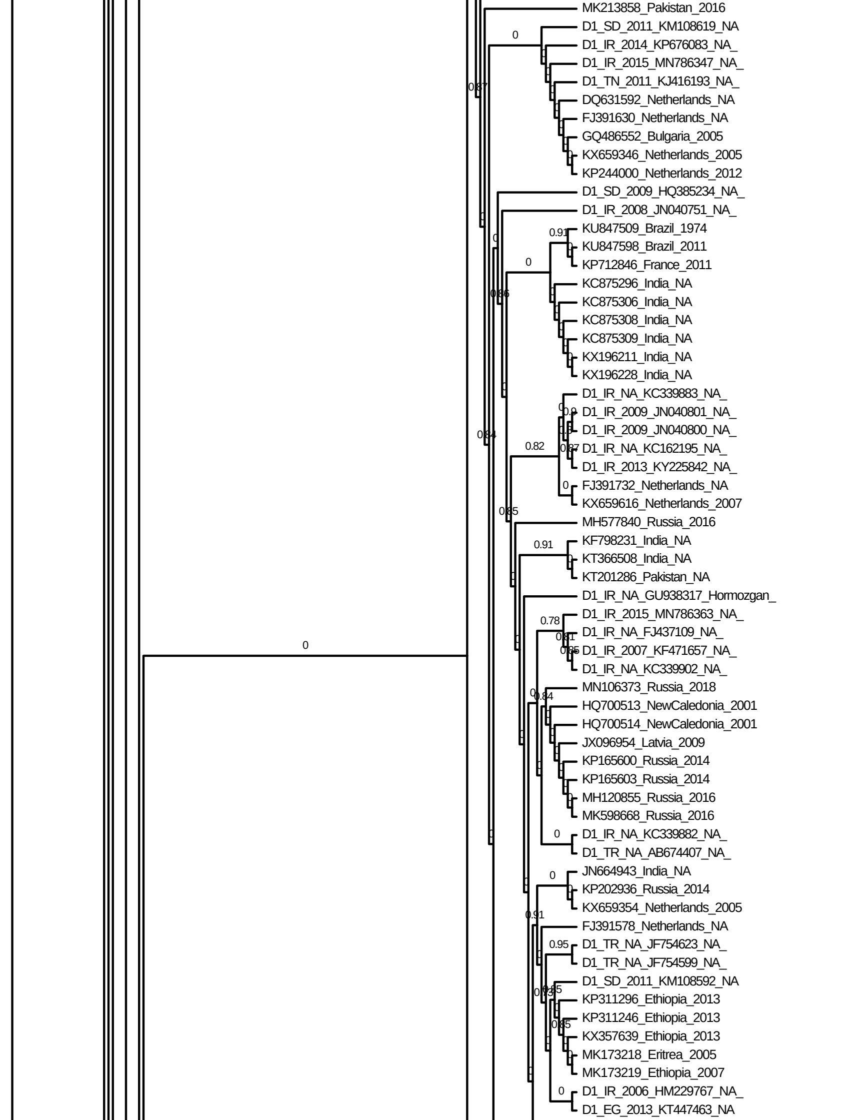
MK213858_Pakistan_2016
D1_SD_2011_KM108619_NA
D1_IR_2014_KP676083_NA_
D1_IR_2015_MN786347_NA_
D1_TN_2011_KJ416193_NA_
DQ631592_Netherlands_NA
FJ391630_Netherlands_NA
GQ486552_Bulgaria_2005
KX659346_Netherlands_2005
KP244000_Netherlands_2012
D1_SD_2009_HQ385234_NA_
D1_IR_2008_JN040751_NA_
KU847509_Brazil_1974
KU847598_Brazil_2011
KP712846_France_2011
KC875296_India_NA
KC875306_India_NA
KC875308_India_NA
KC875309_India_NA
KX196211_India_NA
KX196228_India_NA
D1_IR_NA_KC339883_NA_
D1_IR_2009_JN040801_NA_
D1_IR_2009_JN040800_NA_
D1_IR_NA_KC162195_NA_
D1_IR_2013_KY225842_NA_
FJ391732_Netherlands_NA
KX659616_Netherlands_2007
MH577840_Russia_2016
KF798231_India_NA
KT366508_India_NA
KT201286_Pakistan_NA
D1_IR_NA_GU938317_Hormozgan_
D1_IR_2015_MN786363_NA_
D1_IR_NA_FJ437109_NA_
D1_IR_2007_KF471657_NA_
D1_IR_NA_KC339902_NA_
MN106373_Russia_2018
HQ700513_NewCaledonia_2001
HQ700514_NewCaledonia_2001
JX096954_Latvia_2009
KP165600_Russia_2014
KP165603_Russia_2014
MH120855_Russia_2016
MK598668_Russia_2016
D1_IR_NA_KC339882_NA_
D1_TR_NA_AB674407_NA_
JN664943_India_NA
KP202936_Russia_2014
KX659354_Netherlands_2005
FJ391578_Netherlands_NA
D1_TR_NA_JF754623_NA_
D1_TR_NA_JF754599_NA_
D1_SD_2011_KM108592_NA
KP311296_Ethiopia_2013
KP311246_Ethiopia_2013
KX357639_Ethiopia_2013
MK173218_Eritrea_2005
MK173219_Ethiopia_2007
D1_IR_2006_HM229767_NA_
D1_EG_2013_KT447463_NA

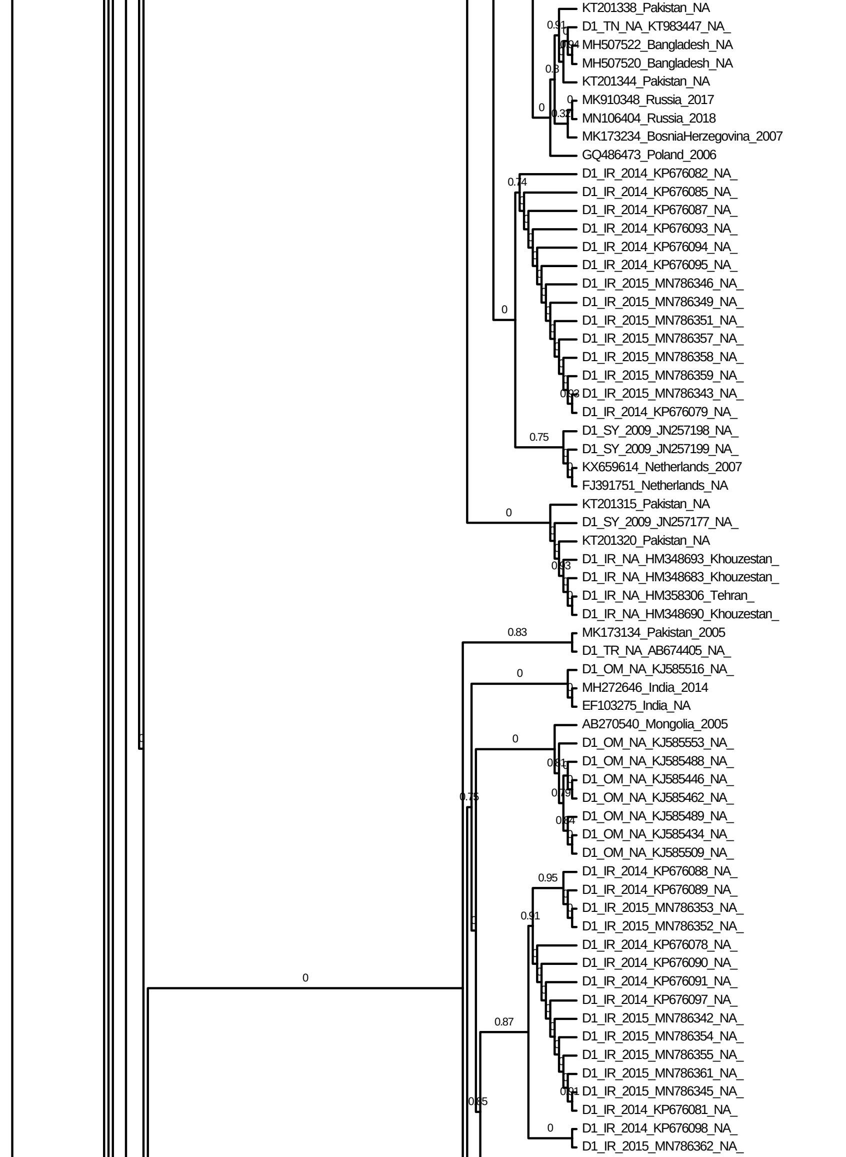
KT201338_Pakistan_NA
D1_TN_NA_KT983447_NA_
MH507522_Bangladesh_NA
MH507520_Bangladesh_NA
KT201344_Pakistan_NA
MK910348_Russia_2017
MN106404_Russia_2018
MK173234_BosniaHerzegovina_2007
GQ486473_Poland_2006
D1_IR_2014_KP676082_NA_
D1_IR_2014_KP676085_NA_
D1_IR_2014_KP676087_NA_
D1_IR_2014_KP676093_NA_
D1_IR_2014_KP676094_NA_
D1_IR_2014_KP676095_NA_
D1_IR_2015_MN786346_NA_
D1_IR_2015_MN786349_NA_
D1_IR_2015_MN786351_NA_
D1_IR_2015_MN786357_NA_
D1_IR_2015_MN786358_NA_
D1_IR_2015_MN786359_NA_
D1_IR_2015_MN786343_NA_
D1_IR_2014_KP676079_NA_
D1_SY_2009_JN257198_NA_
D1_SY_2009_JN257199_NA_
KX659614_Netherlands_2007
FJ391751_Netherlands_NA
KT201315_Pakistan_NA
D1_SY_2009_JN257177_NA_
KT201320_Pakistan_NA
D1_IR_NA_HM348693_Khouzestan_
D1_IR_NA_HM348683_Khouzestan_
D1_IR_NA_HM358306_Tehran_
D1_IR_NA_HM348690_Khouzestan_
MK173134_Pakistan_2005
D1_TR_NA_AB674405_NA_
D1_OM_NA_KJ585516_NA_
MH272646_India_2014
EF103275_India_NA
AB270540_Mongolia_2005
D1_OM_NA_KJ585553_NA_
D1_OM_NA_KJ585488_NA_
D1_OM_NA_KJ585446_NA_
D1_OM_NA_KJ585462_NA_
D1_OM_NA_KJ585489_NA_
D1_OM_NA_KJ585434_NA_
D1_OM_NA_KJ585509_NA_
D1_IR_2014_KP676088_NA_
D1_IR_2014_KP676089_NA_
D1_IR_2015_MN786353_NA_
D1_IR_2015_MN786352_NA_
D1_IR_2014_KP676078_NA_
D1_IR_2014_KP676090_NA_
D1_IR_2014_KP676091_NA_
D1_IR_2014_KP676097_NA_
D1_IR_2015_MN786342_NA_
D1_IR_2015_MN786354_NA_
D1_IR_2015_MN786355_NA_
D1_IR_2015_MN786361_NA_
D1_IR_2015_MN786345_NA_
D1_IR_2014_KP676081_NA_
D1_IR_2014_KP676098_NA_
D1_IR_2015_MN786362_NA_

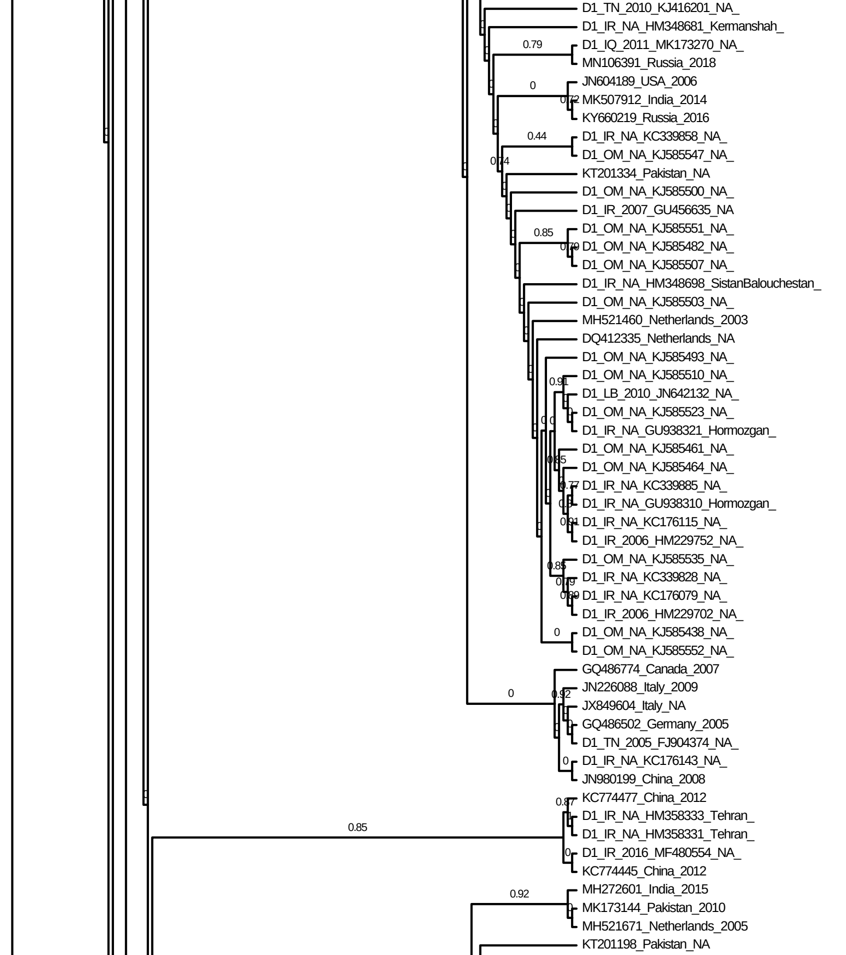
D1_TN_2010_KJ416201_NA_
D1_IR_NA_HM348681_Kermanshah_
D1_IQ_2011_MK173270_NA_
MN106391_Russia_2018
JN604189_USA_2006
MK507912_India_2014
KY660219_Russia_2016
D1_IR_NA_KC339858_NA_
D1_OM_NA_KJ585547_NA_
KT201334_Pakistan_NA
D1_OM_NA_KJ585500_NA_
D1_IR_2007_GU456635_NA
D1_OM_NA_KJ585551_NA_
D1_OM_NA_KJ585482_NA_
D1_OM_NA_KJ585507_NA_
D1_IR_NA_HM348698_SistanBalouchestan_
D1_OM_NA_KJ585503_NA_
MH521460_Netherlands_2003
DQ412335_Netherlands_NA
D1_OM_NA_KJ585493_NA_
D1_OM_NA_KJ585510_NA_
D1_LB_2010_JN642132_NA_
D1_OM_NA_KJ585523_NA_
D1_IR_NA_GU938321_Hormozgan_
D1_OM_NA_KJ585461_NA_
D1_OM_NA_KJ585464_NA_
D1_IR_NA_KC339885_NA_
D1_IR_NA_GU938310_Hormozgan_
D1_IR_NA_KC176115_NA_
D1_IR_2006_HM229752_NA_
D1_OM_NA_KJ585535_NA_
D1_IR_NA_KC339828_NA_
D1_IR_NA_KC176079_NA_
D1_IR_2006_HM229702_NA_
D1_OM_NA_KJ585438_NA_
D1_OM_NA_KJ585552_NA_
GQ486774_Canada_2007
JN226088_Italy_2009
JX849604_Italy_NA
GQ486502_Germany_2005
D1_TN_2005_FJ904374_NA_
D1_IR_NA_KC176143_NA_
JN980199_China_2008
KC774477_China_2012
D1_IR_NA_HM358333_Tehran_
D1_IR_NA_HM358331_Tehran_
D1_IR_2016_MF480554_NA_
KC774445_China_2012
MH272601_India_2015
MK173144_Pakistan_2010
MH521671_Netherlands_2005
KT201198_Pakistan_NA
0.79
0
0.44
0.85
0.85
0.92
0.92

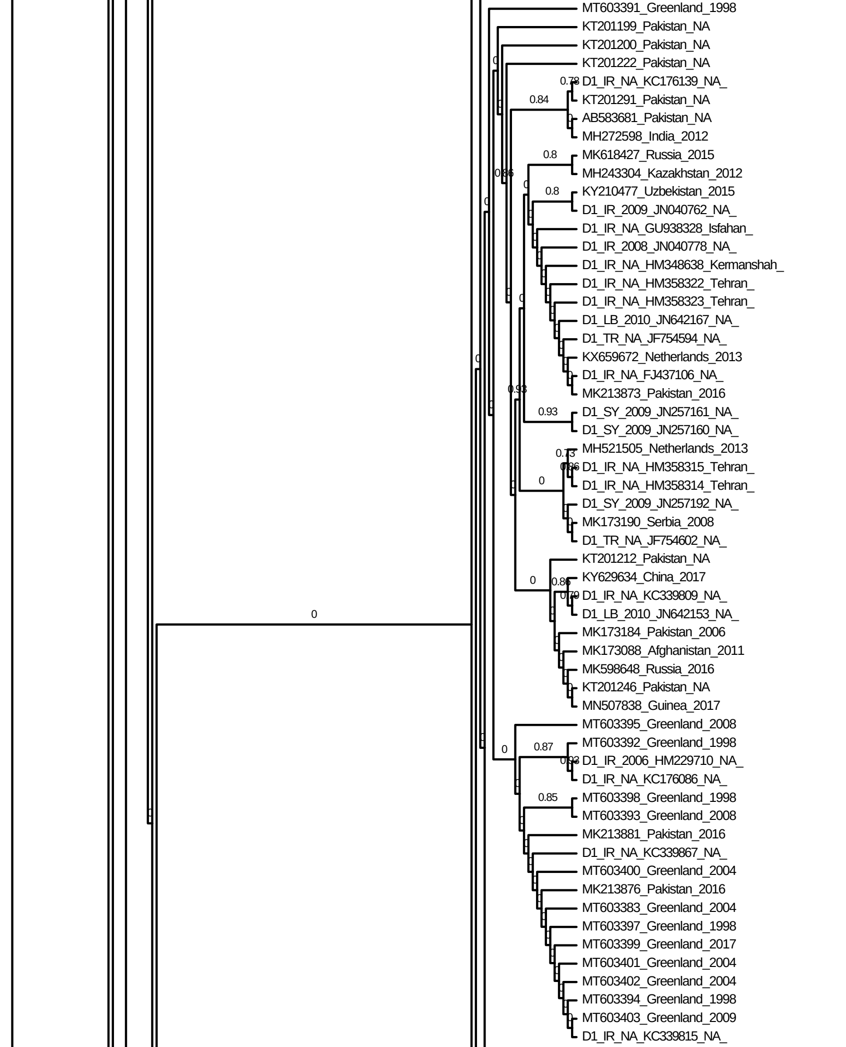
MT603391_Greenland_1998
KT201199_Pakistan_NA
KT201200_Pakistan_NA
KT201222_Pakistan_NA
D1_IR_NA_KC176139_NA_
KT201291_Pakistan_NA
AB583681_Pakistan_NA
MH272598_India_2012
MK618427_Russia_2015
MH243304_Kazakhstan_2012
KY210477_Uzbekistan_2015
D1_IR_2009_JN040762_NA_
D1_IR_NA_GU938328_Isfahan_
D1_IR_2008_JN040778_NA_
D1_IR_NA_HM348638_Kermanshah_
D1_IR_NA_HM358322_Tehran_
D1_IR_NA_HM358323_Tehran_
D1_LB_2010_JN642167_NA_
D1_TR_NA_JF754594_NA_
KX659672_Netherlands_2013
D1_IR_NA_FJ437106_NA_
MK213873_Pakistan_2016
D1_SY_2009_JN257161_NA_
D1_SY_2009_JN257160_NA_
MH521505_Netherlands_2013
D1_IR_NA_HM358315_Tehran_
D1_IR_NA_HM358314_Tehran_
D1_SY_2009_JN257192_NA_
MK173190_Serbia_2008
D1_TR_NA_JF754602_NA_
KT201212_Pakistan_NA
KY629634_China_2017
D1_IR_NA_KC339809_NA_
D1_LB_2010_JN642153_NA_
MK173184_Pakistan_2006
MK173088_Afghanistan_2011
MK598648_Russia_2016
KT201246_Pakistan_NA
MN507838_Guinea_2017
MT603395_Greenland_2008
MT603392_Greenland_1998
D1_IR_2006_HM229710_NA_
D1_IR_NA_KC176086_NA_
MT603398_Greenland_1998
MT603393_Greenland_2008
MK213881_Pakistan_2016
D1_IR_NA_KC339867_NA_
MT603400_Greenland_2004
MK213876_Pakistan_2016
MT603383_Greenland_2004
MT603397_Greenland_1998
MT603399_Greenland_2017
MT603401_Greenland_2004
MT603402_Greenland_2004
MT603394_Greenland_1998
MT603403_Greenland_2009
D1_IR_NA_KC339815_NA_

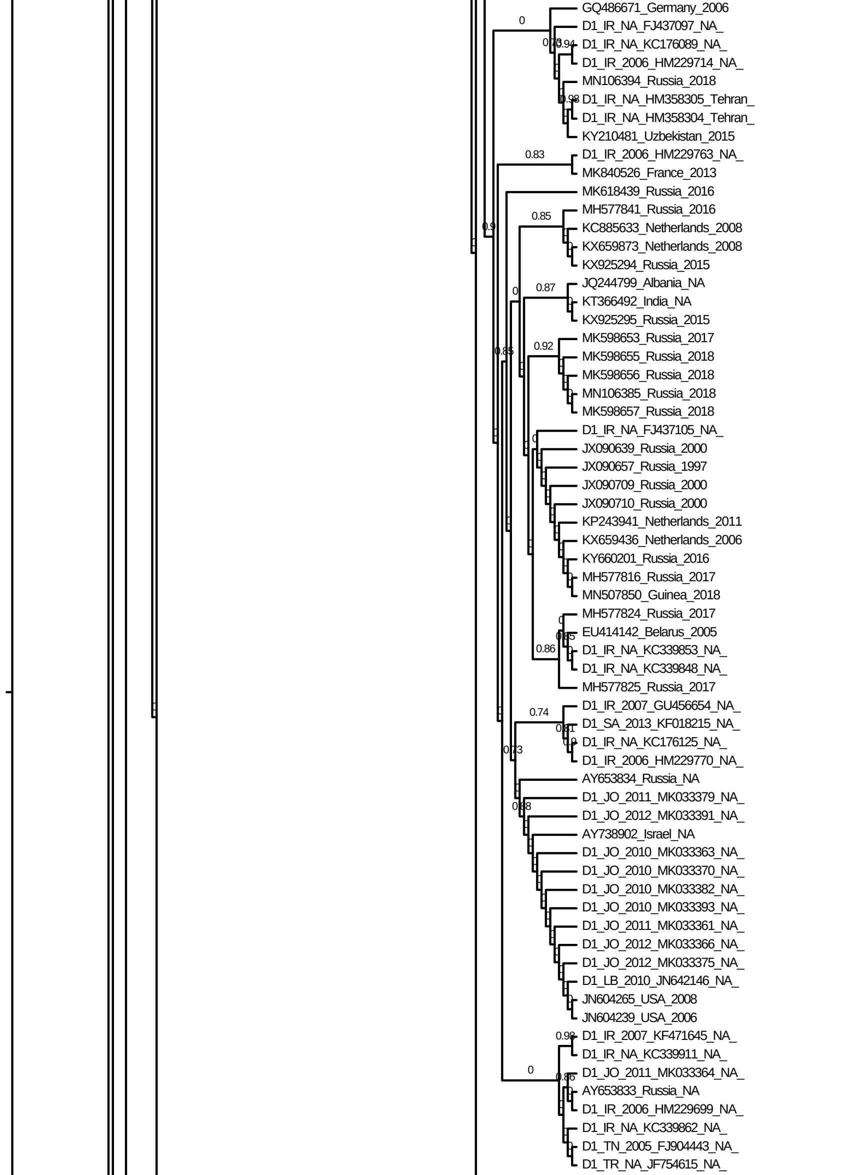
GQ486671_Germany_2006
D1_IR_NA_FJ437097_NA_
D1_IR_NA_KC176089_NA_
D1_IR_2006_HM229714_NA_
MN106394_Russia_2018
D1_IR_NA_HM358305_Tehran_
D1_IR_NA_HM358304_Tehran_
KY210481_Uzbekistan_2015
D1_IR_2006_HM229763_NA_
MK840526_France_2013
MK618439_Russia_2016
MH577841_Russia_2016
KC885633_Netherlands_2008
KX659873_Netherlands_2008
KX925294_Russia_2015
JQ244799_Albania_NA
KT366492_India_NA
KX925295_Russia_2015
MK598653_Russia_2017
MK598655_Russia_2018
MK598656_Russia_2018
MN106385_Russia_2018
MK598657_Russia_2018
D1_IR_NA_FJ437105_NA_
JX090639_Russia_2000
JX090657_Russia_1997
JX090709_Russia_2000
JX090710_Russia_2000
KP243941_Netherlands_2011
KX659436_Netherlands_2006
KY660201_Russia_2016
MH577816_Russia_2017
MN507850_Guinea_2018
MH577824_Russia_2017
EU414142_Belarus_2005
D1_IR_NA_KC339853_NA_
D1_IR_NA_KC339848_NA_
MH577825_Russia_2017
D1_IR_2007_GU456654_NA_
D1_SA_2013_KF018215_NA_
D1_IR_NA_KC176125_NA_
D1_IR_2006_HM229770_NA_
AY653834_Russia_NA
D1_JO_2011_MK033379_NA_
D1_JO_2012_MK033391_NA_
AY738902_Israel_NA
D1_JO_2010_MK033363_NA_
D1_JO_2010_MK033370_NA_
D1_JO_2010_MK033382_NA_
D1_JO_2010_MK033393_NA_
D1_JO_2011_MK033361_NA_
D1_JO_2012_MK033366_NA_
D1_JO_2012_MK033375_NA_
D1_LB_2010_JN642146_NA_
JN604265_USA_2008
JN604239_USA_2006
D1_IR_2007_KF471645_NA_
D1_IR_NA_KC339911_NA_
D1_JO_2011_MK033364_NA_
AY653833_Russia_NA
D1_IR_2006_HM229699_NA_
D1_IR_NA_KC339862_NA_
D1_TN_2005_FJ904443_NA_
D1_TR_NA_JF754615_NA_
0
0.94
0.98
0.83
0.85
0.9
0.87
0.92
0.86
0.74
0.81
0.9
0.73
0.88
0.98
0.86
0

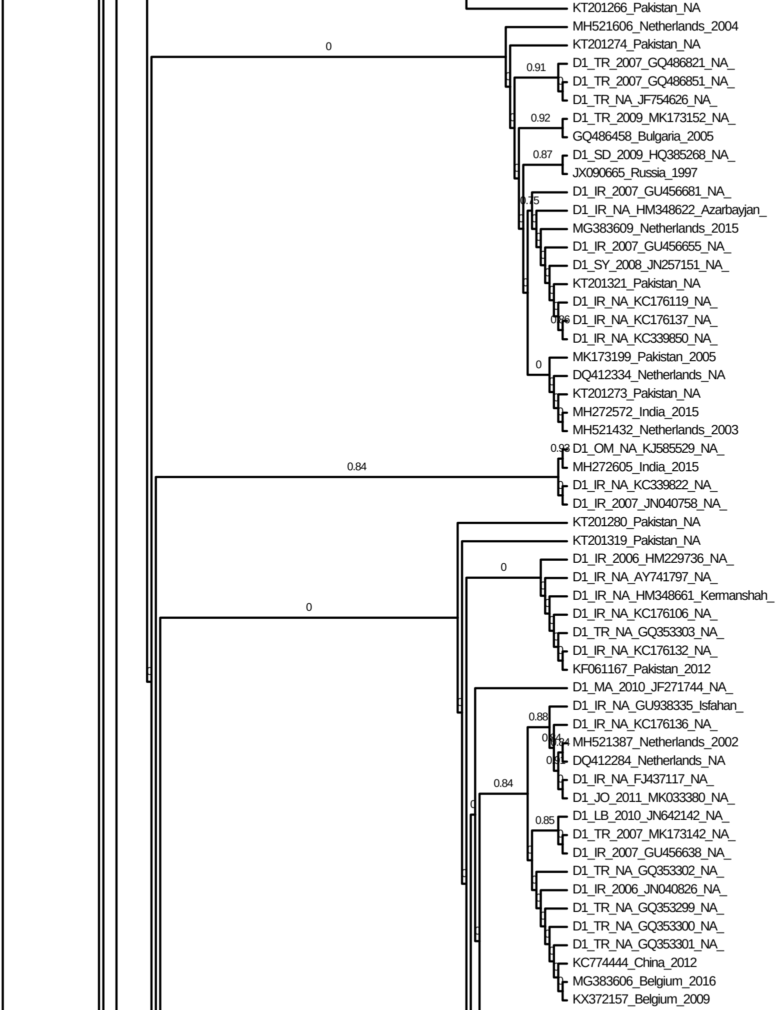
KT201266_Pakistan_NA
0
MH521606_Netherlands_2004
KT201274_Pakistan_NA
0.91
D1_TR_2007_GQ486821_NA_
D1_TR_2007_GQ486851_NA_
D1_TR_NA_JF754626_NA_
0.92
D1_TR_2009_MK173152_NA_
GQ486458_Bulgaria_2005
0.87
D1_SD_2009_HQ385268_NA_
JX090665_Russia_1997
D1_IR_2007_GU456681_NA_
0.75
D1_IR_NA_HM348622_Azarbayjan_
MG383609_Netherlands_2015
D1_IR_2007_GU456655_NA_
D1_SY_2008_JN257151_NA_
KT201321_Pakistan_NA
D1_IR_NA_KC176119_NA_
0.86
D1_IR_NA_KC176137_NA_
D1_IR_NA_KC339850_NA_
0
MK173199_Pakistan_2005
DQ412334_Netherlands_NA
KT201273_Pakistan_NA
MH272572_India_2015
MH521432_Netherlands_2003
0.84
0.93
D1_OM_NA_KJ585529_NA_
MH272605_India_2015
D1_IR_NA_KC339822_NA_
D1_IR_2007_JN040758_NA_
KT201280_Pakistan_NA
KT201319_Pakistan_NA
0
D1_IR_2006_HM229736_NA_
D1_IR_NA_AY741797_NA_
D1_IR_NA_HM348661_Kermanshah_
D1_IR_NA_KC176106_NA_
D1_TR_NA_GQ353303_NA_
D1_IR_NA_KC176132_NA_
KF061167_Pakistan_2012
D1_MA_2010_JF271744_NA_
0.88
D1_IR_NA_GU938335_Isfahan_
D1_IR_NA_KC176136_NA_
0.84
MH521387_Netherlands_2002
0.91
DQ412284_Netherlands_NA
D1_IR_NA_FJ437117_NA_
0.84
D1_JO_2011_MK033380_NA_
0.85
D1_LB_2010_JN642142_NA_
D1_TR_2007_MK173142_NA_
D1_IR_2007_GU456638_NA_
D1_TR_NA_GQ353302_NA_
D1_IR_2006_JN040826_NA_
D1_TR_NA_GQ353299_NA_
D1_TR_NA_GQ353300_NA_
D1_TR_NA_GQ353301_NA_
KC774444_China_2012
MG383606_Belgium_2016
KX372157_Belgium_2009

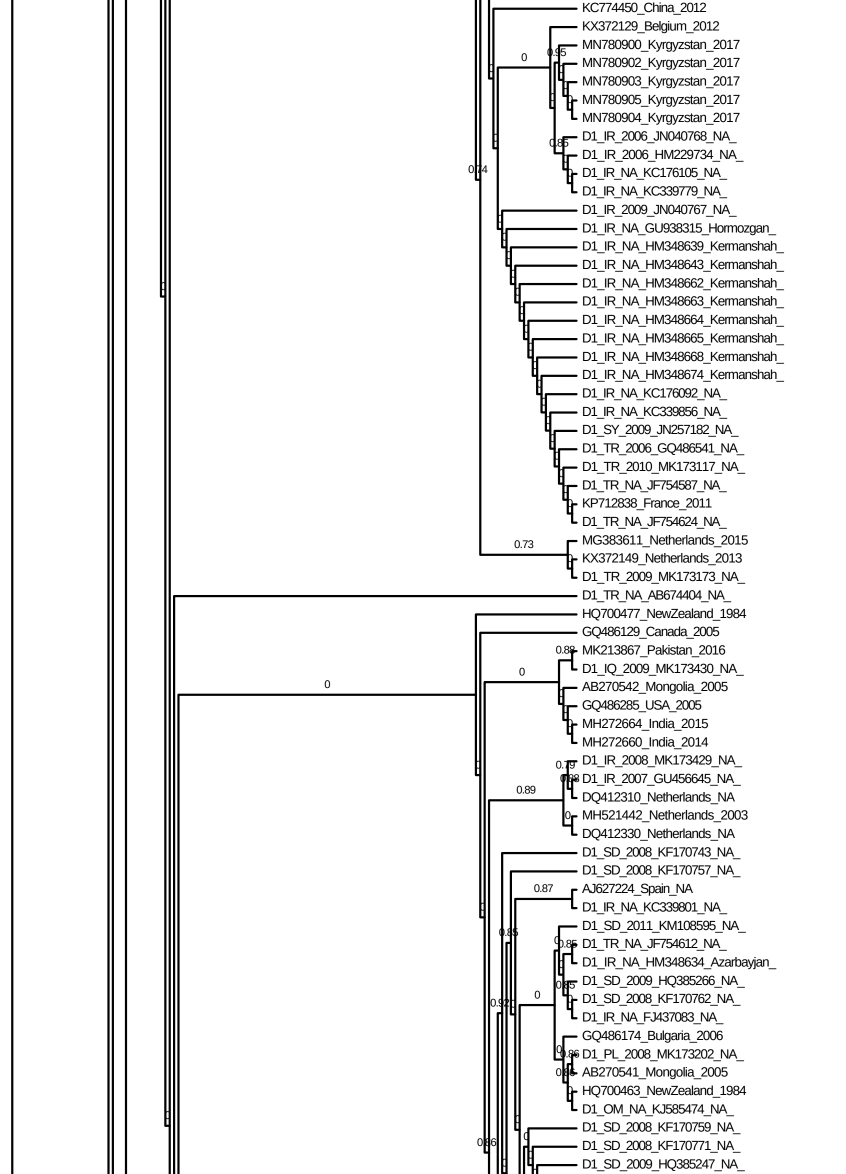

KC774450_China_2012
KX372129_Belgium_2012
MN780900_Kyrgyzstan_2017
MN780902_Kyrgyzstan_2017
MN780903_Kyrgyzstan_2017
MN780905_Kyrgyzstan_2017
MN780904_Kyrgyzstan_2017
D1_IR_2006_JN040768_NA_
D1_IR_2006_HM229734_NA_
D1_IR_NA_KC176105_NA_
D1_IR_NA_KC339779_NA_
D1_IR_2009_JN040767_NA_
D1_IR_NA_GU938315_Hormozgan_
D1_IR_NA_HM348639_Kermanshah_
D1_IR_NA_HM348643_Kermanshah_
D1_IR_NA_HM348662_Kermanshah_
D1_IR_NA_HM348663_Kermanshah_
D1_IR_NA_HM348664_Kermanshah_
D1_IR_NA_HM348665_Kermanshah_
D1_IR_NA_HM348668_Kermanshah_
D1_IR_NA_HM348674_Kermanshah_
D1_IR_NA_KC176092_NA_
D1_IR_NA_KC339856_NA_
D1_SY_2009_JN257182_NA_
D1_TR_2006_GQ486541_NA_
D1_TR_2010_MK173117_NA_
D1_TR_NA_JF754587_NA_
KP712838_France_2011
D1_TR_NA_JF754624_NA_
MG383611_Netherlands_2015
KX372149_Netherlands_2013
D1_TR_2009_MK173173_NA_
D1_TR_NA_AB674404_NA_
HQ700477_NewZealand_1984
GQ486129_Canada_2005
MK213867_Pakistan_2016
D1_IQ_2009_MK173430_NA_
AB270542_Mongolia_2005
GQ486285_USA_2005
MH272664_India_2015
MH272660_India_2014
D1_IR_2008_MK173429_NA_
D1_IR_2007_GU456645_NA_
DQ412310_Netherlands_NA
MH521442_Netherlands_2003
DQ412330_Netherlands_NA
D1_SD_2008_KF170743_NA_
D1_SD_2008_KF170757_NA_
AJ627224_Spain_NA
D1_IR_NA_KC339801_NA_
D1_SD_2011_KM108595_NA_
D1_TR_NA_JF754612_NA_
D1_IR_NA_HM348634_Azarbayjan_
D1_SD_2009_HQ385266_NA_
D1_SD_2008_KF170762_NA_
D1_IR_NA_FJ437083_NA_
GQ486174_Bulgaria_2006
D1_PL_2008_MK173202_NA_
AB270541_Mongolia_2005
HQ700463_NewZealand_1984
D1_OM_NA_KJ585474_NA_
D1_SD_2008_KF170759_NA_
D1_SD_2008_KF170771_NA_
D1_SD_2009_HQ385247_NA_

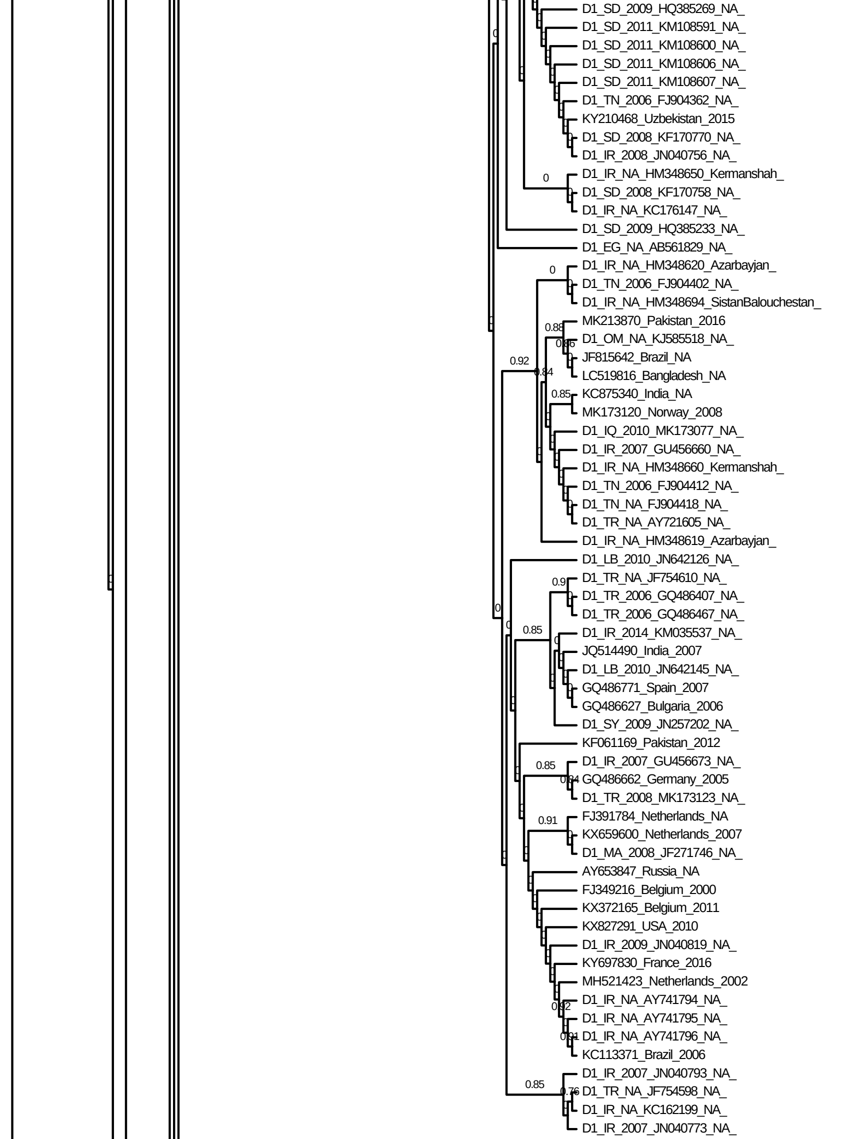
D1_SD_2009_HQ385269_NA_
D1_SD_2011_KM108591_NA_
D1_SD_2011_KM108600_NA_
D1_SD_2011_KM108606_NA_
D1_SD_2011_KM108607_NA_
D1_TN_2006_FJ904362_NA_
KY210468_Uzbekistan_2015
D1_SD_2008_KF170770_NA_
D1_IR_2008_JN040756_NA_
D1_IR_NA_HM348650_Kermanshah_
D1_SD_2008_KF170758_NA_
D1_IR_NA_KC176147_NA_
D1_SD_2009_HQ385233_NA_
D1_EG_NA_AB561829_NA_
D1_IR_NA_HM348620_Azarbayjan_
D1_TN_2006_FJ904402_NA_
D1_IR_NA_HM348694_SistanBalouchestan_
MK213870_Pakistan_2016
D1_OM_NA_KJ585518_NA_
JF815642_Brazil_NA
LC519816_Bangladesh_NA
KC875340_India_NA
MK173120_Norway_2008
D1_IQ_2010_MK173077_NA_
D1_IR_2007_GU456660_NA_
D1_IR_NA_HM348660_Kermanshah_
D1_TN_2006_FJ904412_NA_
D1_TN_NA_FJ904418_NA_
D1_TR_NA_AY721605_NA_
D1_IR_NA_HM348619_Azarbayjan_
D1_LB_2010_JN642126_NA_
D1_TR_NA_JF754610_NA_
D1_TR_2006_GQ486407_NA_
D1_TR_2006_GQ486467_NA_
D1_IR_2014_KM035537_NA_
JQ514490_India_2007
D1_LB_2010_JN642145_NA_
GQ486771_Spain_2007
GQ486627_Bulgaria_2006
D1_SY_2009_JN257202_NA_
KF061169_Pakistan_2012
D1_IR_2007_GU456673_NA_
GQ486662_Germany_2005
D1_TR_2008_MK173123_NA_
FJ391784_Netherlands_NA
KX659600_Netherlands_2007
D1_MA_2008_JF271746_NA_
AY653847_Russia_NA
FJ349216_Belgium_2000
KX372165_Belgium_2011
KX827291_USA_2010
D1_IR_2009_JN040819_NA_
KY697830_France_2016
MH521423_Netherlands_2002
D1_IR_NA_AY741794_NA_
D1_IR_NA_AY741795_NA_
D1_IR_NA_AY741796_NA_
KC113371_Brazil_2006
D1_IR_2007_JN040793_NA_
D1_TR_NA_JF754598_NA_
D1_IR_NA_KC162199_NA_
D1_IR_2007_JN040773_NA_

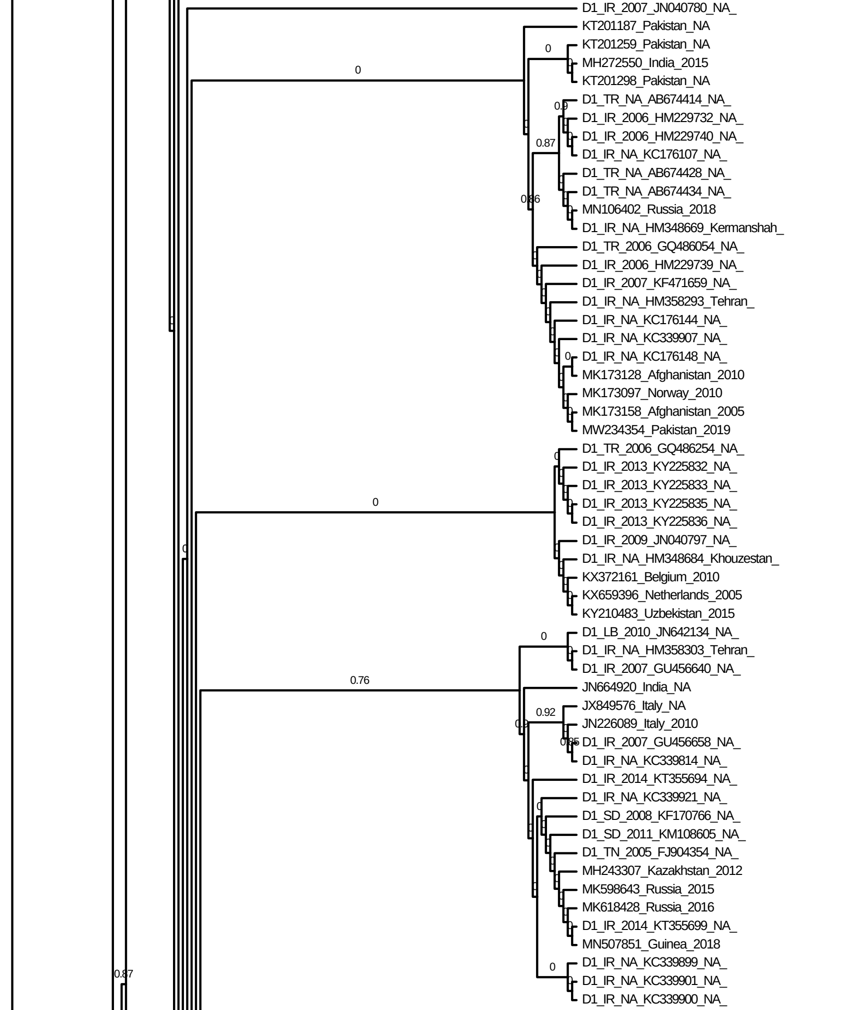
D1_IR_2007_JN040780_NA_
KT201187_Pakistan_NA
KT201259_Pakistan_NA
MH272550_India_2015
KT201298_Pakistan_NA
D1_TR_NA_AB674414_NA_
D1_IR_2006_HM229732_NA_
D1_IR_2006_HM229740_NA_
D1_IR_NA_KC176107_NA_
D1_TR_NA_AB674428_NA_
D1_TR_NA_AB674434_NA_
MN106402_Russia_2018
D1_IR_NA_HM348669_Kermanshah_
D1_TR_2006_GQ486054_NA_
D1_IR_2006_HM229739_NA_
D1_IR_2007_KF471659_NA_
D1_IR_NA_HM358293_Tehran_
D1_IR_NA_KC176144_NA_
D1_IR_NA_KC339907_NA_
D1_IR_NA_KC176148_NA_
MK173128_Afghanistan_2010
MK173097_Norway_2010
MK173158_Afghanistan_2005
MW234354_Pakistan_2019
D1_TR_2006_GQ486254_NA_
D1_IR_2013_KY225832_NA_
D1_IR_2013_KY225833_NA_
D1_IR_2013_KY225835_NA_
D1_IR_2013_KY225836_NA_
D1_IR_2009_JN040797_NA_
D1_IR_NA_HM348684_Khouzestan_
KX372161_Belgium_2010
KX659396_Netherlands_2005
KY210483_Uzbekistan_2015
D1_LB_2010_JN642134_NA_
D1_IR_NA_HM358303_Tehran_
D1_IR_2007_GU456640_NA_
JN664920_India_NA
JX849576_Italy_NA
JN226089_Italy_2010
D1_IR_2007_GU456658_NA_
D1_IR_NA_KC339814_NA_
D1_IR_2014_KT355694_NA_
D1_IR_NA_KC339921_NA_
D1_SD_2008_KF170766_NA_
D1_SD_2011_KM108605_NA_
D1_TN_2005_FJ904354_NA_
MH243307_Kazakhstan_2012
MK598643_Russia_2015
MK618428_Russia_2016
D1_IR_2014_KT355699_NA_
MN507851_Guinea_2018
D1_IR_NA_KC339899_NA_
D1_IR_NA_KC339901_NA_
D1_IR_NA_KC339900_NA_

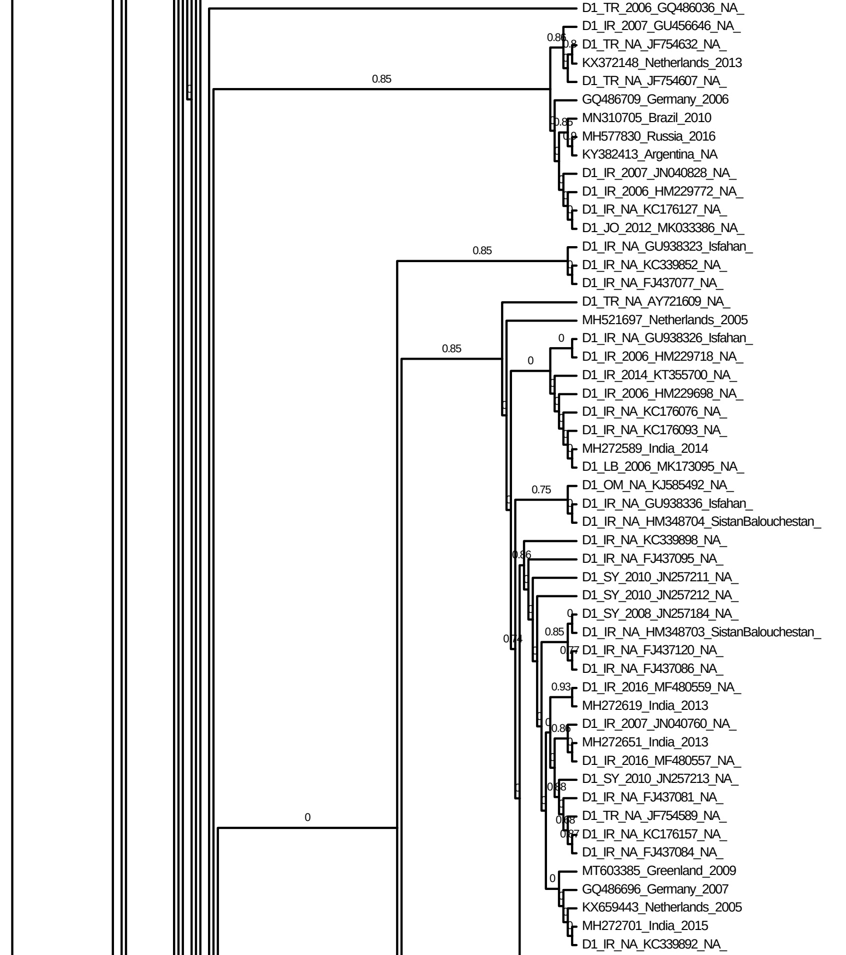
D1_TR_2006_GQ486036_NA_
D1_IR_2007_GU456646_NA_
D1_TR_NA_JF754632_NA_
KX372148_Netherlands_2013
D1_TR_NA_JF754607_NA_
GQ486709_Germany_2006
MN310705_Brazil_2010
MH577830_Russia_2016
KY382413_Argentina_NA
D1_IR_2007_JN040828_NA_
D1_IR_2006_HM229772_NA_
D1_IR_NA_KC176127_NA_
D1_JO_2012_MK033386_NA_
D1_IR_NA_GU938323_Isfahan_
D1_IR_NA_KC339852_NA_
D1_IR_NA_FJ437077_NA_
D1_TR_NA_AY721609_NA_
MH521697_Netherlands_2005
D1_IR_NA_GU938326_Isfahan_
D1_IR_2006_HM229718_NA_
D1_IR_2014_KT355700_NA_
D1_IR_2006_HM229698_NA_
D1_IR_NA_KC176076_NA_
D1_IR_NA_KC176093_NA_
MH272589_India_2014
D1_LB_2006_MK173095_NA_
D1_OM_NA_KJ585492_NA_
D1_IR_NA_GU938336_Isfahan_
D1_IR_NA_HM348704_SistanBalouchestan_
D1_IR_NA_KC339898_NA_
D1_IR_NA_FJ437095_NA_
D1_SY_2010_JN257211_NA_
D1_SY_2010_JN257212_NA_
D1_SY_2008_JN257184_NA_
D1_IR_NA_HM348703_SistanBalouchestan_
D1_IR_NA_FJ437120_NA_
D1_IR_NA_FJ437086_NA_
D1_IR_2016_MF480559_NA_
MH272619_India_2013
D1_IR_2007_JN040760_NA_
MH272651_India_2013
D1_IR_2016_MF480557_NA_
D1_SY_2010_JN257213_NA_
D1_IR_NA_FJ437081_NA_
D1_TR_NA_JF754589_NA_
D1_IR_NA_KC176157_NA_
D1_IR_NA_FJ437084_NA_
MT603385_Greenland_2009
GQ486696_Germany_2007
KX659443_Netherlands_2005
MH272701_India_2015
D1_IR_NA_KC339892_NA_

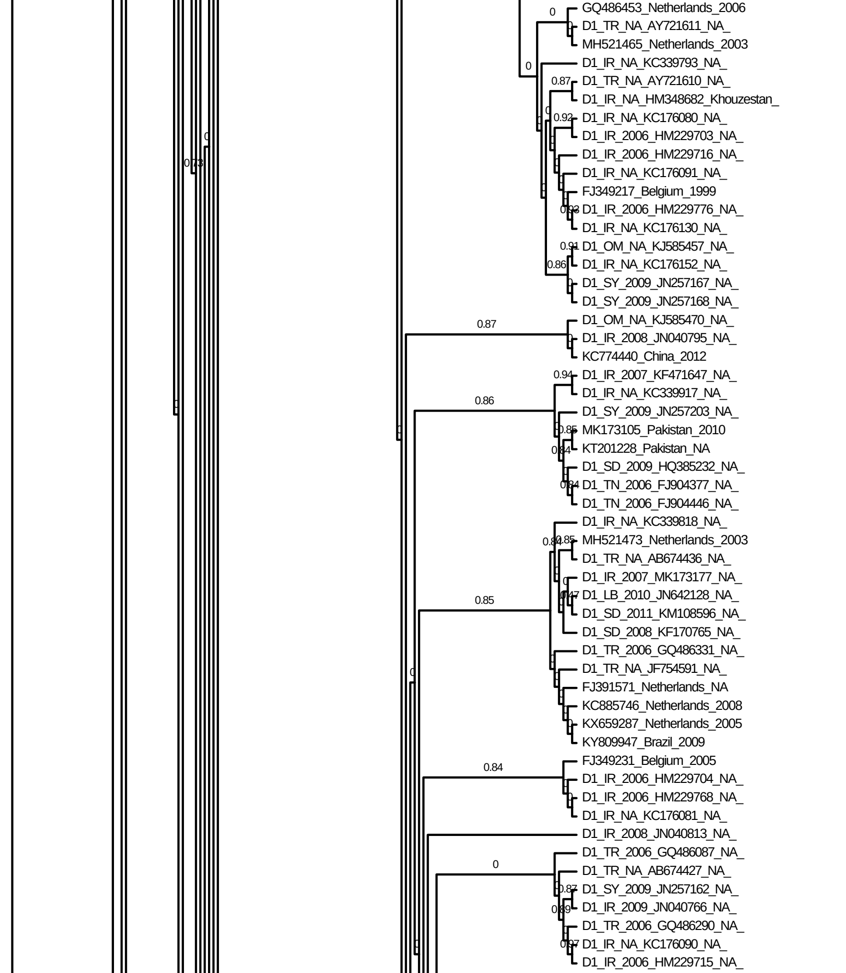
GQ486453_Netherlands_2006
D1_TR_NA_AY721611_NA_
MH521465_Netherlands_2003
D1_IR_NA_KC339793_NA_
D1_TR_NA_AY721610_NA_
D1_IR_NA_HM348682_Khouzestan_
D1_IR_NA_KC176080_NA_
D1_IR_2006_HM229703_NA_
D1_IR_2006_HM229716_NA_
D1_IR_NA_KC176091_NA_
FJ349217_Belgium_1999
D1_IR_2006_HM229776_NA_
D1_IR_NA_KC176130_NA_
D1_OM_NA_KJ585457_NA_
D1_IR_NA_KC176152_NA_
D1_SY_2009_JN257167_NA_
D1_SY_2009_JN257168_NA_
0.87
D1_OM_NA_KJ585470_NA_
D1_IR_2008_JN040795_NA_
KC774440_China_2012
D1_IR_2007_KF471647_NA_
D1_IR_NA_KC339917_NA_
0.86
D1_SY_2009_JN257203_NA_
MK173105_Pakistan_2010
KT201228_Pakistan_NA
D1_SD_2009_HQ385232_NA_
D1_TN_2006_FJ904377_NA_
D1_TN_2006_FJ904446_NA_
D1_IR_NA_KC339818_NA_
MH521473_Netherlands_2003
D1_TR_NA_AB674436_NA_
D1_IR_2007_MK173177_NA_
D1_LB_2010_JN642128_NA_
0.85
D1_SD_2011_KM108596_NA_
D1_SD_2008_KF170765_NA_
D1_TR_2006_GQ486331_NA_
D1_TR_NA_JF754591_NA_
FJ391571_Netherlands_NA
KC885746_Netherlands_2008
KX659287_Netherlands_2005
KY809947_Brazil_2009
0.84
FJ349231_Belgium_2005
D1_IR_2006_HM229704_NA_
D1_IR_2006_HM229768_NA_
D1_IR_NA_KC176081_NA_
D1_IR_2008_JN040813_NA_
0
D1_TR_2006_GQ486087_NA_
D1_TR_NA_AB674427_NA_
D1_SY_2009_JN257162_NA_
D1_IR_2009_JN040766_NA_
D1_TR_2006_GQ486290_NA_
D1_IR_NA_KC176090_NA_
D1_IR_2006_HM229715_NA_

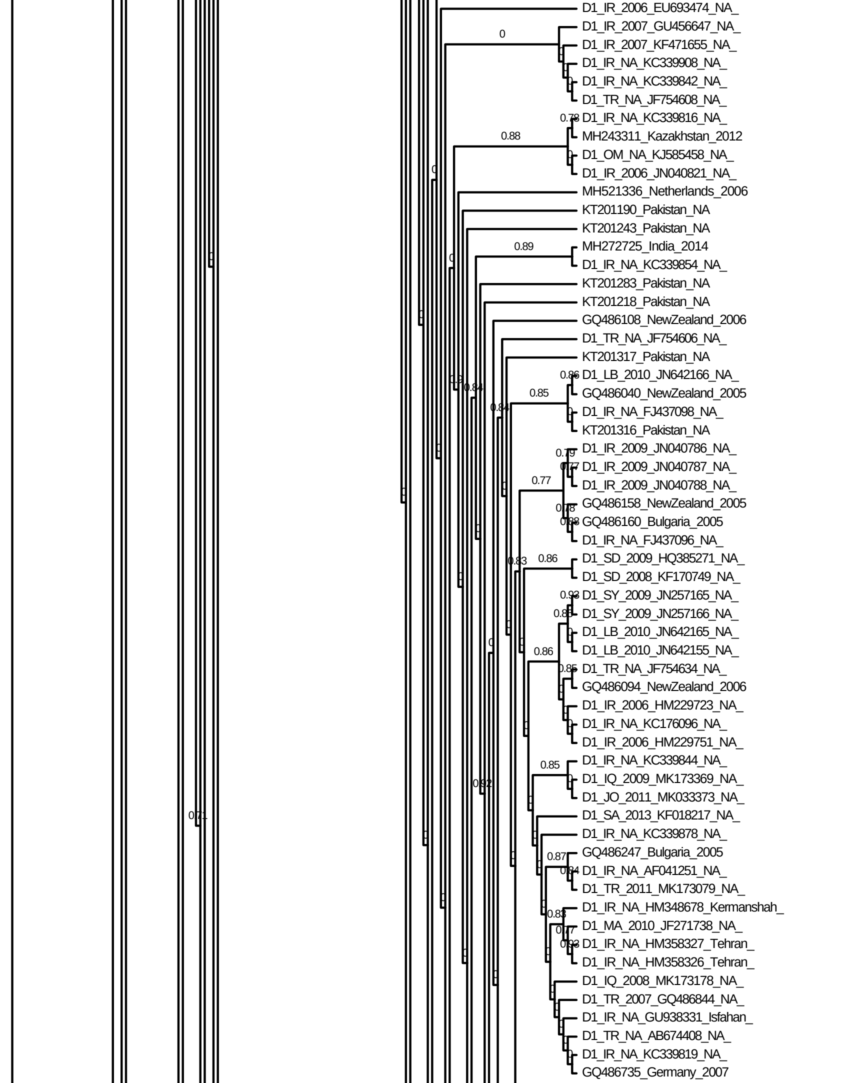
D1_IR_2006_EU693474_NA_
D1_IR_2007_GU456647_NA_
D1_IR_2007_KF471655_NA_
D1_IR_NA_KC339908_NA_
D1_IR_NA_KC339842_NA_
D1_TR_NA_JF754608_NA_
D1_IR_NA_KC339816_NA_
MH243311_Kazakhstan_2012
D1_OM_NA_KJ585458_NA_
D1_IR_2006_JN040821_NA_
MH521336_Netherlands_2006
KT201190_Pakistan_NA
KT201243_Pakistan_NA
MH272725_India_2014
D1_IR_NA_KC339854_NA_
KT201283_Pakistan_NA
KT201218_Pakistan_NA
GQ486108_NewZealand_2006
D1_TR_NA_JF754606_NA_
KT201317_Pakistan_NA
D1_LB_2010_JN642166_NA_
GQ486040_NewZealand_2005
D1_IR_NA_FJ437098_NA_
KT201316_Pakistan_NA
D1_IR_2009_JN040786_NA_
D1_IR_2009_JN040787_NA_
D1_IR_2009_JN040788_NA_
GQ486158_NewZealand_2005
GQ486160_Bulgaria_2005
D1_IR_NA_FJ437096_NA_
D1_SD_2009_HQ385271_NA_
D1_SD_2008_KF170749_NA_
D1_SY_2009_JN257165_NA_
D1_SY_2009_JN257166_NA_
D1_LB_2010_JN642165_NA_
D1_LB_2010_JN642155_NA_
D1_TR_NA_JF754634_NA_
GQ486094_NewZealand_2006
D1_IR_2006_HM229723_NA_
D1_IR_NA_KC176096_NA_
D1_IR_2006_HM229751_NA_
D1_IR_NA_KC339844_NA_
D1_IQ_2009_MK173369_NA_
D1_JO_2011_MK033373_NA_
D1_SA_2013_KF018217_NA_
D1_IR_NA_KC339878_NA_
GQ486247_Bulgaria_2005
D1_IR_NA_AF041251_NA_
D1_TR_2011_MK173079_NA_
D1_IR_NA_HM348678_Kermanshah_
D1_MA_2010_JF271738_NA_
D1_IR_NA_HM358327_Tehran_
D1_IR_NA_HM358326_Tehran_
D1_IQ_2008_MK173178_NA_
D1_TR_2007_GQ486844_NA_
D1_IR_NA_GU938331_Isfahan_
D1_TR_NA_AB674408_NA_
D1_IR_NA_KC339819_NA_
GQ486735_Germany_2007

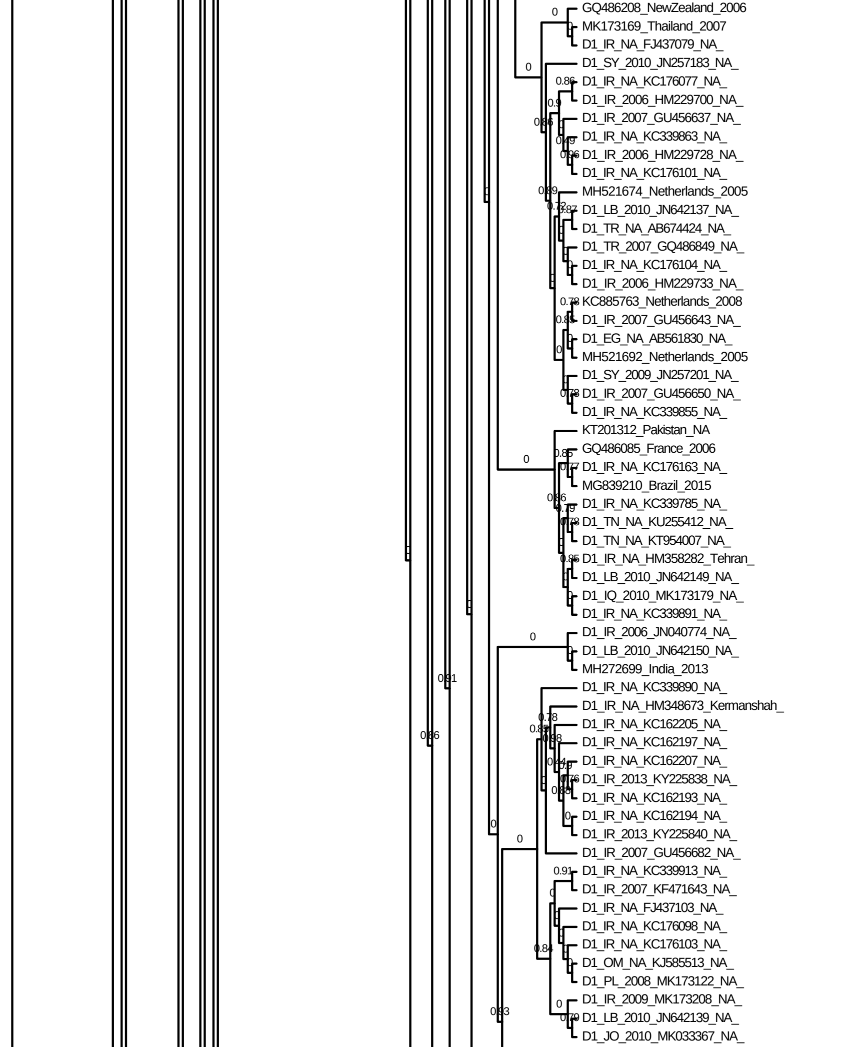
GQ486208_NewZealand_2006
MK173169_Thailand_2007
D1_IR_NA_FJ437079_NA_
D1_SY_2010_JN257183_NA_
D1_IR_NA_KC176077_NA_
D1_IR_2006_HM229700_NA_
D1_IR_2007_GU456637_NA_
D1_IR_NA_KC339863_NA_
D1_IR_2006_HM229728_NA_
D1_IR_NA_KC176101_NA_
MH521674_Netherlands_2005
D1_LB_2010_JN642137_NA_
D1_TR_NA_AB674424_NA_
D1_TR_2007_GQ486849_NA_
D1_IR_NA_KC176104_NA_
D1_IR_2006_HM229733_NA_
KC885763_Netherlands_2008
D1_IR_2007_GU456643_NA_
D1_EG_NA_AB561830_NA_
MH521692_Netherlands_2005
D1_SY_2009_JN257201_NA_
D1_IR_2007_GU456650_NA_
D1_IR_NA_KC339855_NA_
KT201312_Pakistan_NA
GQ486085_France_2006
D1_IR_NA_KC176163_NA_
MG839210_Brazil_2015
D1_IR_NA_KC339785_NA_
D1_TN_NA_KU255412_NA_
D1_TN_NA_KT954007_NA_
D1_IR_NA_HM358282_Tehran_
D1_LB_2010_JN642149_NA_
D1_IQ_2010_MK173179_NA_
D1_IR_NA_KC339891_NA_
D1_IR_2006_JN040774_NA_
D1_LB_2010_JN642150_NA_
MH272699_India_2013
D1_IR_NA_KC339890_NA_
D1_IR_NA_HM348673_Kermanshah_
D1_IR_NA_KC162205_NA_
D1_IR_NA_KC162197_NA_
D1_IR_NA_KC162207_NA_
D1_IR_2013_KY225838_NA_
D1_IR_NA_KC162193_NA_
D1_IR_NA_KC162194_NA_
D1_IR_2013_KY225840_NA_
D1_IR_2007_GU456682_NA_
D1_IR_NA_KC339913_NA_
D1_IR_2007_KF471643_NA_
D1_IR_NA_FJ437103_NA_
D1_IR_NA_KC176098_NA_
D1_IR_NA_KC176103_NA_
D1_OM_NA_KJ585513_NA_
D1_PL_2008_MK173122_NA_
D1_IR_2009_MK173208_NA_
D1_LB_2010_JN642139_NA_
D1_JO_2010_MK033367_NA_

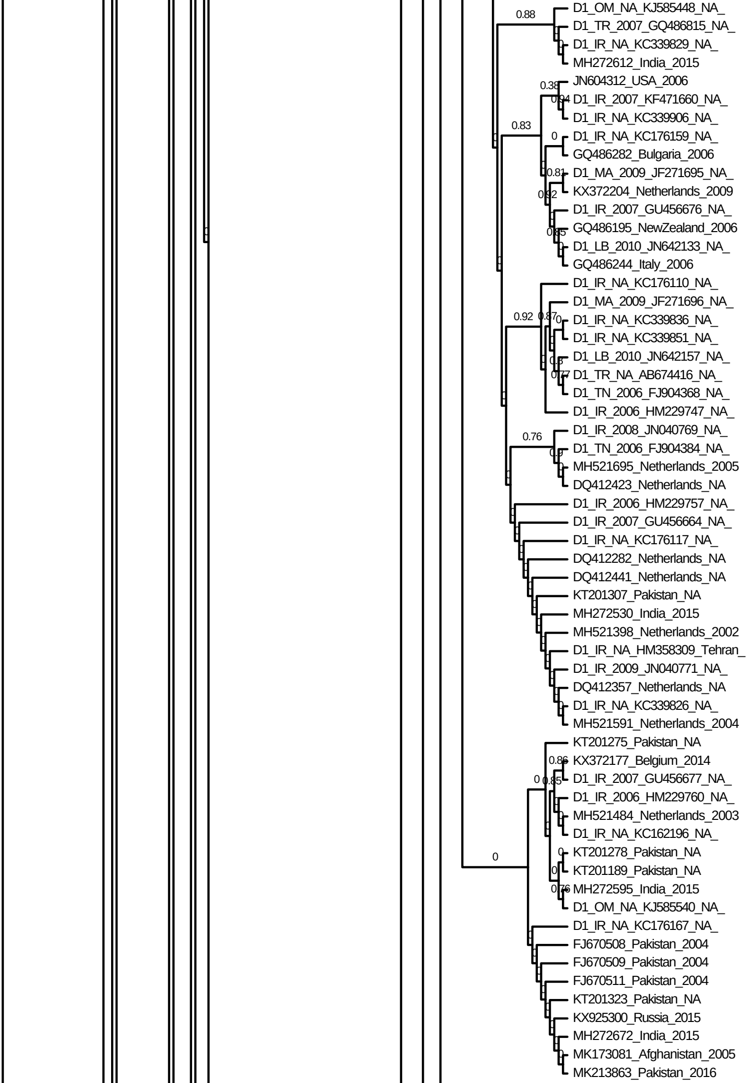

0.88
D1_OM_NA_KJ585448_NA_
D1_TR_2007_GQ486815_NA_
D1_IR_NA_KC339829_NA_
MH272612_India_2015
JN604312_USA_2006
D1_IR_2007_KF471660_NA_
D1_IR_NA_KC339906_NA_
0.83
D1_IR_NA_KC176159_NA_
GQ486282_Bulgaria_2006
D1_MA_2009_JF271695_NA_
KX372204_Netherlands_2009
D1_IR_2007_GU456676_NA_
GQ486195_NewZealand_2006
D1_LB_2010_JN642133_NA_
GQ486244_Italy_2006
D1_IR_NA_KC176110_NA_
D1_MA_2009_JF271696_NA_
0.92
D1_IR_NA_KC339836_NA_
D1_IR_NA_KC339851_NA_
D1_LB_2010_JN642157_NA_
D1_TR_NA_AB674416_NA_
D1_TN_2006_FJ904368_NA_
D1_IR_2006_HM229747_NA_
0.76
D1_IR_2008_JN040769_NA_
D1_TN_2006_FJ904384_NA_
MH521695_Netherlands_2005
DQ412423_Netherlands_NA
D1_IR_2006_HM229757_NA_
D1_IR_2007_GU456664_NA_
D1_IR_NA_KC176117_NA_
DQ412282_Netherlands_NA
DQ412441_Netherlands_NA
KT201307_Pakistan_NA
MH272530_India_2015
MH521398_Netherlands_2002
D1_IR_NA_HM358309_Tehran_
D1_IR_2009_JN040771_NA_
DQ412357_Netherlands_NA
D1_IR_NA_KC339826_NA_
MH521591_Netherlands_2004
KT201275_Pakistan_NA
KX372177_Belgium_2014
D1_IR_2007_GU456677_NA_
D1_IR_2006_HM229760_NA_
MH521484_Netherlands_2003
D1_IR_NA_KC162196_NA_
0
KT201278_Pakistan_NA
KT201189_Pakistan_NA
MH272595_India_2015
D1_OM_NA_KJ585540_NA_
D1_IR_NA_KC176167_NA_
FJ670508_Pakistan_2004
FJ670509_Pakistan_2004
FJ670511_Pakistan_2004
KT201323_Pakistan_NA
KX925300_Russia_2015
MH272672_India_2015
MK173081_Afghanistan_2005
MK213863_Pakistan_2016

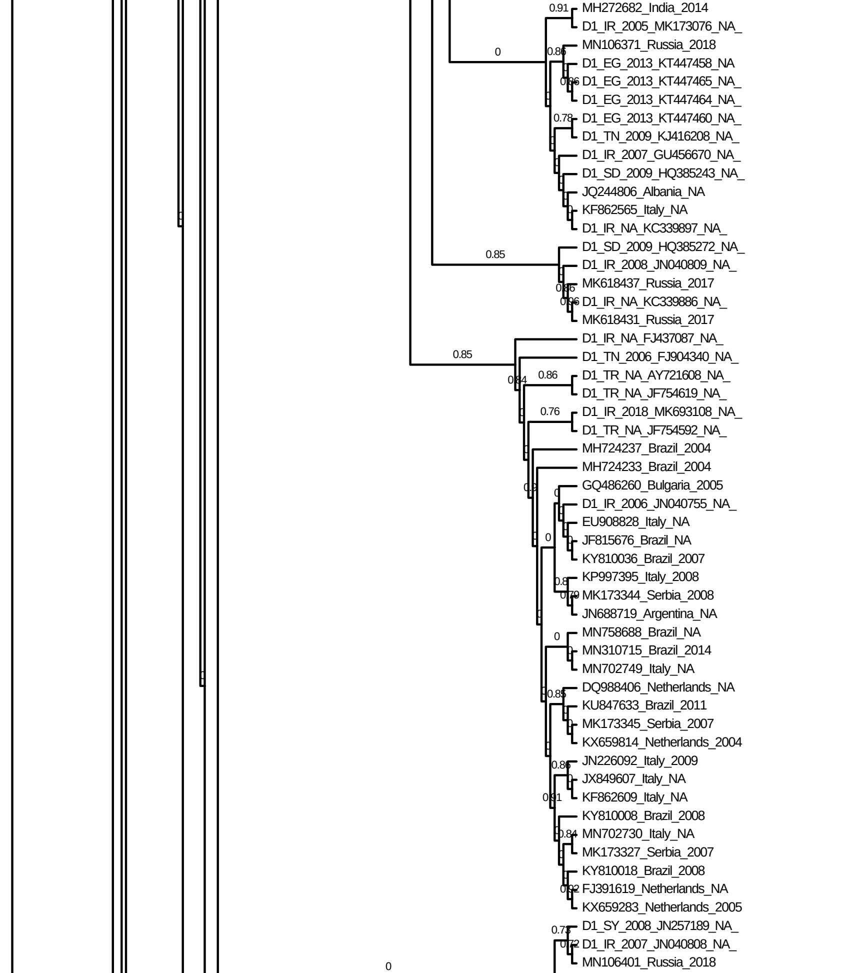
MH272682_India_2014
D1_IR_2005_MK173076_NA_
MN106371_Russia_2018
D1_EG_2013_KT447458_NA_
D1_EG_2013_KT447465_NA_
D1_EG_2013_KT447464_NA_
D1_EG_2013_KT447460_NA_
D1_TN_2009_KJ416208_NA_
D1_IR_2007_GU456670_NA_
D1_SD_2009_HQ385243_NA_
JQ244806_Albania_NA
KF862565_Italy_NA
D1_IR_NA_KC339897_NA_
D1_SD_2009_HQ385272_NA_
D1_IR_2008_JN040809_NA_
MK618437_Russia_2017
D1_IR_NA_KC339886_NA_
MK618431_Russia_2017
D1_IR_NA_FJ437087_NA_
D1_TN_2006_FJ904340_NA_
D1_TR_NA_AY721608_NA_
D1_TR_NA_JF754619_NA_
D1_IR_2018_MK693108_NA_
D1_TR_NA_JF754592_NA_
MH724237_Brazil_2004
MH724233_Brazil_2004
GQ486260_Bulgaria_2005
D1_IR_2006_JN040755_NA_
EU908828_Italy_NA
JF815676_Brazil_NA
KY810036_Brazil_2007
KP997395_Italy_2008
MK173344_Serbia_2008
JN688719_Argentina_NA
MN758688_Brazil_NA
MN310715_Brazil_2014
MN702749_Italy_NA
DQ988406_Netherlands_NA
KU847633_Brazil_2011
MK173345_Serbia_2007
KX659814_Netherlands_2004
JN226092_Italy_2009
JX849607_Italy_NA
KF862609_Italy_NA
KY810008_Brazil_2008
MN702730_Italy_NA
MK173327_Serbia_2007
KY810018_Brazil_2008
FJ391619_Netherlands_NA
KX659283_Netherlands_2005
D1_SY_2008_JN257189_NA_
D1_IR_2007_JN040808_NA_
MN106401_Russia_2018

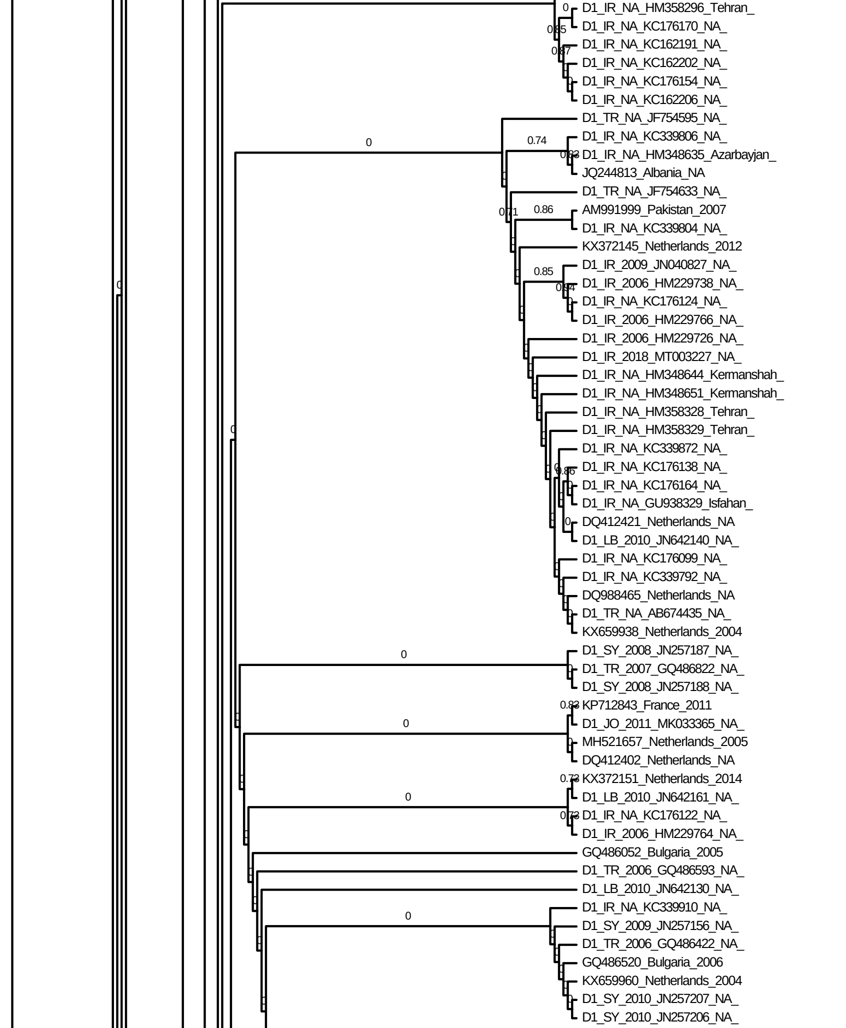

D1_IR_NA_HM358296_Tehran_
D1_IR_NA_KC176170_NA_
D1_IR_NA_KC162191_NA_
D1_IR_NA_KC162202_NA_
D1_IR_NA_KC176154_NA_
D1_IR_NA_KC162206_NA_
D1_TR_NA_JF754595_NA_
D1_IR_NA_KC339806_NA_
D1_IR_NA_HM348635_Azarbayjan_
JQ244813_Albania_NA
D1_TR_NA_JF754633_NA_
AM991999_Pakistan_2007
D1_IR_NA_KC339804_NA_
KX372145_Netherlands_2012
D1_IR_2009_JN040827_NA_
D1_IR_2006_HM229738_NA_
D1_IR_NA_KC176124_NA_
D1_IR_2006_HM229766_NA_
D1_IR_2006_HM229726_NA_
D1_IR_2018_MT003227_NA_
D1_IR_NA_HM348644_Kermanshah_
D1_IR_NA_HM348651_Kermanshah_
D1_IR_NA_HM358328_Tehran_
D1_IR_NA_HM358329_Tehran_
D1_IR_NA_KC339872_NA_
D1_IR_NA_KC176138_NA_
D1_IR_NA_KC176164_NA_
D1_IR_NA_GU938329_Isfahan_
DQ412421_Netherlands_NA
D1_LB_2010_JN642140_NA_
D1_IR_NA_KC176099_NA_
D1_IR_NA_KC339792_NA_
DQ988465_Netherlands_NA
D1_TR_NA_AB674435_NA_
KX659938_Netherlands_2004
D1_SY_2008_JN257187_NA_
D1_TR_2007_GQ486822_NA_
D1_SY_2008_JN257188_NA_
KP712843_France_2011
D1_JO_2011_MK033365_NA_
MH521657_Netherlands_2005
DQ412402_Netherlands_NA
KX372151_Netherlands_2014
D1_LB_2010_JN642161_NA_
D1_IR_NA_KC176122_NA_
D1_IR_2006_HM229764_NA_
GQ486052_Bulgaria_2005
D1_TR_2006_GQ486593_NA_
D1_LB_2010_JN642130_NA_
D1_IR_NA_KC339910_NA_
D1_SY_2009_JN257156_NA_
D1_TR_2006_GQ486422_NA_
GQ486520_Bulgaria_2006
KX659960_Netherlands_2004
D1_SY_2010_JN257207_NA_
D1_SY_2010_JN257206_NA_

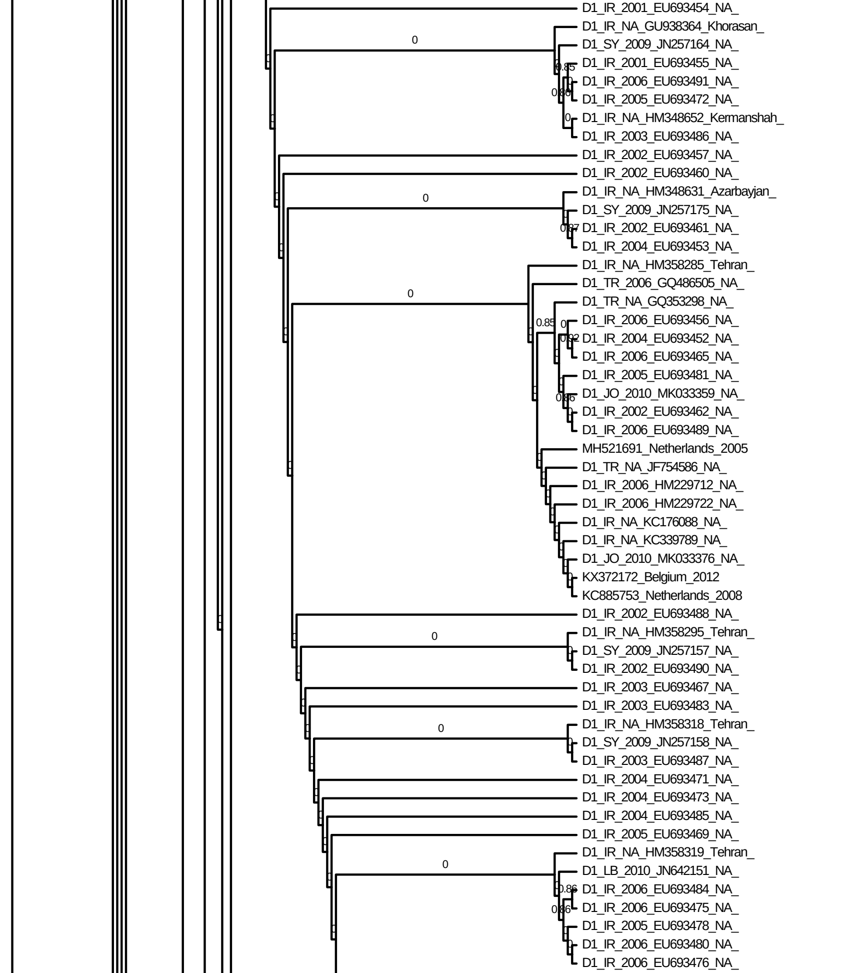
D1_IR_2001_EU693454_NA_
D1_IR_NA_GU938364_Khorasan_
D1_SY_2009_JN257164_NA_
D1_IR_2001_EU693455_NA_
D1_IR_2006_EU693491_NA_
D1_IR_2005_EU693472_NA_
D1_IR_NA_HM348652_Kermanshah_
D1_IR_2003_EU693486_NA_
D1_IR_2002_EU693457_NA_
D1_IR_2002_EU693460_NA_
D1_IR_NA_HM348631_Azarbayjan_
D1_SY_2009_JN257175_NA_
D1_IR_2002_EU693461_NA_
D1_IR_2004_EU693453_NA_
D1_IR_NA_HM358285_Tehran_
D1_TR_2006_GQ486505_NA_
D1_TR_NA_GQ353298_NA_
D1_IR_2006_EU693456_NA_
D1_IR_2004_EU693452_NA_
D1_IR_2006_EU693465_NA_
D1_IR_2005_EU693481_NA_
D1_JO_2010_MK033359_NA_
D1_IR_2002_EU693462_NA_
D1_IR_2006_EU693489_NA_
MH521691_Netherlands_2005
D1_TR_NA_JF754586_NA_
D1_IR_2006_HM229712_NA_
D1_IR_2006_HM229722_NA_
D1_IR_NA_KC176088_NA_
D1_IR_NA_KC339789_NA_
D1_JO_2010_MK033376_NA_
KX372172_Belgium_2012
KC885753_Netherlands_2008
D1_IR_2002_EU693488_NA_
D1_IR_NA_HM358295_Tehran_
D1_SY_2009_JN257157_NA_
D1_IR_2002_EU693490_NA_
D1_IR_2003_EU693467_NA_
D1_IR_2003_EU693483_NA_
D1_IR_NA_HM358318_Tehran_
D1_SY_2009_JN257158_NA_
D1_IR_2003_EU693487_NA_
D1_IR_2004_EU693471_NA_
D1_IR_2004_EU693473_NA_
D1_IR_2004_EU693485_NA_
D1_IR_2005_EU693469_NA_
D1_IR_NA_HM358319_Tehran_
D1_LB_2010_JN642151_NA_
D1_IR_2006_EU693484_NA_
D1_IR_2006_EU693475_NA_
D1_IR_2005_EU693478_NA_
D1_IR_2006_EU693480_NA_
D1_IR_2006_EU693476_NA_

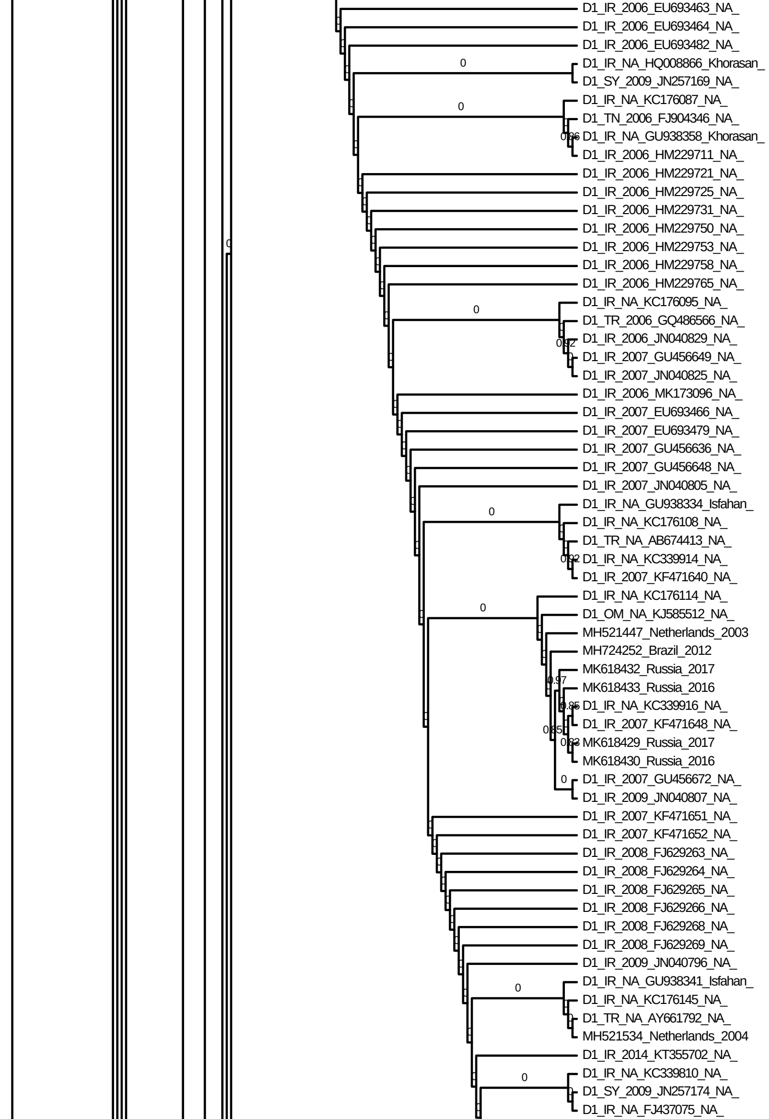
D1_IR_2006_EU693463_NA_
D1_IR_2006_EU693464_NA_
D1_IR_2006_EU693482_NA_
D1_IR_NA_HQ008866_Khorasan_
D1_SY_2009_JN257169_NA_
D1_IR_NA_KC176087_NA_
D1_TN_2006_FJ904346_NA_
D1_IR_NA_GU938358_Khorasan_
D1_IR_2006_HM229711_NA_
D1_IR_2006_HM229721_NA_
D1_IR_2006_HM229725_NA_
D1_IR_2006_HM229731_NA_
D1_IR_2006_HM229750_NA_
D1_IR_2006_HM229753_NA_
D1_IR_2006_HM229758_NA_
D1_IR_2006_HM229765_NA_
D1_IR_NA_KC176095_NA_
D1_TR_2006_GQ486566_NA_
D1_IR_2006_JN040829_NA_
D1_IR_2007_GU456649_NA_
D1_IR_2007_JN040825_NA_
D1_IR_2006_MK173096_NA_
D1_IR_2007_EU693466_NA_
D1_IR_2007_EU693479_NA_
D1_IR_2007_GU456636_NA_
D1_IR_2007_GU456648_NA_
D1_IR_2007_JN040805_NA_
D1_IR_NA_GU938334_Isfahan_
D1_IR_NA_KC176108_NA_
D1_TR_NA_AB674413_NA_
D1_IR_NA_KC339914_NA_
D1_IR_2007_KF471640_NA_
D1_IR_NA_KC176114_NA_
D1_OM_NA_KJ585512_NA_
MH521447_Netherlands_2003
MH724252_Brazil_2012
MK618432_Russia_2017
MK618433_Russia_2016
D1_IR_NA_KC339916_NA_
D1_IR_2007_KF471648_NA_
MK618429_Russia_2017
MK618430_Russia_2016
D1_IR_2007_GU456672_NA_
D1_IR_2009_JN040807_NA_
D1_IR_2007_KF471651_NA_
D1_IR_2007_KF471652_NA_
D1_IR_2008_FJ629263_NA_
D1_IR_2008_FJ629264_NA_
D1_IR_2008_FJ629265_NA_
D1_IR_2008_FJ629266_NA_
D1_IR_2008_FJ629268_NA_
D1_IR_2008_FJ629269_NA_
D1_IR_2009_JN040796_NA_
D1_IR_NA_GU938341_Isfahan_
D1_IR_NA_KC176145_NA_
D1_TR_NA_AY661792_NA_
MH521534_Netherlands_2004
D1_IR_2014_KT355702_NA_
D1_IR_NA_KC339810_NA_
D1_SY_2009_JN257174_NA_
D1_IR_NA_FJ437075_NA_

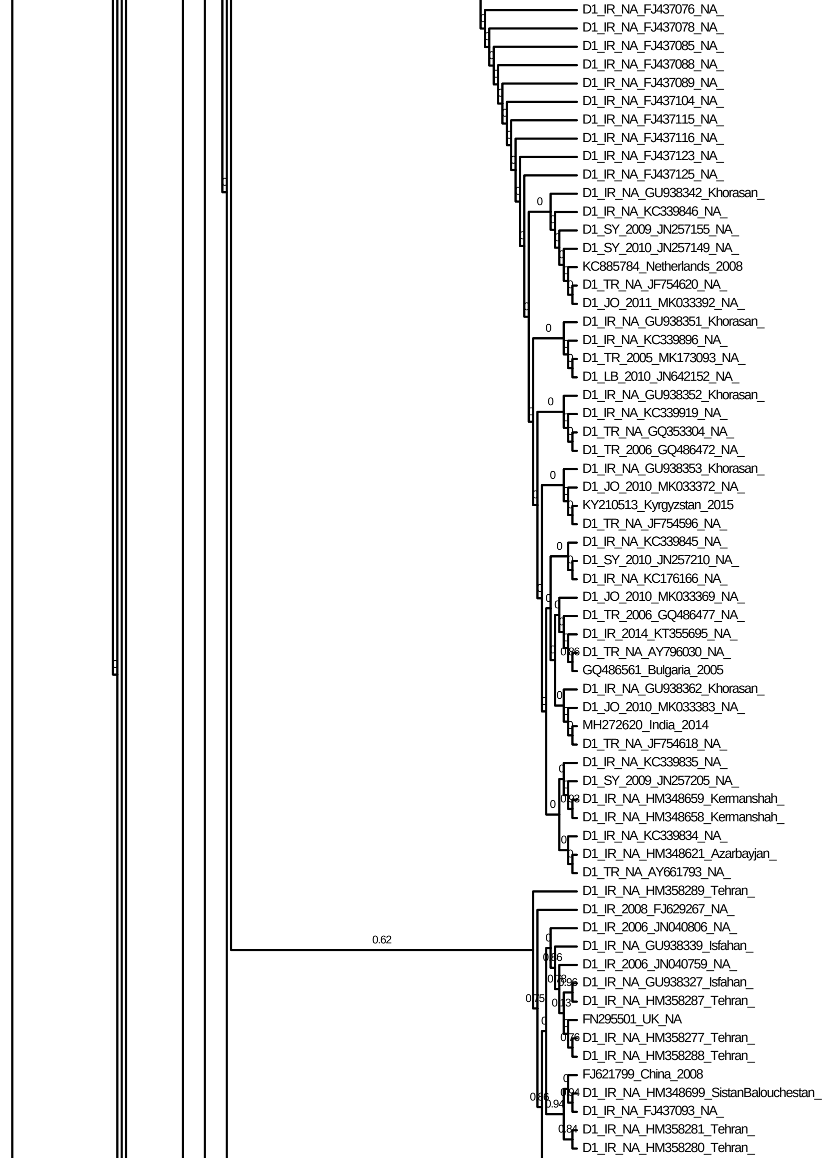

D1_IR_NA_FJ437076_NA_
D1_IR_NA_FJ437078_NA_
D1_IR_NA_FJ437085_NA_
D1_IR_NA_FJ437088_NA_
D1_IR_NA_FJ437089_NA_
D1_IR_NA_FJ437104_NA_
D1_IR_NA_FJ437115_NA_
D1_IR_NA_FJ437116_NA_
D1_IR_NA_FJ437123_NA_
D1_IR_NA_FJ437125_NA_
D1_IR_NA_GU938342_Khorasan_
D1_IR_NA_KC339846_NA_
D1_SY_2009_JN257155_NA_
D1_SY_2010_JN257149_NA_
KC885784_Netherlands_2008
D1_TR_NA_JF754620_NA_
D1_JO_2011_MK033392_NA_
D1_IR_NA_GU938351_Khorasan_
D1_IR_NA_KC339896_NA_
D1_TR_2005_MK173093_NA_
D1_LB_2010_JN642152_NA_
D1_IR_NA_GU938352_Khorasan_
D1_IR_NA_KC339919_NA_
D1_TR_NA_GQ353304_NA_
D1_TR_2006_GQ486472_NA_
D1_IR_NA_GU938353_Khorasan_
D1_JO_2010_MK033372_NA_
KY210513_Kyrgyzstan_2015
D1_TR_NA_JF754596_NA_
D1_IR_NA_KC339845_NA_
D1_SY_2010_JN257210_NA_
D1_IR_NA_KC176166_NA_
D1_JO_2010_MK033369_NA_
D1_TR_2006_GQ486477_NA_
D1_IR_2014_KT355695_NA_
D1_TR_NA_AY796030_NA_
GQ486561_Bulgaria_2005
D1_IR_NA_GU938362_Khorasan_
D1_JO_2010_MK033383_NA_
MH272620_India_2014
D1_TR_NA_JF754618_NA_
D1_IR_NA_KC339835_NA_
D1_SY_2009_JN257205_NA_
D1_IR_NA_HM348659_Kermanshah_
D1_IR_NA_HM348658_Kermanshah_
D1_IR_NA_KC339834_NA_
D1_IR_NA_HM348621_Azarbayjan_
D1_TR_NA_AY661793_NA_
D1_IR_NA_HM358289_Tehran_
D1_IR_2008_FJ629267_NA_
D1_IR_2006_JN040806_NA_
D1_IR_NA_GU938339_Isfahan_
D1_IR_2006_JN040759_NA_
D1_IR_NA_GU938327_Isfahan_
D1_IR_NA_HM358287_Tehran_
FN295501_UK_NA
D1_IR_NA_HM358277_Tehran_
D1_IR_NA_HM358288_Tehran_
FJ621799_China_2008
D1_IR_NA_HM348699_SistanBalouchestan_
D1_IR_NA_FJ437093_NA_
D1_IR_NA_HM358281_Tehran_
D1_IR_NA_HM358280_Tehran_
0.62
0.75
0.86
0.94
0.96
0.84
0.76

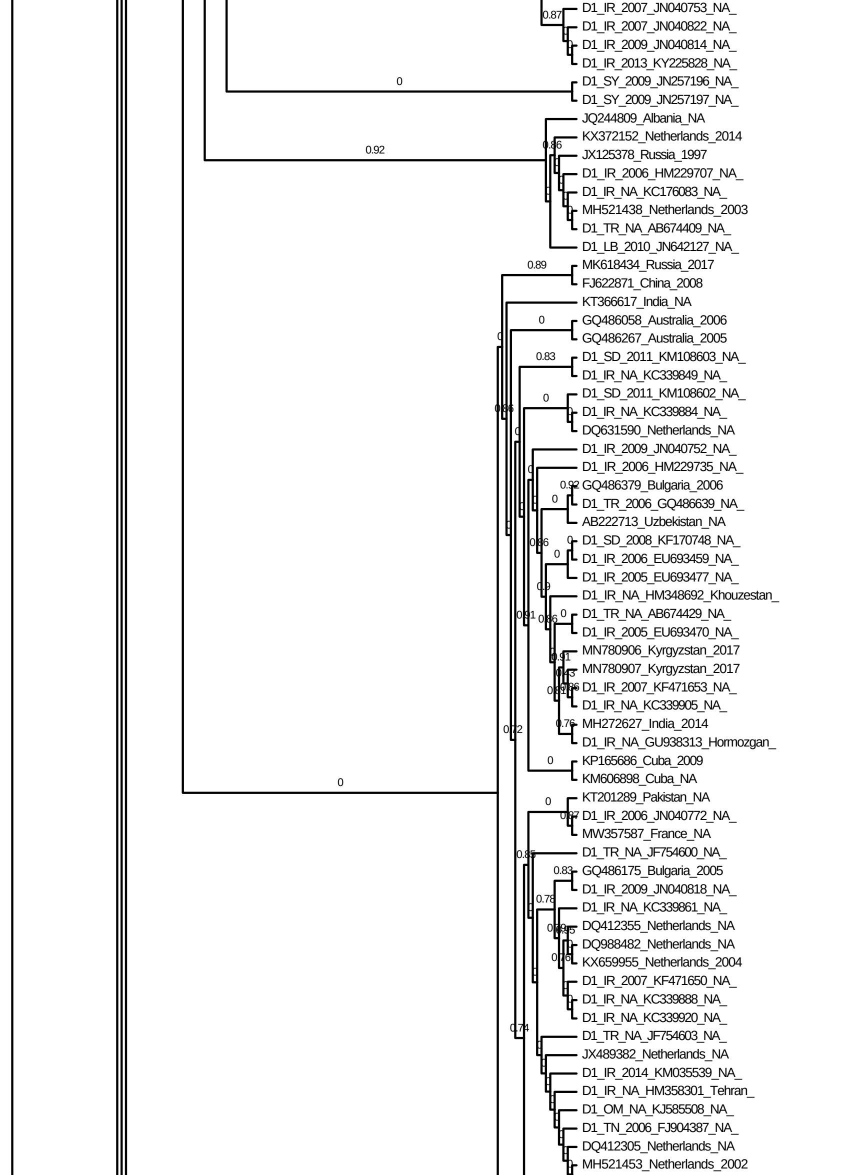
D1_IR_2007_JN040753_NA_
D1_IR_2007_JN040822_NA_
D1_IR_2009_JN040814_NA_
D1_IR_2013_KY225828_NA_
D1_SY_2009_JN257196_NA_
D1_SY_2009_JN257197_NA_
JQ244809_Albania_NA
KX372152_Netherlands_2014
JX125378_Russia_1997
D1_IR_2006_HM229707_NA_
D1_IR_NA_KC176083_NA_
MH521438_Netherlands_2003
D1_TR_NA_AB674409_NA_
D1_LB_2010_JN642127_NA_
MK618434_Russia_2017
FJ622871_China_2008
KT366617_India_NA
GQ486058_Australia_2006
GQ486267_Australia_2005
D1_SD_2011_KM108603_NA_
D1_IR_NA_KC339849_NA_
D1_SD_2011_KM108602_NA_
D1_IR_NA_KC339884_NA_
DQ631590_Netherlands_NA
D1_IR_2009_JN040752_NA_
D1_IR_2006_HM229735_NA_
GQ486379_Bulgaria_2006
D1_TR_2006_GQ486639_NA_
AB222713_Uzbekistan_NA
D1_SD_2008_KF170748_NA_
D1_IR_2006_EU693459_NA_
D1_IR_2005_EU693477_NA_
D1_IR_NA_HM348692_Khouzestan_
D1_TR_NA_AB674429_NA_
D1_IR_2005_EU693470_NA_
MN780906_Kyrgyzstan_2017
MN780907_Kyrgyzstan_2017
D1_IR_2007_KF471653_NA_
D1_IR_NA_KC339905_NA_
MH272627_India_2014
D1_IR_NA_GU938313_Hormozgan_
KP165686_Cuba_2009
KM606898_Cuba_NA
KT201289_Pakistan_NA
D1_IR_2006_JN040772_NA_
MW357587_France_NA
D1_TR_NA_JF754600_NA_
GQ486175_Bulgaria_2005
D1_IR_2009_JN040818_NA_
D1_IR_NA_KC339861_NA_
DQ412355_Netherlands_NA
DQ988482_Netherlands_NA
KX659955_Netherlands_2004
D1_IR_2007_KF471650_NA_
D1_IR_NA_KC339888_NA_
D1_IR_NA_KC339920_NA_
D1_TR_NA_JF754603_NA_
JX489382_Netherlands_NA
D1_IR_2014_KM035539_NA_
D1_IR_NA_HM358301_Tehran_
D1_OM_NA_KJ585508_NA_
D1_TN_2006_FJ904387_NA_
DQ412305_Netherlands_NA
MH521453_Netherlands_2002

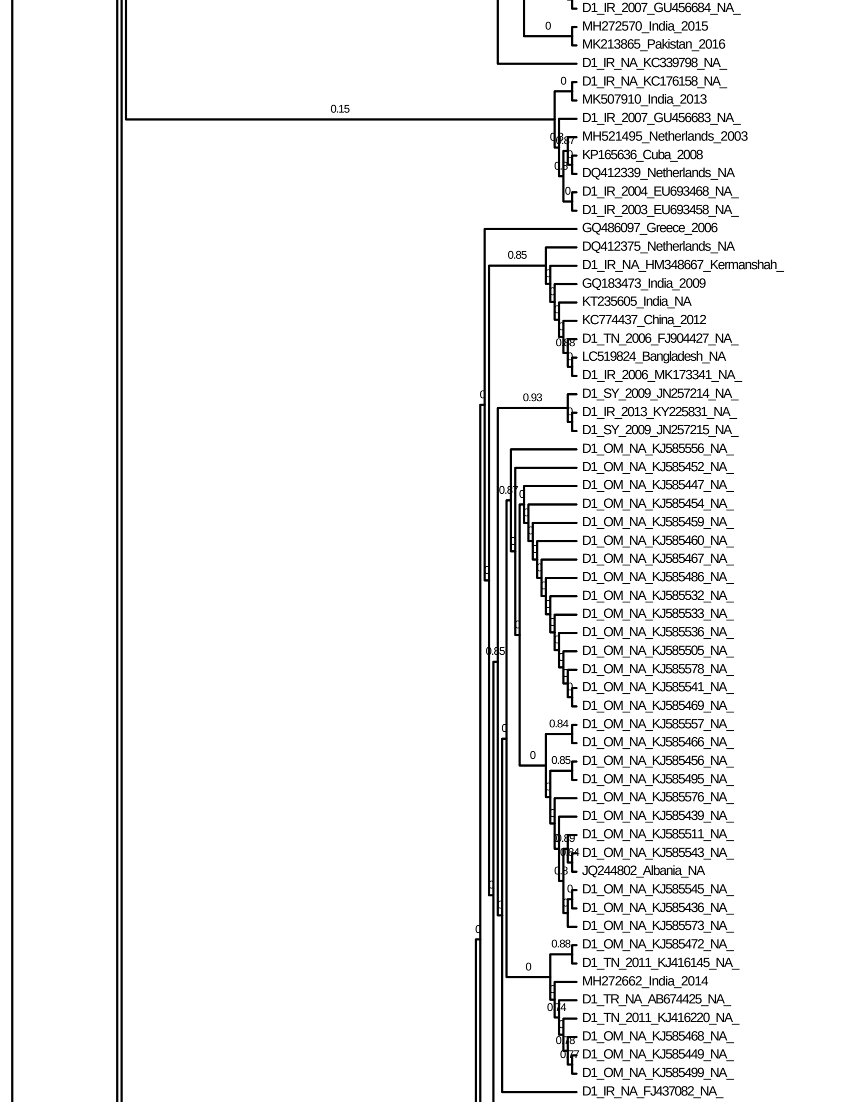
D1_IR_2007_GU456684_NA_
MH272570_India_2015
MK213865_Pakistan_2016
D1_IR_NA_KC339798_NA_
D1_IR_NA_KC176158_NA_
MK507910_India_2013
0.15
D1_IR_2007_GU456683_NA_
MH521495_Netherlands_2003
KP165636_Cuba_2008
DQ412339_Netherlands_NA
D1_IR_2004_EU693468_NA_
D1_IR_2003_EU693458_NA_
GQ486097_Greece_2006
DQ412375_Netherlands_NA
D1_IR_NA_HM348667_Kermanshah_
GQ183473_India_2009
KT235605_India_NA
KC774437_China_2012
D1_TN_2006_FJ904427_NA_
LC519824_Bangladesh_NA
D1_IR_2006_MK173341_NA_
D1_SY_2009_JN257214_NA_
D1_IR_2013_KY225831_NA_
D1_SY_2009_JN257215_NA_
D1_OM_NA_KJ585556_NA_
D1_OM_NA_KJ585452_NA_
D1_OM_NA_KJ585447_NA_
D1_OM_NA_KJ585454_NA_
D1_OM_NA_KJ585459_NA_
D1_OM_NA_KJ585460_NA_
D1_OM_NA_KJ585467_NA_
D1_OM_NA_KJ585486_NA_
D1_OM_NA_KJ585532_NA_
D1_OM_NA_KJ585533_NA_
D1_OM_NA_KJ585536_NA_
D1_OM_NA_KJ585505_NA_
D1_OM_NA_KJ585578_NA_
D1_OM_NA_KJ585541_NA_
D1_OM_NA_KJ585469_NA_
D1_OM_NA_KJ585557_NA_
D1_OM_NA_KJ585466_NA_
D1_OM_NA_KJ585456_NA_
D1_OM_NA_KJ585495_NA_
D1_OM_NA_KJ585576_NA_
D1_OM_NA_KJ585439_NA_
D1_OM_NA_KJ585511_NA_
D1_OM_NA_KJ585543_NA_
JQ244802_Albania_NA
D1_OM_NA_KJ585545_NA_
D1_OM_NA_KJ585436_NA_
D1_OM_NA_KJ585573_NA_
D1_OM_NA_KJ585472_NA_
D1_TN_2011_KJ416145_NA_
MH272662_India_2014
D1_TR_NA_AB674425_NA_
D1_TN_2011_KJ416220_NA_
D1_OM_NA_KJ585468_NA_
D1_OM_NA_KJ585449_NA_
D1_OM_NA_KJ585499_NA_
D1_IR_NA_FJ437082_NA_

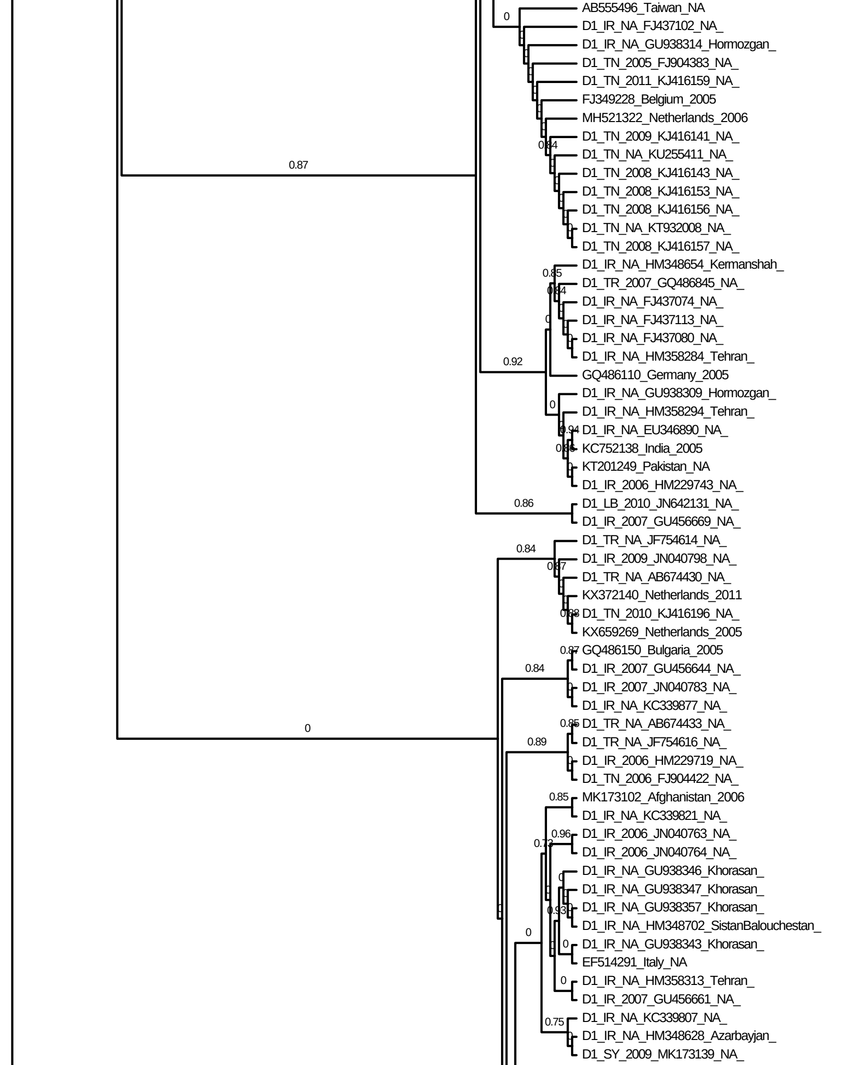
AB555496_Taiwan_NA
D1_IR_NA_FJ437102_NA_
D1_IR_NA_GU938314_Hormozgan_
D1_TN_2005_FJ904383_NA_
D1_TN_2011_KJ416159_NA_
FJ349228_Belgium_2005
MH521322_Netherlands_2006
D1_TN_2009_KJ416141_NA_
D1_TN_NA_KU255411_NA_
D1_TN_2008_KJ416143_NA_
D1_TN_2008_KJ416153_NA_
D1_TN_2008_KJ416156_NA_
D1_TN_NA_KT932008_NA_
D1_TN_2008_KJ416157_NA_
D1_IR_NA_HM348654_Kermanshah_
D1_TR_2007_GQ486845_NA_
D1_IR_NA_FJ437074_NA_
D1_IR_NA_FJ437113_NA_
D1_IR_NA_FJ437080_NA_
D1_IR_NA_HM358284_Tehran_
GQ486110_Germany_2005
D1_IR_NA_GU938309_Hormozgan_
D1_IR_NA_HM358294_Tehran_
D1_IR_NA_EU346890_NA_
KC752138_India_2005
KT201249_Pakistan_NA
D1_IR_2006_HM229743_NA_
D1_LB_2010_JN642131_NA_
D1_IR_2007_GU456669_NA_
D1_TR_NA_JF754614_NA_
D1_IR_2009_JN040798_NA_
D1_TR_NA_AB674430_NA_
KX372140_Netherlands_2011
D1_TN_2010_KJ416196_NA_
KX659269_Netherlands_2005
GQ486150_Bulgaria_2005
D1_IR_2007_GU456644_NA_
D1_IR_2007_JN040783_NA_
D1_IR_NA_KC339877_NA_
D1_TR_NA_AB674433_NA_
D1_TR_NA_JF754616_NA_
D1_IR_2006_HM229719_NA_
D1_TN_2006_FJ904422_NA_
MK173102_Afghanistan_2006
D1_IR_NA_KC339821_NA_
D1_IR_2006_JN040763_NA_
D1_IR_2006_JN040764_NA_
D1_IR_NA_GU938346_Khorasan_
D1_IR_NA_GU938347_Khorasan_
D1_IR_NA_GU938357_Khorasan_
D1_IR_NA_HM348702_SistanBalouchestan_
D1_IR_NA_GU938343_Khorasan_
EF514291_Italy_NA
D1_IR_NA_HM358313_Tehran_
D1_IR_2007_GU456661_NA_
D1_IR_NA_KC339807_NA_
D1_IR_NA_HM348628_Azarbayjan_
D1_SY_2009_MK173139_NA_
0.87
0.92
0.86
0.84
0.84
0.89
0
0.85
0.96
0.75

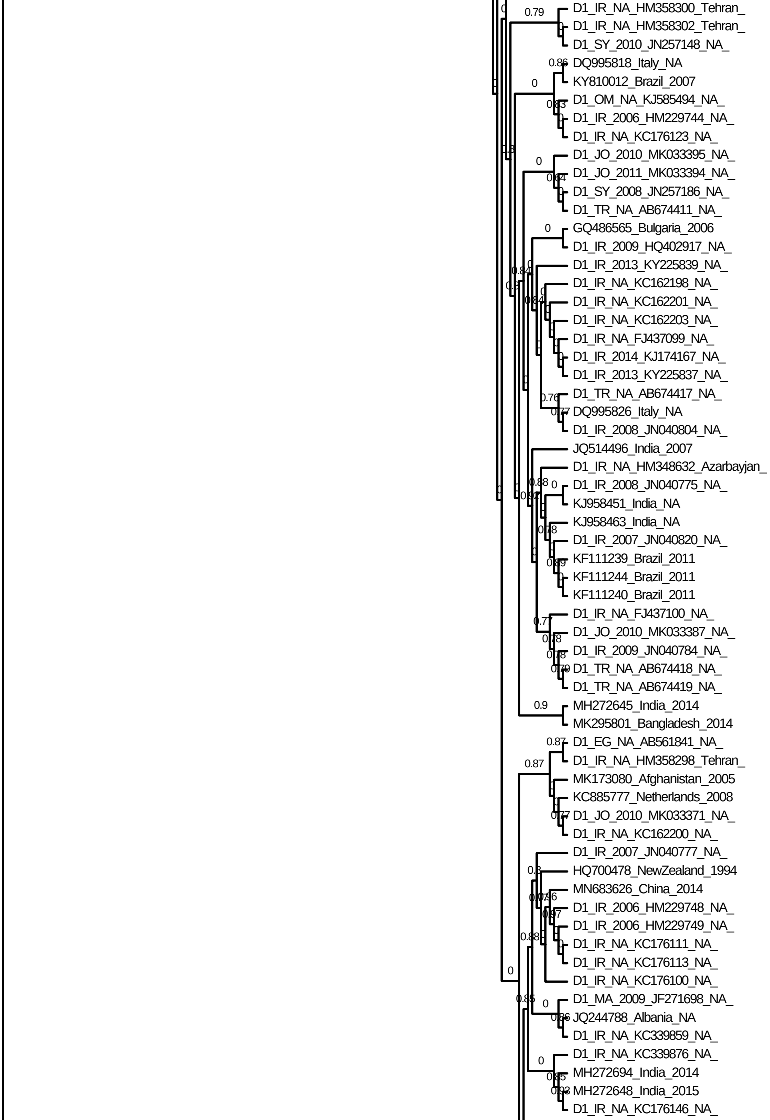
D1_IR_NA_HM358300_Tehran_
D1_IR_NA_HM358302_Tehran_
D1_SY_2010_JN257148_NA_
DQ995818_Italy_NA
KY810012_Brazil_2007
D1_OM_NA_KJ585494_NA_
D1_IR_2006_HM229744_NA_
D1_IR_NA_KC176123_NA_
D1_JO_2010_MK033395_NA_
D1_JO_2011_MK033394_NA_
D1_SY_2008_JN257186_NA_
D1_TR_NA_AB674411_NA_
GQ486565_Bulgaria_2006
D1_IR_2009_HQ402917_NA_
D1_IR_2013_KY225839_NA_
D1_IR_NA_KC162198_NA_
D1_IR_NA_KC162201_NA_
D1_IR_NA_KC162203_NA_
D1_IR_NA_FJ437099_NA_
D1_IR_2014_KJ174167_NA_
D1_IR_2013_KY225837_NA_
D1_TR_NA_AB674417_NA_
DQ995826_Italy_NA
D1_IR_2008_JN040804_NA_
JQ514496_India_2007
D1_IR_NA_HM348632_Azarbayjan_
D1_IR_2008_JN040775_NA_
KJ958451_India_NA
KJ958463_India_NA
D1_IR_2007_JN040820_NA_
KF111239_Brazil_2011
KF111244_Brazil_2011
KF111240_Brazil_2011
D1_IR_NA_FJ437100_NA_
D1_JO_2010_MK033387_NA_
D1_IR_2009_JN040784_NA_
D1_TR_NA_AB674418_NA_
D1_TR_NA_AB674419_NA_
MH272645_India_2014
MK295801_Bangladesh_2014
D1_EG_NA_AB561841_NA_
D1_IR_NA_HM358298_Tehran_
MK173080_Afghanistan_2005
KC885777_Netherlands_2008
D1_JO_2010_MK033371_NA_
D1_IR_NA_KC162200_NA_
D1_IR_2007_JN040777_NA_
HQ700478_NewZealand_1994
MN683626_China_2014
D1_IR_2006_HM229748_NA_
D1_IR_2006_HM229749_NA_
D1_IR_NA_KC176111_NA_
D1_IR_NA_KC176113_NA_
D1_IR_NA_KC176100_NA_
D1_MA_2009_JF271698_NA_
JQ244788_Albania_NA
D1_IR_NA_KC339859_NA_
D1_IR_NA_KC339876_NA_
MH272694_India_2014
MH272648_India_2015
D1_IR_NA_KC176146_NA_

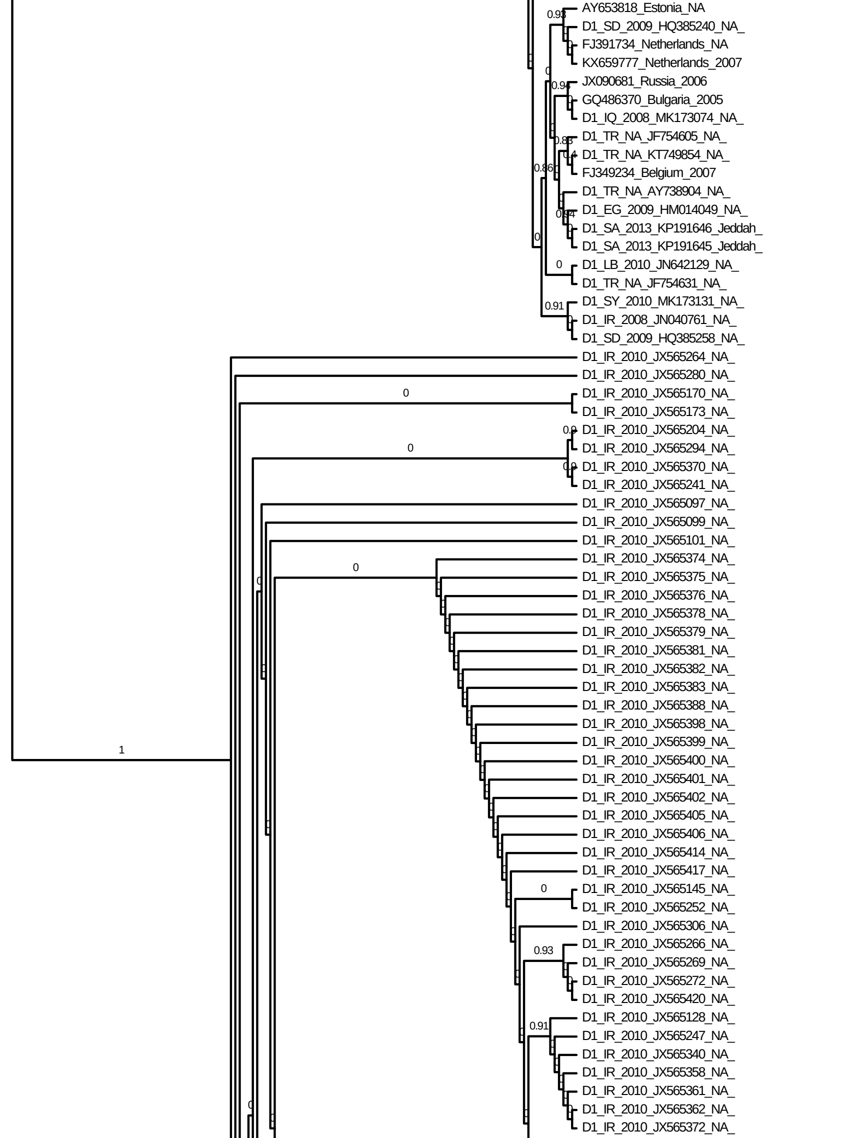
AY653818_Estonia_NA
D1_SD_2009_HQ385240_NA_
FJ391734_Netherlands_NA
KX659777_Netherlands_2007
JX090681_Russia_2006
GQ486370_Bulgaria_2005
D1_IQ_2008_MK173074_NA_
D1_TR_NA_JF754605_NA_
D1_TR_NA_KT749854_NA_
FJ349234_Belgium_2007
D1_TR_NA_AY738904_NA_
D1_EG_2009_HM014049_NA_
D1_SA_2013_KP191646_Jeddah_
D1_SA_2013_KP191645_Jeddah_
D1_LB_2010_JN642129_NA_
D1_TR_NA_JF754631_NA_
D1_SY_2010_MK173131_NA_
D1_IR_2008_JN040761_NA_
D1_SD_2009_HQ385258_NA_
D1_IR_2010_JX565264_NA_
D1_IR_2010_JX565280_NA_
D1_IR_2010_JX565170_NA_
D1_IR_2010_JX565173_NA_
D1_IR_2010_JX565204_NA_
D1_IR_2010_JX565294_NA_
D1_IR_2010_JX565370_NA_
D1_IR_2010_JX565241_NA_
D1_IR_2010_JX565097_NA_
D1_IR_2010_JX565099_NA_
D1_IR_2010_JX565101_NA_
D1_IR_2010_JX565374_NA_
D1_IR_2010_JX565375_NA_
D1_IR_2010_JX565376_NA_
D1_IR_2010_JX565378_NA_
D1_IR_2010_JX565379_NA_
D1_IR_2010_JX565381_NA_
D1_IR_2010_JX565382_NA_
D1_IR_2010_JX565383_NA_
D1_IR_2010_JX565388_NA_
D1_IR_2010_JX565398_NA_
D1_IR_2010_JX565399_NA_
D1_IR_2010_JX565400_NA_
D1_IR_2010_JX565401_NA_
D1_IR_2010_JX565402_NA_
D1_IR_2010_JX565405_NA_
D1_IR_2010_JX565406_NA_
D1_IR_2010_JX565414_NA_
D1_IR_2010_JX565417_NA_
D1_IR_2010_JX565145_NA_
D1_IR_2010_JX565252_NA_
D1_IR_2010_JX565306_NA_
D1_IR_2010_JX565266_NA_
D1_IR_2010_JX565269_NA_
D1_IR_2010_JX565272_NA_
D1_IR_2010_JX565420_NA_
D1_IR_2010_JX565128_NA_
D1_IR_2010_JX565247_NA_
D1_IR_2010_JX565340_NA_
D1_IR_2010_JX565358_NA_
D1_IR_2010_JX565361_NA_
D1_IR_2010_JX565362_NA_
D1_IR_2010_JX565372_NA_

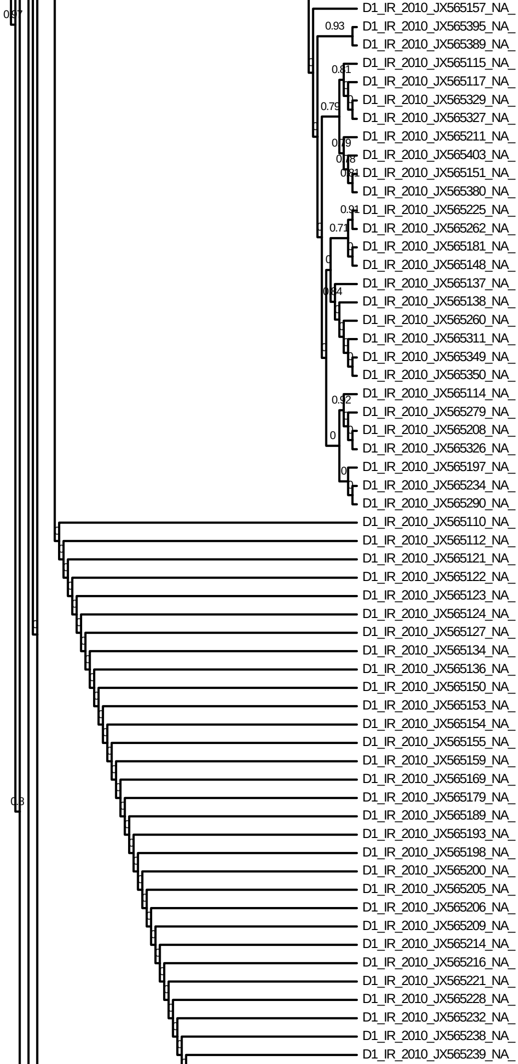

0.97
D1_IR_2010_JX565157_NA_
0.93
D1_IR_2010_JX565395_NA_
D1_IR_2010_JX565389_NA_
D1_IR_2010_JX565115_NA_
0.81
D1_IR_2010_JX565117_NA_
D1_IR_2010_JX565329_NA_
0.79
D1_IR_2010_JX565327_NA_
D1_IR_2010_JX565211_NA_
0.79
D1_IR_2010_JX565403_NA_
0.78
0.81
D1_IR_2010_JX565151_NA_
D1_IR_2010_JX565380_NA_
0.91
D1_IR_2010_JX565225_NA_
0.71
D1_IR_2010_JX565262_NA_
D1_IR_2010_JX565181_NA_
D1_IR_2010_JX565148_NA_
D1_IR_2010_JX565137_NA_
0.84
D1_IR_2010_JX565138_NA_
D1_IR_2010_JX565260_NA_
D1_IR_2010_JX565311_NA_
D1_IR_2010_JX565349_NA_
D1_IR_2010_JX565350_NA_
D1_IR_2010_JX565114_NA_
0.92
D1_IR_2010_JX565279_NA_
D1_IR_2010_JX565208_NA_
0
D1_IR_2010_JX565326_NA_
D1_IR_2010_JX565197_NA_
0
D1_IR_2010_JX565234_NA_
D1_IR_2010_JX565290_NA_
D1_IR_2010_JX565110_NA_
D1_IR_2010_JX565112_NA_
D1_IR_2010_JX565121_NA_
D1_IR_2010_JX565122_NA_
D1_IR_2010_JX565123_NA_
D1_IR_2010_JX565124_NA_
D1_IR_2010_JX565127_NA_
D1_IR_2010_JX565134_NA_
D1_IR_2010_JX565136_NA_
D1_IR_2010_JX565150_NA_
D1_IR_2010_JX565153_NA_
D1_IR_2010_JX565154_NA_
D1_IR_2010_JX565155_NA_
D1_IR_2010_JX565159_NA_
D1_IR_2010_JX565169_NA_
D1_IR_2010_JX565179_NA_
0.8
D1_IR_2010_JX565189_NA_
D1_IR_2010_JX565193_NA_
D1_IR_2010_JX565198_NA_
D1_IR_2010_JX565200_NA_
D1_IR_2010_JX565205_NA_
D1_IR_2010_JX565206_NA_
D1_IR_2010_JX565209_NA_
D1_IR_2010_JX565214_NA_
D1_IR_2010_JX565216_NA_
D1_IR_2010_JX565221_NA_
D1_IR_2010_JX565228_NA_
D1_IR_2010_JX565232_NA_
D1_IR_2010_JX565238_NA_
D1_IR_2010_JX565239_NA_

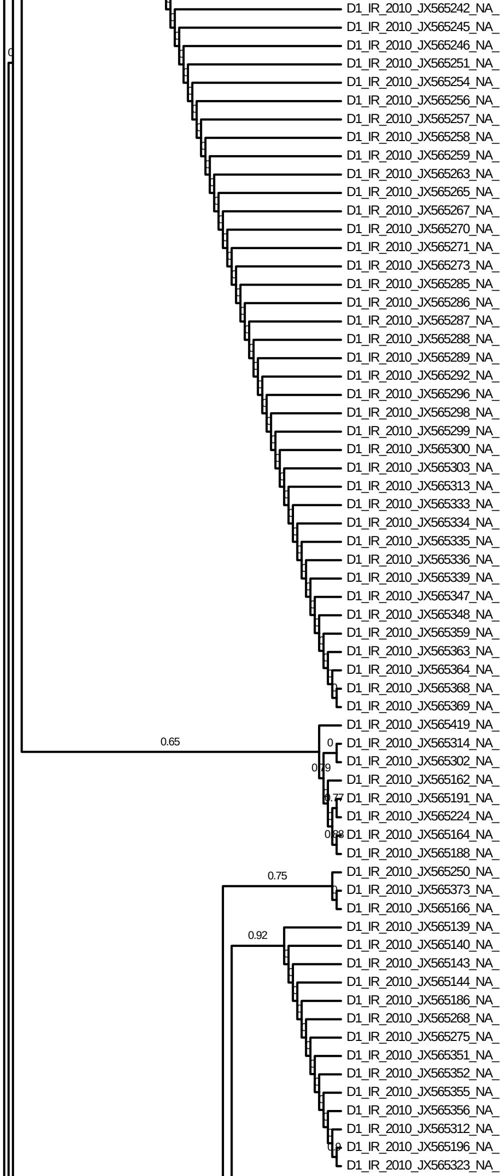
D1_IR_2010_JX565242_NA_
D1_IR_2010_JX565245_NA_
D1_IR_2010_JX565246_NA_
D1_IR_2010_JX565251_NA_
D1_IR_2010_JX565254_NA_
D1_IR_2010_JX565256_NA_
D1_IR_2010_JX565257_NA_
D1_IR_2010_JX565258_NA_
D1_IR_2010_JX565259_NA_
D1_IR_2010_JX565263_NA_
D1_IR_2010_JX565265_NA_
D1_IR_2010_JX565267_NA_
D1_IR_2010_JX565270_NA_
D1_IR_2010_JX565271_NA_
D1_IR_2010_JX565273_NA_
D1_IR_2010_JX565285_NA_
D1_IR_2010_JX565286_NA_
D1_IR_2010_JX565287_NA_
D1_IR_2010_JX565288_NA_
D1_IR_2010_JX565289_NA_
D1_IR_2010_JX565292_NA_
D1_IR_2010_JX565296_NA_
D1_IR_2010_JX565298_NA_
D1_IR_2010_JX565299_NA_
D1_IR_2010_JX565300_NA_
D1_IR_2010_JX565303_NA_
D1_IR_2010_JX565313_NA_
D1_IR_2010_JX565333_NA_
D1_IR_2010_JX565334_NA_
D1_IR_2010_JX565335_NA_
D1_IR_2010_JX565336_NA_
D1_IR_2010_JX565339_NA_
D1_IR_2010_JX565347_NA_
D1_IR_2010_JX565348_NA_
D1_IR_2010_JX565359_NA_
D1_IR_2010_JX565363_NA_
D1_IR_2010_JX565364_NA_
D1_IR_2010_JX565368_NA_
D1_IR_2010_JX565369_NA_
D1_IR_2010_JX565419_NA_
0.65
0
D1_IR_2010_JX565314_NA_
D1_IR_2010_JX565302_NA_
0.79
D1_IR_2010_JX565162_NA_
0.77
D1_IR_2010_JX565191_NA_
D1_IR_2010_JX565224_NA_
0.88
D1_IR_2010_JX565164_NA_
D1_IR_2010_JX565188_NA_
0.75
D1_IR_2010_JX565250_NA_
D1_IR_2010_JX565373_NA_
D1_IR_2010_JX565166_NA_
D1_IR_2010_JX565139_NA_
0.92
D1_IR_2010_JX565140_NA_
D1_IR_2010_JX565143_NA_
D1_IR_2010_JX565144_NA_
D1_IR_2010_JX565186_NA_
D1_IR_2010_JX565268_NA_
D1_IR_2010_JX565275_NA_
D1_IR_2010_JX565351_NA_
D1_IR_2010_JX565352_NA_
D1_IR_2010_JX565355_NA_
D1_IR_2010_JX565356_NA_
D1_IR_2010_JX565312_NA_
D1_IR_2010_JX565196_NA_
D1_IR_2010_JX565323_NA_

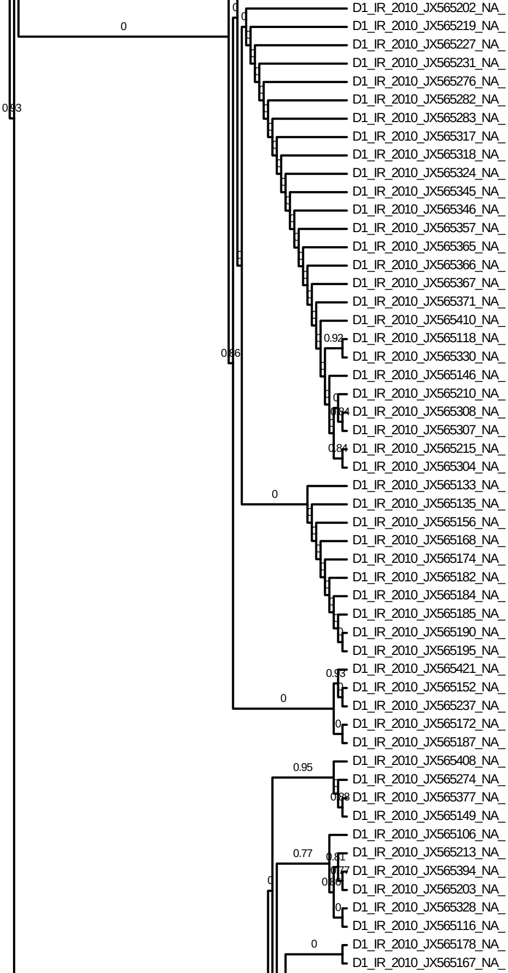

D1_IR_2010_JX565202_NA_
D1_IR_2010_JX565219_NA_
D1_IR_2010_JX565227_NA_
D1_IR_2010_JX565231_NA_
D1_IR_2010_JX565276_NA_
D1_IR_2010_JX565282_NA_
D1_IR_2010_JX565283_NA_
D1_IR_2010_JX565317_NA_
D1_IR_2010_JX565318_NA_
D1_IR_2010_JX565324_NA_
D1_IR_2010_JX565345_NA_
D1_IR_2010_JX565346_NA_
D1_IR_2010_JX565357_NA_
D1_IR_2010_JX565365_NA_
D1_IR_2010_JX565366_NA_
D1_IR_2010_JX565367_NA_
D1_IR_2010_JX565371_NA_
D1_IR_2010_JX565410_NA_
D1_IR_2010_JX565118_NA_
D1_IR_2010_JX565330_NA_
D1_IR_2010_JX565146_NA_
D1_IR_2010_JX565210_NA_
D1_IR_2010_JX565308_NA_
D1_IR_2010_JX565307_NA_
D1_IR_2010_JX565215_NA_
D1_IR_2010_JX565304_NA_
D1_IR_2010_JX565133_NA_
D1_IR_2010_JX565135_NA_
D1_IR_2010_JX565156_NA_
D1_IR_2010_JX565168_NA_
D1_IR_2010_JX565174_NA_
D1_IR_2010_JX565182_NA_
D1_IR_2010_JX565184_NA_
D1_IR_2010_JX565185_NA_
D1_IR_2010_JX565190_NA_
D1_IR_2010_JX565195_NA_
D1_IR_2010_JX565421_NA_
D1_IR_2010_JX565152_NA_
D1_IR_2010_JX565237_NA_
D1_IR_2010_JX565172_NA_
D1_IR_2010_JX565187_NA_
D1_IR_2010_JX565408_NA_
D1_IR_2010_JX565274_NA_
D1_IR_2010_JX565377_NA_
D1_IR_2010_JX565149_NA_
D1_IR_2010_JX565106_NA_
D1_IR_2010_JX565213_NA_
D1_IR_2010_JX565394_NA_
D1_IR_2010_JX565203_NA_
D1_IR_2010_JX565328_NA_
D1_IR_2010_JX565116_NA_
D1_IR_2010_JX565178_NA_
D1_IR_2010_JX565167_NA_

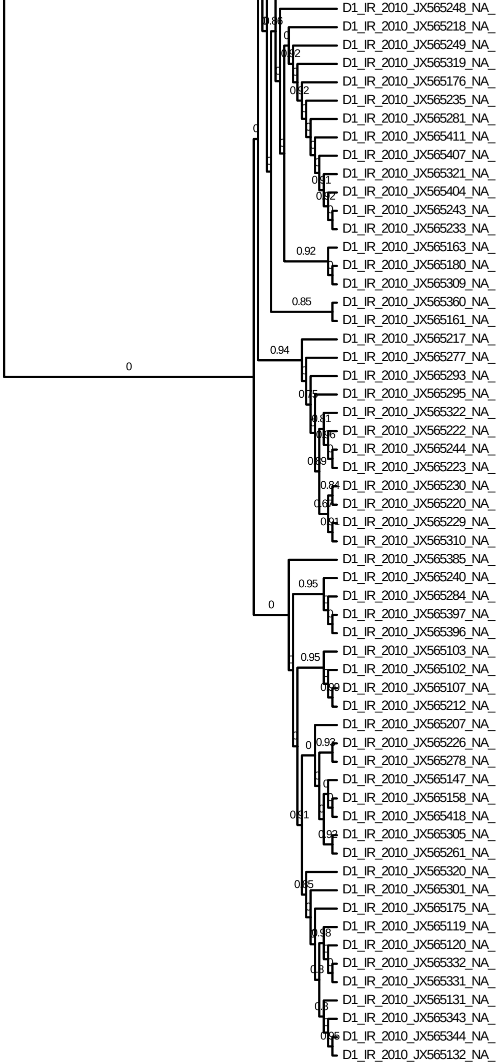

D1_IR_2010_JX565248_NA_
D1_IR_2010_JX565218_NA_
D1_IR_2010_JX565249_NA_
D1_IR_2010_JX565319_NA_
D1_IR_2010_JX565176_NA_
D1_IR_2010_JX565235_NA_
D1_IR_2010_JX565281_NA_
D1_IR_2010_JX565411_NA_
D1_IR_2010_JX565407_NA_
D1_IR_2010_JX565321_NA_
D1_IR_2010_JX565404_NA_
D1_IR_2010_JX565243_NA_
D1_IR_2010_JX565233_NA_
D1_IR_2010_JX565163_NA_
D1_IR_2010_JX565180_NA_
D1_IR_2010_JX565309_NA_
D1_IR_2010_JX565360_NA_
D1_IR_2010_JX565161_NA_
D1_IR_2010_JX565217_NA_
D1_IR_2010_JX565277_NA_
D1_IR_2010_JX565293_NA_
D1_IR_2010_JX565295_NA_
D1_IR_2010_JX565322_NA_
D1_IR_2010_JX565222_NA_
D1_IR_2010_JX565244_NA_
D1_IR_2010_JX565223_NA_
D1_IR_2010_JX565230_NA_
D1_IR_2010_JX565220_NA_
D1_IR_2010_JX565229_NA_
D1_IR_2010_JX565310_NA_
D1_IR_2010_JX565385_NA_
D1_IR_2010_JX565240_NA_
D1_IR_2010_JX565284_NA_
D1_IR_2010_JX565397_NA_
D1_IR_2010_JX565396_NA_
D1_IR_2010_JX565103_NA_
D1_IR_2010_JX565102_NA_
D1_IR_2010_JX565107_NA_
D1_IR_2010_JX565212_NA_
D1_IR_2010_JX565207_NA_
D1_IR_2010_JX565226_NA_
D1_IR_2010_JX565278_NA_
D1_IR_2010_JX565147_NA_
D1_IR_2010_JX565158_NA_
D1_IR_2010_JX565418_NA_
D1_IR_2010_JX565305_NA_
D1_IR_2010_JX565261_NA_
D1_IR_2010_JX565320_NA_
D1_IR_2010_JX565301_NA_
D1_IR_2010_JX565175_NA_
D1_IR_2010_JX565119_NA_
D1_IR_2010_JX565120_NA_
D1_IR_2010_JX565332_NA_
D1_IR_2010_JX565331_NA_
D1_IR_2010_JX565131_NA_
D1_IR_2010_JX565343_NA_
D1_IR_2010_JX565344_NA_
D1_IR_2010_JX565132_NA_

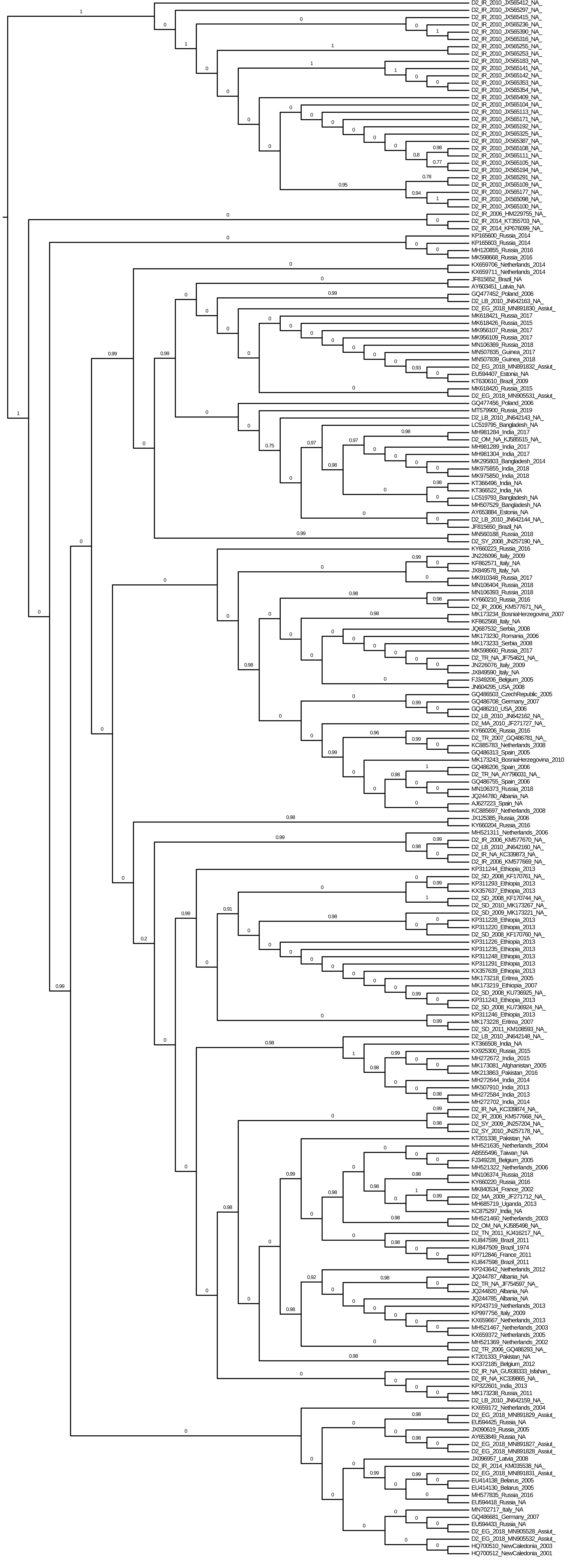
D2_IR_2010_JX565412_NA_
D2_IR_2010_JX565297_NA_
D2_IR_2010_JX565415_NA_
D2_IR_2010_JX565236_NA_
D2_IR_2010_JX565390_NA_
D2_IR_2010_JX565316_NA_
D2_IR_2010_JX565255_NA_
D2_IR_2010_JX565253_NA_
D2_IR_2010_JX565183_NA_
D2_IR_2010_JX565141_NA_
D2_IR_2010_JX565142_NA_
D2_IR_2010_JX565353_NA_
D2_IR_2010_JX565354_NA_
D2_IR_2010_JX565409_NA_
D2_IR_2010_JX565104_NA_
D2_IR_2010_JX565113_NA_
D2_IR_2010_JX565171_NA_
D2_IR_2010_JX565192_NA_
D2_IR_2010_JX565325_NA_
D2_IR_2010_JX565387_NA_
D2_IR_2010_JX565108_NA_
D2_IR_2010_JX565111_NA_
D2_IR_2010_JX565105_NA_
D2_IR_2010_JX565194_NA_
D2_IR_2010_JX565291_NA_
D2_IR_2010_JX565109_NA_
D2_IR_2010_JX565177_NA_
D2_IR_2010_JX565098_NA_
D2_IR_2010_JX565100_NA_
D2_IR_2006_HM229755_NA_
D2_IR_2014_KT355703_NA_
D2_IR_2014_KP676099_NA_
KP165600_Russia_2014
KP165603_Russia_2014
MH120855_Russia_2016
MK598668_Russia_2016
KX659706_Netherlands_2014
KX659711_Netherlands_2014
JF815652_Brazil_NA
AY603451_Latvia_NA
GQ477452_Poland_2006
D2_LB_2010_JN642163_NA_
D2_EG_2018_MN891830_Assiut_
MK618421_Russia_2017
MK618426_Russia_2015
MK956107_Russia_2017
MK956109_Russia_2017
MN106369_Russia_2018
MN507835_Guinea_2017
MN507839_Guinea_2018
D2_EG_2018_MN891832_Assiut_
EU594407_Estonia_NA
KT630610_Brazil_2009
MK618420_Russia_2015
D2_EG_2018_MN905531_Assiut_
GQ477456_Poland_2006
MT579900_Russia_2019
D2_LB_2010_JN642143_NA_
LC519795_Bangladesh_NA
MH981284_India_2017
D2_OM_NA_KJ585515_NA_
MH981289_India_2017
MH981304_India_2017
MK295803_Bangladesh_2014
MK975855_India_2018
MK975850_India_2018
KT366496_India_NA
KT366522_India_NA
LC519793_Bangladesh_NA
MH507529_Bangladesh_NA
AY653884_Estonia_NA
D2_LB_2010_JN642144_NA_
JF815650_Brazil_NA
MN560188_Russia_2018
D2_SY_2008_JN257190_NA_
KY660223_Russia_2016
JN226096_Italy_2009
KF862571_Italy_NA
JX849578_Italy_NA
MK910348_Russia_2017
MN106404_Russia_2018
MN106393_Russia_2018
KY660210_Russia_2016
D2_IR_2006_KM577671_NA_
MK173234_BosniaHerzegovina_2007
KF862568_Italy_NA
JQ687532_Serbia_2008
MK173230_Romania_2006
MK173233_Serbia_2008
MK598660_Russia_2017
D2_TR_NA_JF754621_NA_
JN226076_Italy_2009
JX849590_Italy_NA
FJ349206_Belgium_2005
JN604295_USA_2008
GQ486503_CzechRepublic_2005
GQ486708_Germany_2007
GQ486210_USA_2006
D2_LB_2010_JN642162_NA_
D2_MA_2010_JF271727_NA_
KY660206_Russia_2016
D2_TR_2007_GQ486781_NA_
KC885783_Netherlands_2008
GQ486313_Spain_2005
MK173243_BosniaHerzegovina_2010
GQ486206_Spain_2006
D2_TR_NA_AY796031_NA_
GQ486755_Spain_2006
MN106373_Russia_2018
JQ244780_Albania_NA
AJ627223_Spain_NA
KC885697_Netherlands_2008
JX125385_Russia_2006
KY660204_Russia_2016
MH521311_Netherlands_2006
D2_IR_2006_KM577670_NA_
D2_LB_2010_JN642160_NA_
D2_IR_NA_KC339873_NA_
D2_IR_2006_KM577669_NA_
KP311244_Ethiopia_2013
D2_SD_2008_KF170761_NA_
KP311293_Ethiopia_2013
KX357637_Ethiopia_2013
D2_SD_2008_KF170744_NA_
D2_SD_2010_MK173267_NA_
D2_SD_2009_MK173221_NA_
KP311228_Ethiopia_2013
KP311220_Ethiopia_2013
D2_SD_2008_KF170760_NA_
KP311226_Ethiopia_2013
KP311235_Ethiopia_2013
KP311248_Ethiopia_2013
KP311291_Ethiopia_2013
KX357639_Ethiopia_2013
MK173218_Eritrea_2005
MK173219_Ethiopia_2007
D2_SD_2008_KU736925_NA_
KP311243_Ethiopia_2013
D2_SD_2008_KU736924_NA_
KP311246_Ethiopia_2013
MK173228_Eritrea_2007
D2_SD_2011_KM108593_NA_
D2_LB_2010_JN642148_NA_
KT366508_India_NA
KX925300_Russia_2015
MH272672_India_2015
MK173081_Afghanistan_2005
MK213863_Pakistan_2016
MH272644_India_2014
MK507910_India_2013
MH272584_India_2013
MH272702_India_2014
D2_IR_NA_KC339874_NA_
D2_IR_2006_KM577668_NA_
D2_SY_2009_JN257204_NA_
D2_SY_2010_JN257178_NA_
KT201338_Pakistan_NA
MH521635_Netherlands_2004
AB555496_Taiwan_NA
FJ349228_Belgium_2005
MH521322_Netherlands_2006
MN106374_Russia_2018
KY660220_Russia_2016
MK840534_France_2002
D2_MA_2009_JF271712_NA_
MH685719_Uganda_2013
KC875297_India_NA
MH521460_Netherlands_2003
D2_OM_NA_KJ585498_NA_
D2_TN_2011_KJ416217_NA_
KU847599_Brazil_2011
KU847509_Brazil_1974
KP712846_France_2011
KU847598_Brazil_2011
KP243642_Netherlands_2012
JQ244787_Albania_NA
D2_TR_NA_JF754597_NA_
JQ244820_Albania_NA
JQ244785_Albania_NA
KP243719_Netherlands_2013
KP997756_Italy_2009
KX659667_Netherlands_2013
MH521467_Netherlands_2003
KX659372_Netherlands_2005
MH521369_Netherlands_2002
D2_TR_2006_GQ486293_NA_
KT201333_Pakistan_NA
KX372185_Belgium_2012
D2_IR_NA_GU938333_Isfahan_
D2_IR_NA_KC339865_NA_
KP322601_India_2013
MK173238_Russia_2011
D2_LB_2010_JN642159_NA_
KX659172_Netherlands_2004
D2_EG_2018_MN891829_Assiut_
EU594425_Russia_NA
JX090619_Russia_2005
AY653849_Russia_NA
D2_EG_2018_MN891827_Assiut_
D2_EG_2018_MN891828_Assiut_
JX096957_Latvia_2008
D2_IR_2014_KM035538_NA_
D2_EG_2018_MN891831_Assiut_
EU414138_Belarus_2005
EU414130_Belarus_2005
MH577835_Russia_2016
EU594418_Russia_NA
MN702717_Italy_NA
GQ486681_Germany_2007
EU594433_Russia_NA
D2_EG_2018_MN905528_Assiut_
D2_EG_2018_MN905532_Assiut_
HQ700510_NewCaledonia_2003
HQ700512_NewCaledonia_2001

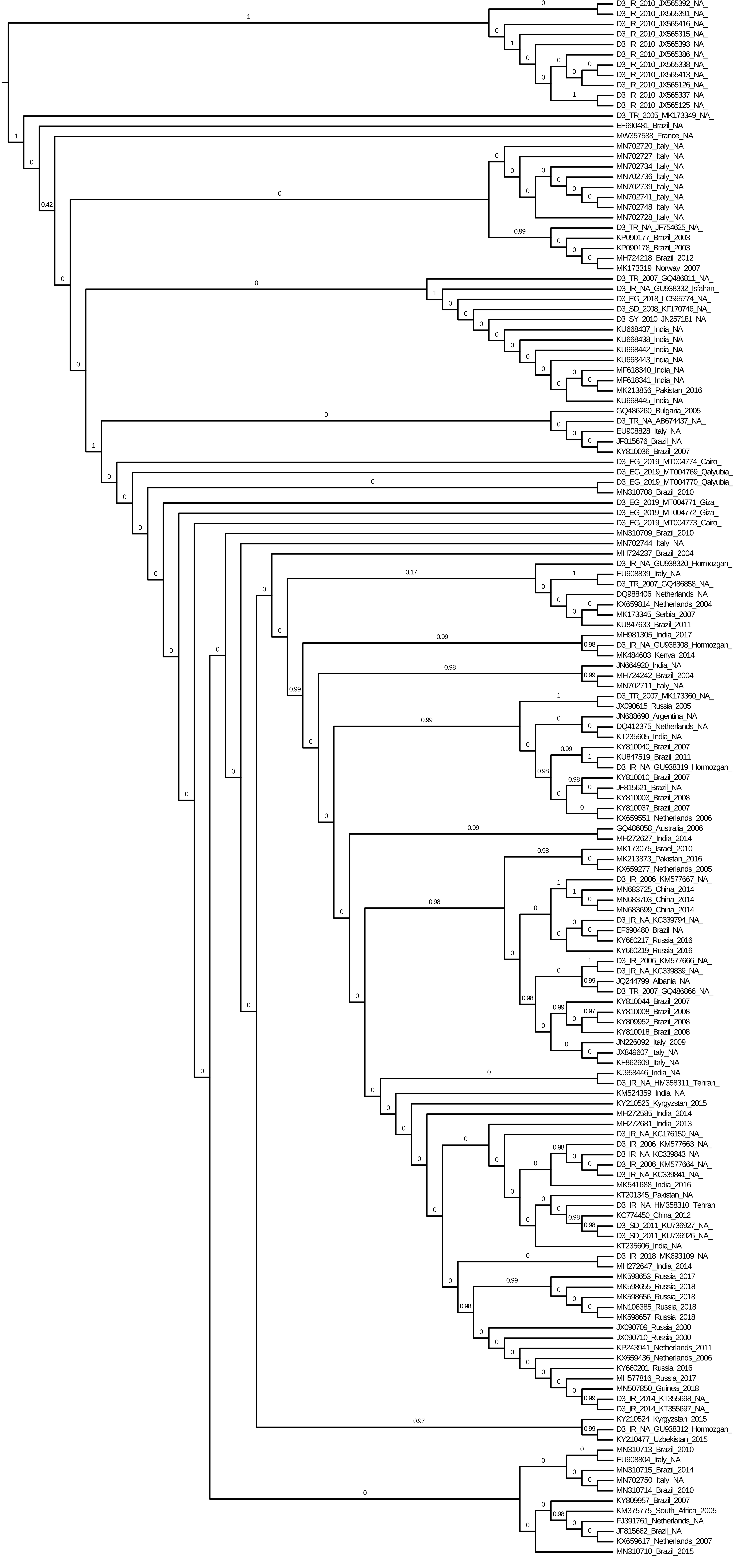
D3_IR_2010_JX565392_NA_
D3_IR_2010_JX565391_NA_
D3_IR_2010_JX565416_NA_
D3_IR_2010_JX565315_NA_
D3_IR_2010_JX565393_NA_
D3_IR_2010_JX565386_NA_
D3_IR_2010_JX565338_NA_
D3_IR_2010_JX565413_NA_
D3_IR_2010_JX565126_NA_
D3_IR_2010_JX565337_NA_
D3_IR_2010_JX565125_NA_
D3_TR_2005_MK173349_NA_
EF690481_Brazil_NA
MW357588_France_NA
MN702720_Italy_NA
MN702727_Italy_NA
MN702734_Italy_NA
MN702736_Italy_NA
MN702739_Italy_NA
MN702741_Italy_NA
MN702748_Italy_NA
MN702728_Italy_NA
D3_TR_NA_JF754625_NA_
KP090177_Brazil_2003
KP090178_Brazil_2003
MH724218_Brazil_2012
MK173319_Norway_2007
D3_TR_2007_GQ486811_NA_
D3_IR_NA_GU938332_Isfahan_
D3_EG_2018_LC595774_NA_
D3_SD_2008_KF170746_NA_
D3_SY_2010_JN257181_NA_
KU668437_India_NA
KU668438_India_NA
KU668442_India_NA
KU668443_India_NA
MF618340_India_NA
MF618341_India_NA
MK213856_Pakistan_2016
KU668445_India_NA
GQ486260_Bulgaria_2005
D3_TR_NA_AB674437_NA_
EU908828_Italy_NA
JF815676_Brazil_NA
KY810036_Brazil_2007
D3_EG_2019_MT004774_Cairo_
D3_EG_2019_MT004769_Qalyubia_
D3_EG_2019_MT004770_Qalyubia_
MN310708_Brazil_2010
D3_EG_2019_MT004771_Giza_
D3_EG_2019_MT004772_Giza_
D3_EG_2019_MT004773_Cairo_
MN310709_Brazil_2010
MN702744_Italy_NA
MH724237_Brazil_2004
D3_IR_NA_GU938320_Hormozgan_
EU908839_Italy_NA
D3_TR_2007_GQ486858_NA_
DQ988406_Netherlands_NA
KX659814_Netherlands_2004
MK173345_Serbia_2007
KU847633_Brazil_2011
MH981305_India_2017
D3_IR_NA_GU938308_Hormozgan_
MK484603_Kenya_2014
JN664920_India_NA
MH724242_Brazil_2004
MN702711_Italy_NA
D3_TR_2007_MK173360_NA_
JX090615_Russia_2005
JN688690_Argentina_NA
DQ412375_Netherlands_NA
KT235605_India_NA
KY810040_Brazil_2007
KU847519_Brazil_2011
D3_IR_NA_GU938319_Hormozgan_
KY810010_Brazil_2007
JF815621_Brazil_NA
KY810003_Brazil_2008
KY810037_Brazil_2007
KX659551_Netherlands_2006
GQ486058_Australia_2006
MH272627_India_2014
MK173075_Israel_2010
MK213873_Pakistan_2016
KX659277_Netherlands_2005
D3_IR_2006_KM577667_NA_
MN683725_China_2014
MN683703_China_2014
MN683699_China_2014
D3_IR_NA_KC339794_NA_
EF690480_Brazil_NA
KY660217_Russia_2016
KY660219_Russia_2016
D3_IR_2006_KM577666_NA_
D3_IR_NA_KC339839_NA_
JQ244799_Albania_NA
D3_TR_2007_GQ486866_NA_
KY810044_Brazil_2007
KY810008_Brazil_2008
KY809952_Brazil_2008
KY810018_Brazil_2008
JN226092_Italy_2009
JX849607_Italy_NA
KF862609_Italy_NA
KJ958446_India_NA
D3_IR_NA_HM358311_Tehran_
KM524359_India_NA
KY210525_Kyrgyzstan_2015
MH272585_India_2014
MH272681_India_2013
D3_IR_NA_KC176150_NA_
D3_IR_2006_KM577663_NA_
D3_IR_NA_KC339843_NA_
D3_IR_2006_KM577664_NA_
D3_IR_NA_KC339841_NA_
MK541688_India_2016
KT201345_Pakistan_NA
D3_IR_NA_HM358310_Tehran_
KC774450_China_2012
D3_SD_2011_KU736927_NA_
D3_SD_2011_KU736926_NA_
KT235606_India_NA
D3_IR_2018_MK693109_NA_
MH272647_India_2014
MK598653_Russia_2017
MK598655_Russia_2018
MK598656_Russia_2018
MN106385_Russia_2018
MK598657_Russia_2018
JX090709_Russia_2000
JX090710_Russia_2000
KP243941_Netherlands_2011
KX659436_Netherlands_2006
KY660201_Russia_2016
MH577816_Russia_2017
MN507850_Guinea_2018
D3_IR_2014_KT355698_NA_
D3_IR_2014_KT355697_NA_
KY210524_Kyrgyzstan_2015
D3_IR_NA_GU938312_Hormozgan_
KY210477_Uzbekistan_2015
MN310713_Brazil_2010
EU908804_Italy_NA
MN310715_Brazil_2014
MN702750_Italy_NA
MN310714_Brazil_2010
KY809957_Brazil_2007
KM375775_South_Africa_2005
FJ391761_Netherlands_NA
JF815662_Brazil_NA
KX659617_Netherlands_2007
MN310710_Brazil_2015

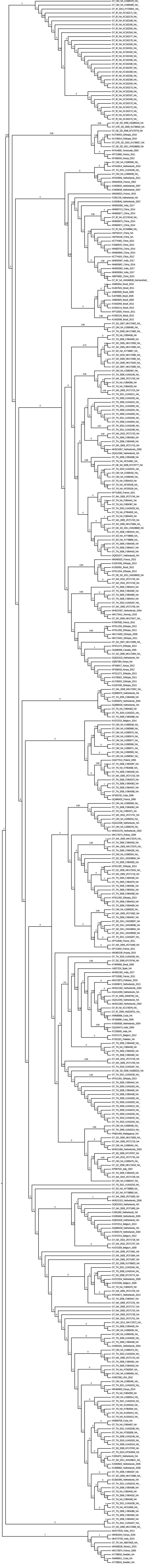

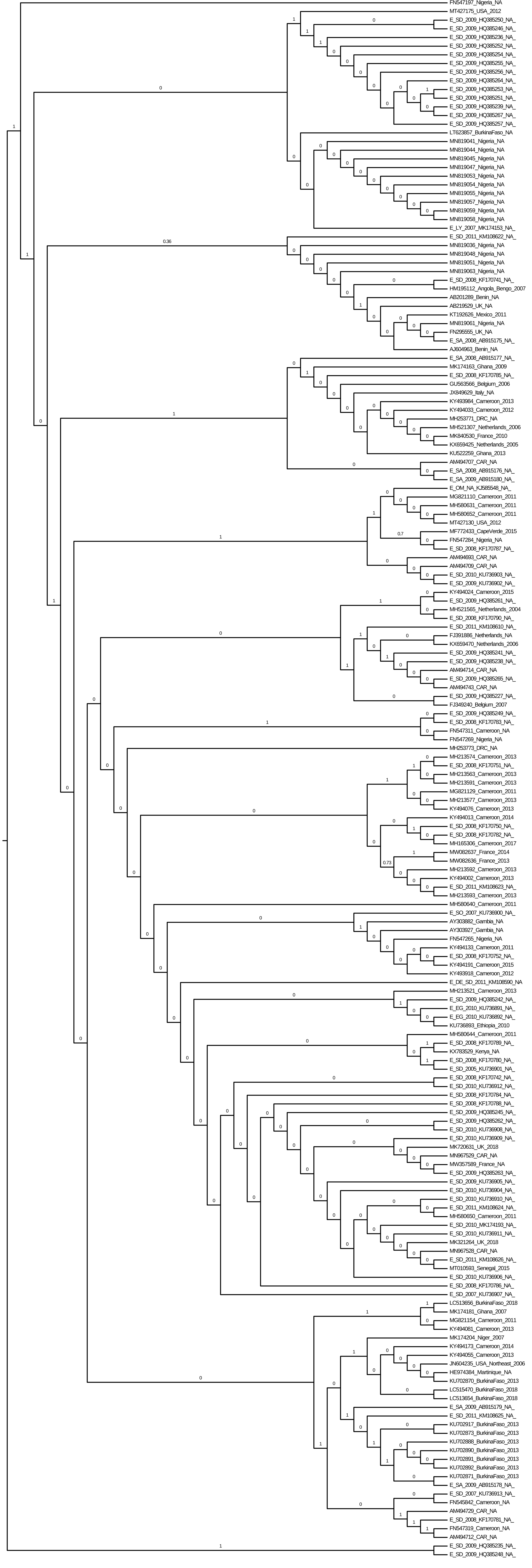
FN547197_Nigeria_NA
MT427175_USA_2012
E_SD_2009_HQ385250_NA_
E_SD_2009_HQ385246_NA_
E_SD_2009_HQ385236_NA_
E_SD_2009_HQ385252_NA_
E_SD_2009_HQ385254_NA_
E_SD_2009_HQ385255_NA_
E_SD_2009_HQ385256_NA_
E_SD_2009_HQ385264_NA_
E_SD_2009_HQ385253_NA_
E_SD_2009_HQ385251_NA_
E_SD_2009_HQ385239_NA_
E_SD_2009_HQ385267_NA_
E_SD_2009_HQ385257_NA_
LT623857_BurkinaFaso_NA
MN819041_Nigeria_NA
MN819044_Nigeria_NA
MN819045_Nigeria_NA
MN819047_Nigeria_NA
MN819053_Nigeria_NA
MN819054_Nigeria_NA
MN819055_Nigeria_NA
MN819057_Nigeria_NA
MN819059_Nigeria_NA
MN819058_Nigeria_NA
E_LY_2007_MK174153_NA_
E_SD_2011_KM108622_NA_
MN819036_Nigeria_NA
MN819048_Nigeria_NA
MN819051_Nigeria_NA
MN819063_Nigeria_NA
E_SD_2008_KF170741_NA_
HM195112_Angola_Bengo_2007
AB201289_Benin_NA
AB219529_UK_NA
KT192626_Mexico_2011
MN819061_Nigeria_NA
FN295555_UK_NA
E_SA_2008_AB915175_NA_
AJ604963_Benin_NA
E_SA_2008_AB915177_NA_
MK174163_Ghana_2009
E_SD_2008_KF170785_NA_
GU563566_Belgium_2006
JX849629_Italy_NA
KY493984_Cameroon_2013
KY494033_Cameroon_2012
MH253771_DRC_NA
MH521307_Netherlands_2006
MK840530_France_2010
KX659425_Netherlands_2005
KU522259_Ghana_2013
AM494707_CAR_NA
E_SA_2008_AB915176_NA_
E_SA_2009_AB915180_NA_
E_OM_NA_KJ585548_NA_
MG821110_Cameroon_2011
MH580631_Cameroon_2011
MH580652_Cameroon_2011
MT427130_USA_2012
MF772433_CapeVerde_2015
FN547284_Nigeria_NA
E_SD_2008_KF170787_NA_
AM494693_CAR_NA
AM494709_CAR_NA
E_SD_2010_KU736903_NA_
E_SD_2009_KU736902_NA_
KY494024_Cameroon_2015
E_SD_2009_HQ385261_NA_
MH521565_Netherlands_2004
E_SD_2008_KF170790_NA_
E_SD_2011_KM108610_NA_
FJ391886_Netherlands_NA
KX659470_Netherlands_2006
E_SD_2009_HQ385241_NA_
E_SD_2009_HQ385238_NA_
AM494714_CAR_NA
E_SD_2009_HQ385265_NA_
AM494743_CAR_NA
E_SD_2009_HQ385227_NA_
FJ349240_Belgium_2007
E_SD_2009_HQ385249_NA_
E_SD_2008_KF170783_NA_
FN547311_Cameroon_NA
FN547269_Nigeria_NA
MH253773_DRC_NA
MH213574_Cameroon_2013
E_SD_2008_KF170751_NA_
MH213563_Cameroon_2013
MH213591_Cameroon_2013
MG821129_Cameroon_2011
MH213577_Cameroon_2013
KY494076_Cameroon_2013
KY494013_Cameroon_2014
E_SD_2008_KF170750_NA_
E_SD_2008_KF170782_NA_
MH165306_Cameroon_2017
MW082637_France_2014
MW082636_France_2013
MH213592_Cameroon_2013
KY494002_Cameroon_2013
E_SD_2011_KM108623_NA_
MH213593_Cameroon_2013
MH580640_Cameroon_2011
E_SO_2007_KU736900_NA_
AY303882_Gambia_NA
AY303927_Gambia_NA
FN547265_Nigeria_NA
KY494133_Cameroon_2011
E_SD_2008_KF170752_NA_
KY494191_Cameroon_2015
KY493918_Cameroon_2012
E_DE_SD_2011_KM108590_NA
MH213521_Cameroon_2013
E_SD_2009_HQ385242_NA_
E_EG_2010_KU736891_NA_
E_EG_2010_KU736892_NA_
KU736893_Ethiopia_2010
MH580644_Cameroon_2011
E_SD_2008_KF170789_NA_
KX783529_Kenya_NA
E_SD_2008_KF170780_NA_
E_SD_2005_KU736901_NA_
E_SD_2008_KF170742_NA_
E_SD_2010_KU736912_NA_
E_SD_2008_KF170784_NA_
E_SD_2008_KF170788_NA_
E_SD_2009_HQ385245_NA_
E_SD_2009_HQ385262_NA_
E_SD_2010_KU736908_NA_
E_SD_2010_KU736909_NA_
MK720631_UK_2018
MN967529_CAR_NA
MW357589_France_NA
E_SD_2009_HQ385263_NA_
E_SD_2009_KU736905_NA_
E_SD_2010_KU736904_NA_
E_SD_2010_KU736910_NA_
E_SD_2011_KM108624_NA_
MH580650_Cameroon_2011
E_SD_2010_MK174193_NA_
E_SD_2010_KU736911_NA_
MK321264_UK_2018
MN967528_CAR_NA
E_SD_2011_KM108626_NA_
MT010593_Senegal_2015
E_SD_2010_KU736906_NA_
E_SD_2008_KF170786_NA_
E_SD_2007_KU736907_NA_
LC513656_BurkinaFaso_2018
MK174181_Ghana_2007
MG821154_Cameroon_2011
KY494081_Cameroon_2013
MK174204_Niger_2007
KY494173_Cameroon_2014
KY494055_Cameroon_2013
JN604235_USA_Northeast_2006
HE974384_Martinique_NA
KU702870_BurkinaFaso_2013
LC515470_BurkinaFaso_2018
LC513654_BurkinaFaso_2018
E_SA_2009_AB915179_NA_
E_SD_2011_KM108625_NA_
KU702917_BurkinaFaso_2013
KU702873_BurkinaFaso_2013
KU702888_BurkinaFaso_2013
KU702890_BurkinaFaso_2013
KU702891_BurkinaFaso_2013
KU702892_BurkinaFaso_2013
KU702871_BurkinaFaso_2013
E_SA_2009_AB915178_NA_
E_SD_2007_KU736913_NA_
FN545842_Cameroon_NA
AM494729_CAR_NA
E_SD_2008_KF170781_NA_
FN547319_Cameroon_NA
AM494712_CAR_NA
E_SD_2009_HQ385235_NA_
E_SD_2009_HQ385248_NA_
